# Mental disorders following COVID-19 and other epidemics: a systematic review and meta-analysis

**DOI:** 10.1038/s41398-022-01946-6

**Published:** 2022-05-17

**Authors:** Candi M. C. Leung, Margaret K. Ho, Alina A. Bharwani, Hugo Cogo-Moreira, Yishan Wang, Mathew S. C. Chow, Xiaoyan Fan, Sandro Galea, Gabriel M. Leung, Michael Y. Ni

**Affiliations:** 1 School of Public Health, LKS Faculty of Medicine, The University of Hong Kong, Hong Kong, Special Administrative Region China; 2 Department of Psychiatry, Universidade Federal de São Paulo, São Paulo, Brazil; 3 School of Public Health, Boston University, Boston, MA USA; 4 World Health Organization Collaborating Centre for Infectious Disease Epidemiology and Control, School of Public Health, The University of Hong Kong, Hong Kong, Special Administrative Region China; 5 The State Key Laboratory of Brain and Cognitive Sciences, The University of Hong Kong, Hong Kong, Special Administrative Region China; 6 Healthy High Density Cities Lab, HKUrbanLab, The University of Hong Kong, Hong Kong, Special Administrative Region China

**Keywords:** Psychiatric disorders, Depression

## Abstract

COVID-19 has imposed a very substantial direct threat to the physical health of those infected, although the corollary impact on mental health may be even more burdensome. Here we focus on assessing the mental health impact of COVID-19 and of other epidemics in the community. We searched five electronic databases until December 9, 2020, for all peer-reviewed original studies reporting any prevalence or correlates of mental disorders in the general population following novel epidemics in English, Chinese or Portuguese. We synthesised prevalence estimates from probability samples during COVID-19 and past epidemics. The meta-analytical effect size was the prevalence of relevant outcomes, estimated via random-effects model. *I*^2^ statistics, Doi plots and the LFK index were used to examine heterogeneity and publication bias. This study is pre-registered with PROSPERO, CRD42020179105. We identified 255 eligible studies from 50 countries on: COVID-19 (*n* = 247 studies), severe acute respiratory syndrome (SARS; *n* = 5), Ebola virus disease (*n* = 2), and 1918 influenza (*n* = 1). During COVID-19, we estimated the point prevalence for probable anxiety (20.7%, 95% CI 12.9–29.7), probable depression (18.1%, 13.0–23.9), and psychological distress (13.0%, 0–34.1). Correlates for poorer mental health include female sex, lower income, pre-existing medical conditions, perceived risk of infection, exhibiting COVID-19-like symptoms, social media use, financial stress, and loneliness. Public trust in authorities, availability of accurate information, adoption of preventive measures and social support were associated with less morbidity. The mental health consequences of COVID-19 and other epidemics could be comparable to major disasters and armed conflicts. The considerable heterogeneity in our analysis indicates that more random samples are needed. Health-care professionals should be vigilant of the psychological toll of epidemics, including among those who have not been infected.

## Introduction

COVID-19 has disrupted most aspects of daily life and resulted in wide-ranging psychosocial and economic stressors including fear of disease, loss of loved ones, lockdowns, social isolation, school closures, and economic recession [1, 2]. Prior systematic reviews and meta-analyses on COVID-19 have summarised the early findings on mental health available from mostly convenience samples [3–9]. Here, we focus on the prevalence of mental health disorders in probability samples and those with pre-pandemic mental health measures [10].

Mental health consequences of novel epidemics have been examined since the 1918 influenza pandemic to more recently Ebola virus disease and past coronavirus epidemics: severe acute respiratory syndrome (SARS) and Middle East respiratory syndrome (MERS) [2]. Research on COVID-19 and future epidemics could benefit from drawing upon the decades of epidemics-related literature (e.g. study design, potential findings) [11–13]. Accordingly, we conducted a systematic review and meta-analysis of the impact of COVID-19 and past epidemics on population mental health.

## Methods

We pre-registered the study protocol on PROSPERO (CRD42020179105) [14] and followed the PRISMA guideline.

### Search strategy

We searched PubMed, PsycINFO, Embase, CINAHL Plus, and Web of Science from their inception until December 9, 2020. Detailed search strategy and selection criteria are shown in Tables S1 and S2. The scope of our review was the prevalence or correlates of mental disorders in the general population exposed to any droplet-transmissible and airborne-transmissible viral outbreaks, which included novel epidemics of influenza viruses, Ebola virus and coronavirus [13]. Examples were COVID-19, Ebola virus disease, MERS, avian influenza A(H7N9), pandemic influenza A(H1N1), avian influenza A(H5N1), SARS and 1918 influenza [13]. Due to the unprecedented number of COVID-19 studies [15], we narrowed our original inclusion criteria to focus on mental disorders, which may have more clinical utility than the level of symptoms during population shocks [16, 17]. Outcomes were specific mental disorders or clinically significant level of mental distress (hereafter referred to as “psychological distress”), which were assessed by clinician interviews, diagnostic interviews (e.g. SCID, CIDI), or screening tools validated against clinician/diagnostic interviews (e.g. Patient Health Questionnaire-9, Generalized Anxiety Disorder Scale-7, General Health Questionnaire-12). Other transdiagnostic outcomes (e.g. insomnia) were excluded, except suicidality which has been identified as an urgent research priority [18]. Definitions and standardised measures of these outcomes are summarised in Table S3. Only original research studies published in peer-reviewed journals were eligible. In addition to articles in English, we included articles in Chinese and Portuguese based on the languages that known by the authors. We excluded studies focusing on subgroups (e.g. university students), conference abstracts, qualitative studies and modelling studies. We searched the reference lists of the identified studies, grey literature, Google Scholar, and previous review articles to identify additional studies.

### Data extraction

**Fig. 1:**
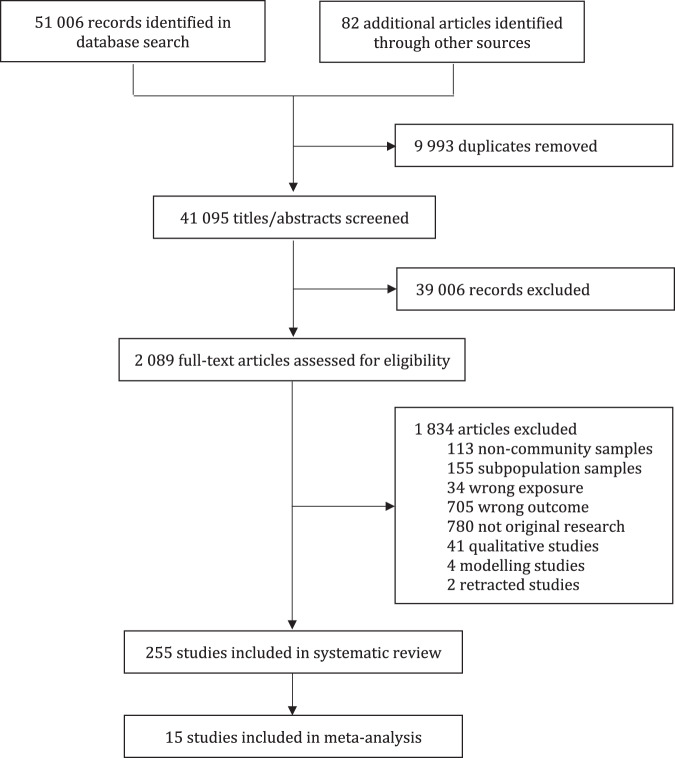
PRISMA flowchart.

Six authors (CML, MKH, AAB, YW, MSC, and XF) worked independently in pairs for screening, data extraction, and evidence grading (Fig. 1) after training and concordance assessment. Any disagreements were first resolved by consensus, then by a third author. With a piloted form, we extracted data for setting, disease, phase of epidemic, study design, survey method, sampling, participation rate, sample size, age range of sample, measures, prevalence and correlates of outcomes, among others. We contacted authors for missing or incomplete prevalence data.

### Evidence grading

We used the Newcastle-Ottawa Scale (NOS) recommended by the Cochrane collaboration to appraise study quality [19]. The NOS was modified for assessing cross-sectional studies with reference to previous adaptations (Table S4) [12, 20–22]. Total NOS scores ranged from 0 to 9 for longitudinal and case-control studies and from 0 to 6 for cross-sectional studies. Study quality was classified as low (0–3), medium (4–6), and high (7–9) [12]. We evaluated the certainty of evidence for each outcome from very low, low, moderate, to high using the Grading of Recommendations, Assessment, Development and Evaluations (GRADE) [23].

### Data synthesis

We prioritised methodologically sound studies in our data synthesis, given the large number of studies in the extant literature. Meta-analysis was added to this review, because we had identified a number of new probability samples after the PROSPERO registration. We synthesised prevalence estimates only from probability samples, which were recruited via any random sampling procedures (e.g., random-digit-dialling, address-based sampling) [10]. Correlates were summarised only when they were identified via multivariable analyses [24]. These included those consistently identified by at least two studies, and some others that were uniquely reported but may be potentially important predictors of mental disorders during epidemics. We tabulated the results by outcomes and epidemics if applicable. Meta-analysis was conducted using the double arcsine transformation [25]. We used a random-effects model that considers sample size and study quality [26]. The meta-analytical effect size was the pooled prevalence of relevant outcomes with 95% CI. We selected only studies comparable in terms of phase of epidemic (e.g. during, after epidemic) to reduce heterogeneity. In case of duplicate data, we included only the one with largest sample size. For cohort studies, we included only the first survey after the outbreak. Raw data for meta-analysis is provided in Table S5. Forest plots with *I*^2^ statistics were used to examine any study heterogeneity. Given the small number of probability samples, Doi plots and Luis Furuya-Kanamori (LFK) index were used to detect publication bias where applicable [27]. All statistical analyses were done by MetaXL 5.3 [28].

## Results

### Study characteristics

**Fig. 2:**
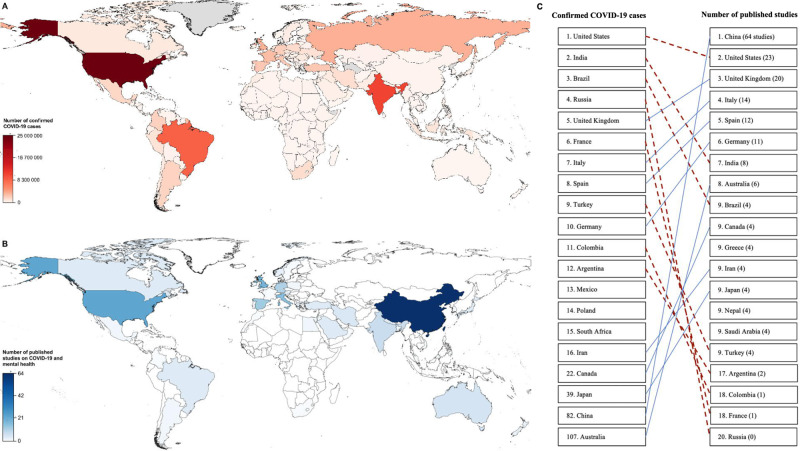
**A** Number of confirmed Coronavirus Disease 2019 (COVID-19) cases as of January 21, 2021. **B** Number of published studies on COVID-19 and mental health as of December 9, 2020. **C** Ranking of countries based on panels (**A**) confirmed COVID-19 cases and (**B**) number of studies on COVID-19 and mental health. Data source: Center for Systems Science and Engineering at Johns Hopkins University. Grey regions indicate regions with no available data.

Of the 41,095 unique records screened, we identified 255 eligible studies that examined prevalence or correlates of mental disorders or suicidality during novel epidemics (Fig. 1). These included 247 studies on COVID-19 (97%) involving over 1.2 million participants and 48 countries (Table 1). A quarter of COVID-19 studies were conducted in China (*n* = 64), more than other individual countries worldwide (Fig. 2, Table S6). Nearly 90% (*n* = 220) of COVID-19 studies had used convenience samples or opt-in online panels, compared to 12.5% (*n* = 1) of studies on past epidemics.

**Table 1:** Study characteristics of published studies on novel epidemics and mental health.

	Number of studies (%) (*n* = 255)
Disease	
Coronavirus	252 (98.8)
Coronavirus Disease 2019	247 (96.9)
Severe acute respiratory syndrome	5 (2.0)
Ebola virus disease	2 (0.8)
1918 influenza	1 (0.4)
Study design	
Longitudinal	14 (5.5)
Time series	3 (1.2)
Case-control	4 (1.6)
Serial cross-sectional	10 (3.9)
Cross-sectional	224 (87.8)
Mental health outcomes^a^	
Depression	148 (58.0)
Anxiety	133 (52.2)
Post-traumatic stress disorder	55 (21.6)
Psychological distress	54 (21.2)
Suicidality	11 (4.3)
Alcohol use disorder	5 (2.0)
Acute stress disorder	2 (0.8)
Obsessive-compulsive disorder	2 (0.8)
Agoraphobia	1 (0.4)
Panic disorder	1 (0.4)
Social phobia	1 (0.4)

^a^The number of studies may exceed 255 as some studies examined more than one outcome.

For all epidemics, we identified 33 studies (13%) that assessed prevalence of mental disorders or suicidality in probability samples or whole populations (Table S7) [29–61]. Nine of these studies reported pre-epidemic baseline prevalence of mental disorders [33, 35–37, 47, 51, 53, 55, 58], but six of these relied on other samples for baseline data. The remaining three studies examined psychological distress before and during COVID-19 using the same panel of individuals in the UK Household Longitudinal Study (Table 2) [47, 53, 55]. We included 15 studies for meta-analysis of prevalence estimates during epidemics (Table S5).

**Table 2:** Prevalence of mental health outcomes during and after novel epidemics in probability samples of general population.

Study	Setting	Disease	Response rate reported	Weighting applied	Sample size	Baseline prevalence before the epidemic^a^	Prevalence during or after the epidemic (95% CI)
*Anxiety*							
Bruine de Bruin 2020 [29]	United States	COVID-19	79%	Yes	6666	–	15.5%^b,c^
Choi et al. 2020 [30]	Hong Kong	COVID-19	64.6%	–	500	–	14.0%
Holingue et al. 2020 [31]	United States	COVID-19	63%	Yes	5065	–	14.7%^c^
Qian et al. 2020 [32]	China	COVID-19	13.8%	Yes	Wuhan: 510, Shanghai: 501	–	Wuhan: 32.8%; Shanghai: 20.5%
Twenge et al. 2020 [33]	United States	COVID-19	–	Yes	39,447–119,897	8.2%	T1: 30.8%, T2: 30.0%, T3: 28.2%, T4: 29.4%^d^
Zhao et al. 2020 [35]	Hong Kong	COVID-19	61.3%	Yes	1501	T1: 11.3%, T2: 9.3%	15.8%^e^
*Depression*							
Bruine de Bruin 2020 [29]	United States	COVID-19	79%	Yes	6666	–	10.3%^b,c^
Choi et al. 2020 [30]	Hong Kong	COVID-19	64.6%	–	500	–	19.8%
Daly et al. 2020 [36]	United States	COVID-19	T1: 80.2%, T2: 63.9%	Yes	5428–6819	8.9%	T1: 10.5%, T2: 14.2%^c^
Ettman et al. 2020 [37]	United States	COVID-19	64.3%	Yes	1441	8.5%	27.8%^e,f^
Garre-Olmo et al. 2020 [39]	Girona, Spain	COVID-19	81.7%	–	692	–	12.7% (10.3–15.4)
Holingue et al. 2020 [31]	United States	COVID-19	63.0%	Yes	5065	–	9.5%^c^
Ko et al. 2006 [40]	Taiwan	SARS	–	–	1499	–	3.7%
Li et al. 2020 [41]	Hong Kong	COVID-19	71.4%	–	3011	–	21.3% (19.9–22.8)
Twenge et al. 2020 [33]	United States	COVID-19	–	Yes	39,447–119,897	6.6%	T1: 23.5%, T2: 24.1%, T3: 24.4%, T4: 24.9%^d^
Zhao et al. 2020 [35]	Hong Kong	COVID-19	61.3%	Yes	1501	T1: 7.2%, T2: 6.3%	14.8%
*Post-traumatic stress disorder*							
Jalloh et al. 2018 [42]	Sierra Leone	EVD	97.9%	Yes	3564	–	16% (14.7–17.1)
Lau et al. 2005 [43]	Hong Kong	SARS	57.7%	–	818	–	15.7%^e^
*Psychological distress*							
Bruine de Bruin 2020 [29]	United States	COVID-19	79%	Yes	6666	–	11.2%^b,c^
Cénat et al. 2020 [45]	Équateur, Congo	EVD	98.6%	–	1614	–	45.6% (42.0–49.2)
Chandola et al. 2020 [46]	United Kingdom	COVID-19	39.2–49%	Yes	13,754–17,761	–	T1: 37.2%, T2: 34.7%, T3: 32.1%, T4: 25.8%^g^
Daly et al. 2020 [47]	United Kingdom	COVID-19	46.0–48.6%	Yes	14,393	24.3%^h^	T1: 37.8%, T2: 34.7%, T3: 31.9%^g^
Harris et al. 2020 [48]	Norway	COVID-19	–	Yes	4008	–	<1%
Jalloh et al. 2018 [42]	Sierra Leone	EVD	97.9%	Yes	3564	–	6% (5.4–7.0)
Kämpfen et al. 2020 [49]	United States	COVID-19	78.1%	Yes	6585	–	11.2%^c^
Li et al. 2020 [50]	United Kingdom	COVID-19	41.2%	Yes	15,530	–	29.2%
McGinty et al. 2020 [51]	United States	COVID-19	70.4%	Yes	1468	3.9%	13.6% (11.1–16.5)^f^
McGinty et al. 2020 [52]	United States	COVID-19	T1: –, T2: 91.2%	Yes	1337	–	T1: 14.2% (11.3–17.7), T2: 13.0% (10.1–16.5)^f^
Niedzwiedz et al. 2020 [53]	United Kingdom	COVID-19	48.6%	Yes	9748	19.4%^h^	30.6% (29.1–32.3)^g^
Peng et al. 2010 [54]	Taiwan	SARS	68.3%	Yes	1278	–	11.7%
Pierce et al. 2020 [55]	United Kingdom	COVID-19	41.2%	Yes	17,452	18.9%^h^	27.3% (26.3–28.2)^g^
Riehm et al. 2020 [56]	United States	COVID-19	81.6%	Yes	6329	–	11.3%^c^
Robinson et al. 2020 [57]	United States	COVID-19	–	Yes	5146–5784	–	T1: 10.5%, T2: 16.0%, T6: 9.8%^c^
*Alcohol use disordersc*							
Jackson et al. 2020 [58]	England, United Kingdom	COVID-19	–	Yes	1674	25.1%	38.3%

Full table with additional information (i.e. baseline data, phase of epidemic, study design, survey method, age range, measure) and full reference list can be found in the Supplementary Information.

*COVID-19* Coronavirus Disease 2019, *EVD* Ebola Virus Disease, *SARS* severe acute respiratory syndrome; – not reported.

^a^Reported by the authors based on cross-sectional data from another sample or longitudinal data of the same cohort.

^b^Numerical data was obtained by contacting the corresponding author.

^c^Understanding America Study.

^d^Household Pulse Survey.

^e^Same data reported in another study was omitted.

^f^NORC’s AmeriSpeak panel.

^g^UK Household Longitudinal Study.

^h^Longitudinal data of the same cohort.

### Assessment of mental health outcomes

Probable depression was most frequently assessed, followed by probable anxiety, suspected post-traumatic stress disorder (PTSD), and psychological distress (Table 1). We use the term probable as most studies used screening instruments. We used the term suspected PTSD as nearly all studies were conducted during epidemics, and often DSM Criterion A was not assessed or clearly defined. Table 2 lists the studies that assessed prevalence of mental disorders in probability samples during or after epidemics. Table 3 summarises the correlates consistently identified by at least two studies during COVID-19.

**Table 3:** Correlates for adverse mental health in the general population following COVID-19.

*Anxiety*	
Demographic	Female [70, 73,144–159]
	Higher education [153,154]
	Lower income [65,146,160]
	Unemployed/not working [65,155]
	Health personnel [79,147–149,161]
Individual	Pre-existing medical conditions [96,146,148,156,157,162]
	Poorer self-rated health [64, 71,154,163,164]
Exposure to epidemic	Self/family/acquaintances quarantined/infected/died [65, 74,148,149,156,157,161,165]
	Close contact with infected individuals [66, 74, 79]
	Living in high-risk areas [32, 74, 79,161,164,166]
	Exposure to epidemic-related news via:
	Social media [64, 66,163,167], General media [65,168]
	Higher epidemic-related worries/fears [73, 74,157]
	Greater impact on daily life [62,154,162,165]
	Under lockdown or mass stay-at-home orders [67, 68]
	Reduced outside or physical activities [68, 72, 80,154]
	Loneliness [70, 71]
	Adverse economic impacts [62, 98,148,155–157,165]
Perception	Higher perceived susceptibility [32, 62, 70, 74, 98,148,165]
*Depression*	
Demographic	Female [41, 70,147,148,152,155–157,159,169]
	Being widowed/divorced/separated [37, 41, 77,156]
	Lower income [37, 41,160,170]
	Unemployed [41,155,169]
	Living alone [152,155,170]
Individual	Pre-existing medical conditions [78, 96,146,148,155,157,162,171]
	Poorer self-rated health [64,154]
	Prior stressful life events [77, 84]
	Negative coping strategies [72, 76]
Exposure to epidemic	Self/family/acquaintances quarantined/infected/died [65, 74,157]
	Close contact with infected individuals [66,172]
	Exposure to epidemic-related news via:
	Social media [66,173], General media [65, 76]
	Presence of physical symptoms [75, 76]
	Higher epidemic-related worries/fears [39, 73, 74,157,174]
	Greater impact on daily life [62,162,165,172]
	Loneliness [70, 71]
	Home confinement [34, 79, 80]
	Adverse economic impacts [38, 39, 41, 62, 76, 98,151,152,155–157,165]
Perception	Higher perceived susceptibility [62, 70, 74, 75, 98,165]
	Higher perceived severity [75, 76,171]
*Post-traumatic stress disorder*	
Demographic	Female [81–83, 85,156,175–177]
	Younger age [82,156,176,177]
Individual	Pre-existing medical conditions [85,162,176,178]
Exposure to epidemic	Self/family/acquaintances quarantined/infected/died [82, 85,156]
	Exposure to epidemic-related news via: General media [83,176]
	Greater impact on daily life [162,177]
	Adverse economic impacts [156,177]
Perception	Higher perceived susceptibility [82,176–178]
*Psychological distress*	
Demographic	Female [50, 81, 82, 86–88, 92,179–182]
	Younger age [82, 87–89, 91,181,183,184]
	Lower income [89,185]
Individual	Pre-existing medical conditions [81, 88, 89, 91, 92,180,182,183,185]
	Adoption of preventive measures not recommended by WHO (e.g. taking antibiotics, vitamins) [92, 93]
Exposure to epidemic	Self/family/acquaintances quarantined/infected/died [82, 89,181,184]
	Presence of physical symptoms [50, 81, 86]
	Increased exposure to virus [45, 86]
	Higher epidemic-related worries/fears [87,182]
	Exposure to epidemic-related news via: General Media [87, 88]
	Adverse economic impacts [88, 89, 91, 98,180,181]
	Family conflicts [91,183]
Perception	Higher perceived susceptibility [82, 87, 89,181]

Correlates detected in two or more studies and controlled for confounders are listed. Full reference list can be found in the Supporting Information.

### Anxiety

#### Prevalence

The prevalence of probable anxiety ranged from 14.0% to 32.8% in the general population during COVID-19 (Table 2) [29–34]. No eligible studies were identified for past epidemics. The pooled prevalence was 20.7% (95% CI 12.9–29.7), with high heterogeneity (*I*^2^ = 99%; Fig. S1) and major asymmetry indicated by the DOI plot and LFK index of −8.6 (Fig. S2). Anxiety levels appeared to be higher during COVID-19 compared to the reported baselines (Table 2) [33, 35]. In the US, anxiety prevalence has remained high five months into the COVID-19 epidemic, where anxiety was documented to have increased from 8.2% before the epidemic to 29.4% [33].

#### Correlates

During COVID-19, higher risk of probable anxiety was observed in females, those who were unemployed or lived alone (Table 3). Higher perceived susceptibility and severity [32, 62], having multiple COVID-19 risk factors [63], masks shortage [30], frequent traditional and social media exposure [64–66], lockdown or mass stay-at-home orders [67, 68], perceived inadequate housing conditions to cope with lockdowns [69], loneliness [70, 71], reduced outside or physical activities [68, 72], greater impact on daily life [62], and adverse economic impacts [62] were associated with probable anxiety. Higher resilience [73], social support [66], getting reliable, adequate and timely epidemic information [74], perceived effectiveness and adoption of physical distancing and personal preventive measures [32, 34], and having enough basic supplies [74] were associated with a lower risk of probable anxiety.

### Depression

#### Prevalence

The prevalence of probable depression ranged from 9.5% to 27.8% in the general population during COVID-19 (Table 2) [29–31, 33–40, 50]. The pooled prevalence was 18.1% (95% CI 13.0–23.9), with high heterogeneity (*I*^2^ = 99%; Fig. S1) and major asymmetry indicated by the DOI plot and LFK index of −4.42 (Fig. S2). Compared to pre-COVID-19 periods, depression appeared to have increased during COVID-19 in Hong Kong (from 6.3% to 14.8%) and US (from 6.6% to 24.9%) [33, 35]. For past epidemics, probable depression was 3.7% in Taiwan one month after the SARS epidemic [40].

#### Correlates

During COVID-19, females, those who were unemployed or lived alone had a higher risk of probable depression (Table 3). Higher perceived susceptibility and severity [62, 75], COVID-like-symptoms [75, 76], frequent traditional and social media exposure [65, 66], masks shortage [30, 75], unclear mask reuse guidelines [75], disruptions to daily life [62], financial stressors and uncertainties [38, 62], marital conflict [77], experiences of physical and psychological abuse [78], home confinement [34, 79, 80], perceived inadequate housing conditions to cope with lockdowns [69], and loneliness [71] were associated with probable depression. Higher resilience [73], social support [66, 78], increased physical activity [69, 72], accurate and timely epidemic information [74], promotion of preventive measures by government [75], perceived effectiveness and adoption of physical distancing and personal preventive measures [34, 74] and sufficient basic supplies [74] were protective against probable depression.

### Post-traumatic stress disorder

#### Prevalence

The prevalence of suspected PTSD for COVID-19 has not been examined in a random sample. Among non-probability samples, the prevalence ranged from 1.7% to 100% indicating the importance of using population-representative samples (Table S8). For Ebola virus disease and SARS, the pooled prevalence of suspected PTSD was 16.0% (95% CI 14.9–17.1), with low heterogeneity between studies (*I*^2^ = 0%; Fig. S1) [42–44].

#### Correlates

The threat of death during COVID-19 (e.g. having COVID-19-like symptoms [81], being unsure if oneself had contracted the virus or had close contact with infected people [82], knowing someone who were infected or died from COVID-19 [82]) and media exposure to COVID-19 news [83] were associated with suspected PTSD. Individuals with lower resilience and stressful life events had elevated risk of suspected PTSD [84, 85].

### Psychological distress

#### Prevalence

The prevalence of psychological distress ranged from <1% to 37.8% in the general population during COVID-19 (Table 2) [29, 46–53, 55–57]. Our meta-analysis included the very low estimate in Norway (<1% vs ≥11.2% in other studies) [48], though it might be an outlier. The pooled prevalence was 13.0% (95% CI 0–34.1), with high heterogeneity (*I*^2^ = 100%; Fig. S1) yet minor asymmetry indicated by the DOI plot and LFK index of −1.62 (Fig. S2). Compared with pre-COVID-19 periods, psychological distress increased during COVID-19 in UK (from 18.9% to 27.3%) and US (3.9% to 13.6%) [51, 55]. However, separate studies have detected a stagnating or even declining trend in psychological distress in the US (from 14.2% to 13.0%) and UK (from 37.2% to 25.8%) from April to July 2020 [46, 52]. Studies on past epidemics indicated potential enduring mental health impact of novel epidemics. For example, the prevalence of psychological distress remained at around 6% and 45.6% respectively in Sierra Leone and Équateur of Congo towards and after the end of the Ebola virus disease epidemic [42, 45]. A study in Taiwan also reported 11.7% of adults having psychological distress four months after SARS [54].

#### Correlates

During COVID-19, females and those who were younger and had lower income showed a higher risk of psychological distress (Table 3). COVID-19-like symptoms [50, 86], worries of self-infection [82], increased risk of exposure to virus [45, 86], media exposure [87, 88], income loss [88, 89], other disruptions of daily life [90], family conflicts [91], and adoptions of preventive measures not recommended by WHO (e.g. taking antibiotics [92, 93]) were associated with psychological distress. Trust in the government and health system [81, 87], perceived effectiveness of preventive measures [87], and adoption of physical distancing [87] were associated with less psychological distress.

### Other mental disorders

During COVID-19, the prevalence of alcohol use disorders increased from 25.1% before the lockdown to 38.3% during lockdown in England (Table 2) [58]. No prevalence data for acute stress disorder and obsessive-compulsive disorder were available from probability samples. Correlates for acute stress disorder during COVID-19 included younger age, lower income, pre-existing health conditions, self or family or friends being infected or quarantined, and increased exposure to virus (e.g., frontline workers; those living in high-risk areas) [94]. Higher resilience was associated with less obsessive-compulsive disorder [95].

### Suicidality

None of the identified studies examined suicidal ideation in probability samples during or after an epidemic, although national registers showed that suicide rates in Norway and Queensland, Australia remained largely unchanged during COVID-19 [59, 60]. In non-probability samples, the prevalence of suicidal ideation during COVID-19 ranged from 2.8% to 14.2% (Table S8).

#### Correlates

Younger age [96], lower socioeconomic status [96], pre-existing mental health conditions [96], insomnia [97], serious marital conflicts [77], stress due to the pandemic [97], and COVID-19 health-related and economic worries [98] were associated with suicidal ideation during COVID-19.

### Evidence appraisal

Study quality ranged from low to high (total NOS score 0–7 out of 9), with 2% classified as high quality (*n* = 6) and 70% (*n* = 179) as medium quality (Table S9). Yet, the quality of these studies might have been underestimated, as all studies had scored 0 for “ascertainment of exposure” and “selection of non-exposed sample” due to the lack of standardised, validated measures of exposure and non-exposed samples. Certainty of evidence based on the GRADE rating was low for anxiety, depression, psychological distress, and suicidality, and very low for PTSD and other mental outcomes. Major issues included lack of longitudinal data, high heterogeneity across studies, convenience sampling, paucity of diagnostic interviews, and potential publication bias. Nonetheless, the prevalence studies in general provided evidence for sample representativeness. These studies adopted probability-based sampling with weighting strategies to account for response bias, attribution bias, and differences with the underlying population (Table 2). The reported median response rate was 63.8% (Table 2), which is quite high given the context of an ongoing pandemic and overall declining trends in participation rates in epidemiologic studies [99].

## Discussion

To date, this is the most comprehensive systematic review of novel epidemics and population mental health. Our meta-analysis showed that COVID-19 and other epidemics of a smaller scale were all associated with a substantial population mental health burden (Fig. S1). In randomly sampled populations, one in five adults had a probable mental disorder during COVID-19 (Fig. S1). This would be comparable to the levels observed in previous epidemics (i.e. Ebola virus disease), major disasters and armed conflicts [100–102], though mental health response to COVID-19 pandemic may vary greatly across settings. Where pre-pandemic prevalence was reported, our identified studies generally indicated an increase in anxiety, depression, psychological distress, and alcohol use disorders during COVID-19 (Table 2). This is consistent with the recent estimation that the global burden of anxiety and depression had increased during the COVID-19 pandemic [103]. We summarised correlates for poor mental health during COVID-19 (Table 3), which is crucial to identifying vulnerable groups when mental health responses are highly heterogeneous [104, 105]. Perceived risk of infection [62], exhibiting COVID-19-like symptoms [75, 76], masks shortage [30, 75], and unclear mask reuse guidelines [75] were associated with anxiety and depression. Providing accurate information and timely tests may therefore allay anxiety [74, 106]. Notably, lockdowns and home confinement during COVID-19 may also have exacerbated mental health conditions [34, 67, 68, 79, 80], particularly among those who had marital or family conflicts [77, 91], experiences of physical and psychological abuse [78], and inadequate housing conditions to cope with lockdowns [69]. While the scale of lockdowns and the infodemic during COVID-19 are unprecedented [107, 108], the psychological toll of lockdowns could be mitigated by social support, acting as a buffer for stressful environments [66, 109]. Social media appears to have become more influential on mental health than traditional media during recent major population events [66, 110, 111]. Indeed, heavy COVID-19-related social media use was associated with anxiety, depression and acute stress, possibly due to the spread of the “emotional contagion”, conflicting COVID-19 information, and fear-inducing misinformation via online social networks [64, 66, 112, 113]. These findings support the WHO’s recommendations on enhancing social support during the pandemic and reducing time spent on distressing COVID-19-related news [114].

Policy makers and service providers need to know who is the most vulnerable to guide priority setting and interventions [10, 18]. While all age groups have experienced poorer mental health during COVID-19 [55], the young can be disproportionately affected by countermeasures [55, 115]. Young adults, women, and those living with young children were found to be at higher risk for poor mental health during the lockdown in the UK [55]. In the US, young adults had the highest prevalence of anxiety or depressive disorders, COVID-19-related trauma- and stressor-related disorders, initiation of or increased substance use, and serious suicidal ideation in the previous month [115]. Decreased time for learning and living conditions during lockdown also had a clear impact on mental health of students [116]. Other vulnerable groups include survivors, health care workers, ethnic minorities, essential workers, unpaid caregivers for adults, those with low income and job loss, and people with pre-existing medical conditions including mental health disorders [37, 91, 96, 115, 117, 118]. In contrast, better mental health during COVID-19 was hypothesised for those with high levels of socioeconomic security due to the ability to work from home and having more time with family, but this has not been established [55]. Nevertheless, individuals and populations often exhibit remarkable resilience following major emergencies, with the majority not developing psychopathology [22, 119]. This lends support for targeted interventions following epidemics rather than mass interventions [120]. The stepped care model has been recommended during COVID-19 where the most effective, least resource-intensive treatments are provided to patients first, and more resource-heavy interventions then stepped up according to patients’ needs [121]. Digital psychological interventions have shown promise in LMICs [122], however, the epidemic and interventions could also widen entrenched patterns of inequities across settings [123]. Nurse-led approaches within a non-specialist setting could help deliver mental health and psychosocial support services to individuals when psychiatric hospitals are closed during epidemics [124]. Upstream approaches targeting the population determinants of health could address inequities by preserving the economy, reducing job loss, and implementing social policies to prevent substance use and domestic violence [125–127].

The COVID-19 pandemic represents a three-way tug-of-war between COVID-19 suppression, economic preservation, and population well-being [128]. An uncontrolled epidemic, prolonged stringent interventions such as lockdowns, and economic recession could all profoundly affect population mental health. In contrast, addressing the emerging and prevailing determinants of mental health would mitigate the psychological toll of the pandemic. This in turn could reduce pandemic fatigue, promote social acceptance and adherence to interventions [128–130]. Protecting population mental health has therefore become even more important during COVID-19 [131]. Yet the psychobehavioural responses to epidemics depend on a interplay between threat perception, stress and coping, individual and collective interests, social context, leadership, and risk communication [130, 132]. Maintaining public trust in authorities and incorporating altruism in health messaging could improve mental well-being and adherence to interventions [11, 18, 130, 133, 134]. Indeed, adoption of physical distancing and personal preventive measures in general have been associated with lower risk of anxiety, depression and psychological distress.

Nevertheless, our review did not include studies on mental health symptoms and transdiagnostic outcomes, thereby limiting the assessment of mental health impact of COVID-19 and other epidemics. In particular, insomnia is a very prevalent mental health condition during epidemics, with an estimation of one in three adults reporting insomnia during COVID-19 [4]. Furthermore, we have identified several major limitations of the extant literature. First, as with other reviews on COVID-19 and previous epidemics [3–9, 12, 13, 102], there was considerable heterogeneity across studies, possibly due to differences in study design and the magnitude of epidemics, countermeasures, and consequences (e.g. social and economic costs) [10, 135]. However, this limitation is inherent to psychiatric epidemiological research following major emergencies [101], and was partly addressed by including more comparable random samples for our meta-analysis. Second, more random samples are needed to provide reliable estimates of the mental health burden of epidemics and to allow a meta-regression to explore reasons for the observed heterogeneity [10]. For instance, the pooled prevalence of psychological distress was lower than specific mental disorders due to the inclusion of a study in Norway which reported a very low prevalence estimate (<1%) [48]. Prior meta-analyses that have relied on convenience samples or opt-in online panels would have included more studies but may have overestimated the population mental health burden associated with COVID-19 [3–6, 8, 9]. The high proportion of probability samples during past epidemics (87.5%) compared to COVID-19 (~10%) suggests that probability samples are possible during epidemics (e.g. via random-digit-dialling, address-based sampling) and should be used to generate high-quality evidence during COVID-19 [10]. Third, longitudinal, population-representative cohorts with baseline data are needed. In this review, we were only able to identify one such cohort (i.e. UK Household Longitudinal Study) [47, 53, 55]. All other studies with pre-pandemic baseline data were cross-sectional and compared different samples. Due to the inherent differences among individuals, it is difficult to discern the changes in prevalence attributable to the COVID-19 pandemic. Baseline data is particularly important when unexpected values of prevalence estimates (e.g. <1% of psychological distress in Norway [48]) were found. Also, psychological distress remained high after the Ebola virus disease epidemic [42, 45], indicating that ongoing surveillance of population mental health and long-term studies are needed for COVID-19 even when we have exited the pandemic. Future research should prioritise longitudinal, population-representative samples with pre-epidemic data and long-term outcomes [10, 18]. This may be difficult but have been successfully implemented by nesting follow-ups in existing random samples where available (e.g. UK Household Longitudinal Study, Hong Kong FAMILY Cohort) [66, 110]. Such cohorts could be instrumental to informing the appropriate response and mobilisation of resources and mental health services [10, 55, 110, 136]. Fourth, all random samples during COVID-19 were conducted in high-income settings. Language restrictions of our search may have excluded studies published in other languages. Resources and studies in low and middle-income countries where COVID-19 has a large impact are needed, and the health system and economy of individual countries could be particularly vulnerable to the consequences of the pandemic [137]. Fifth, most studies relied on screening instruments, and the findings could represent acute reactions to a stressful event as opposed to true psychopathology [138]. However, the screening instruments have been shown to be valid and reliable (Table S3) and we excluded studies using non-validated mental health measures (e.g. self-conceived questions, single-item measures) [10]. Lastly, to date, the prevalence of PTSD or obsessive-compulsive disorder during COVID-19 has not been examined using a random sample. It is well-known that PTSD is highly prevalent following population shocks [139]. By including findings from SARS and Ebola virus disease in our review, we estimated that the prevalence of suspected PTSD may approximate 16% during epidemics. Yet, the applicability of these findings to COVID-19 is unclear.

## Conclusion

Our study shows that the psychological toll of COVID-19 and past epidemics was substantial and widespread in the community. Novel infectious diseases can therefore spill over from infected individuals to the community-at-large, where even those not directly exposed to the pathogen experience psychiatric sequelae. Health-care professionals need to be vigilant in recognising mental health sequelae in the general population. However, the resources available for prevention and treatment of mental disorders in most countries have diminished given competing demands [131]. This needs to be urgently redressed as mental health is uniquely placed to improve the whole spectrum of well-being, and thus should be at the forefront of the health agenda [129, 140]. With further waves of COVID-19 anticipated and the inevitability of new epidemics [141, 142], ongoing surveillance of the mental health impact of epidemics and public mental health interventions to build community resilience should be integrated into preparedness plans worldwide [143].

## Supplementary information

Supplementary information

PRISMA Flowchart

PRISMA 2020 Checklist
