# Supplementary material for: Mental disorders following COVID-19 and other epidemics: a systematic review and meta-analysis
Source: Transl Psychiatry. 2022 May 17;12:205. doi: 10.1038/s41398-022-01946-6 (PMC9110635; doi:10.1038/s41398-022-01946-6)
Supplement: Supplementary file 1 — Supplementary information [file 41398_2022_1946_MOESM1_ESM.pdf]

Supplementary Information

Fig. S1. Forest plots of pooled prevalence of (A) probable anxiety, (B) probable depression, (C) suspected post-traumatic stress disorder, and (D) psychological distress in the general population. All forest plots are adjusted for study quality……2

Fig. S2. Doi plots of pooled prevalence of (A) probable anxiety, (B) probable depression, and (C) psychological distress in the general population……3

Table S1. Detailed search strategy……4

Table S2. Selection criteria of studies……6

Table S3. Summary of validated, standardised mental health measures……7

Table S4. Adapted Newcastle-Ottawa Scale……9

Table S5. Raw data of probability samples included in meta-analysis……12

Table S6. List of countries with published studies on novel epidemics and mental health……16

Table S7. Prevalence of mental health outcomes during and after novel epidemics in probability or complete samples of general population……17

Table S8. Prevalence of mental health outcomes during and after novel epidemics in non-probability samples of general population……20

Table S9. Study quality ratings based on adapted Newcastle-Ottawa Scale……32

Full list of references……45

Fig. S1. Forest plots of pooled prevalence of (A) probable anxiety, (B) probable depression, (C) suspected post-traumatic stress disorder, and (D) psychological distress in the general population. All forest plots are adjusted for study quality.

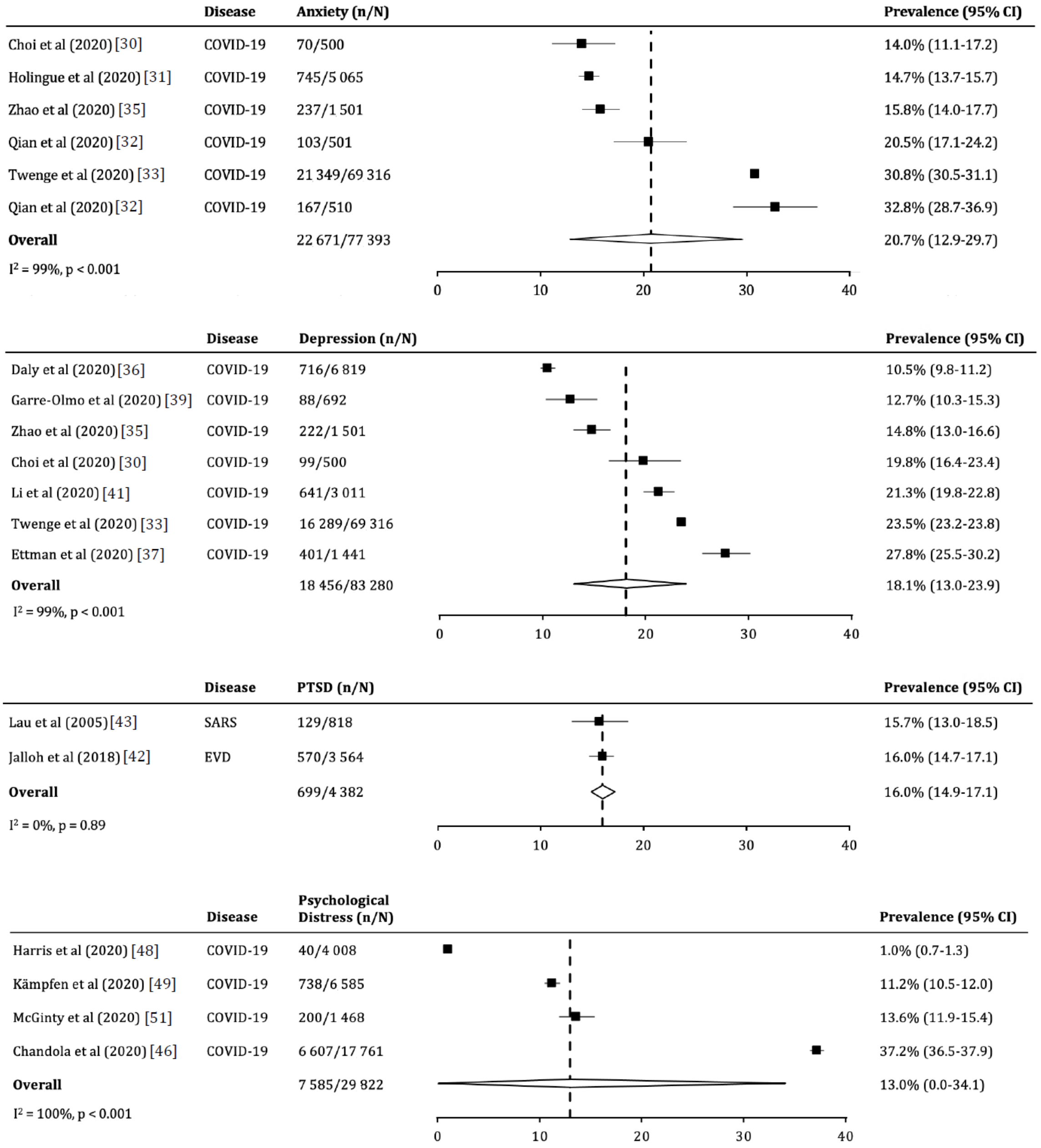

COVID-19=Coronavirus Disease 2019. EVD=Ebola virus disease. PTSD=post-traumatic stress disorder. SARS=severe acute respiratory syndrome.

Fig. S2. Doi plots of pooled prevalence of (A) probable anxiety, (B) probable depression, and (C) psychological distress in the general population

A.

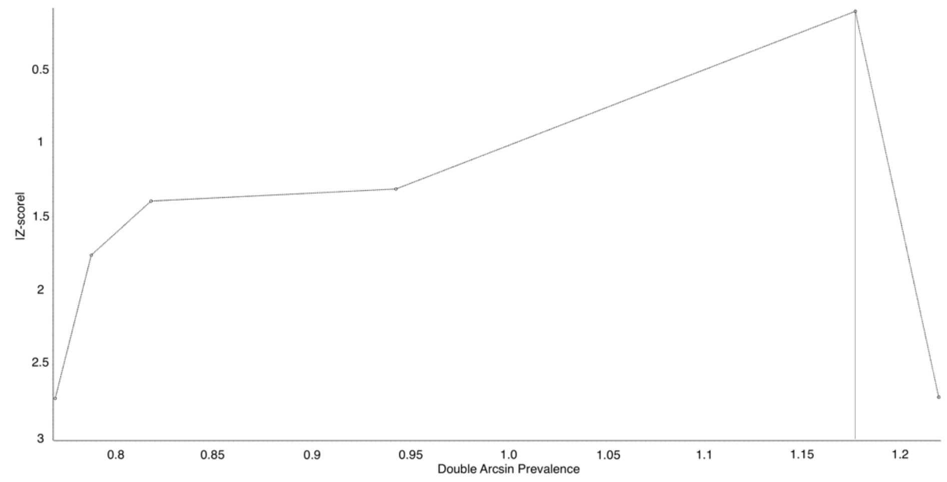

B.

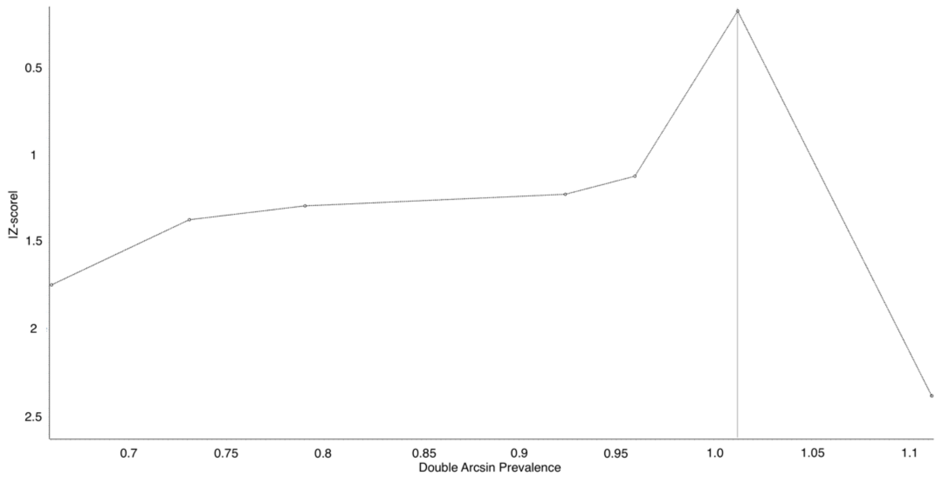

C.

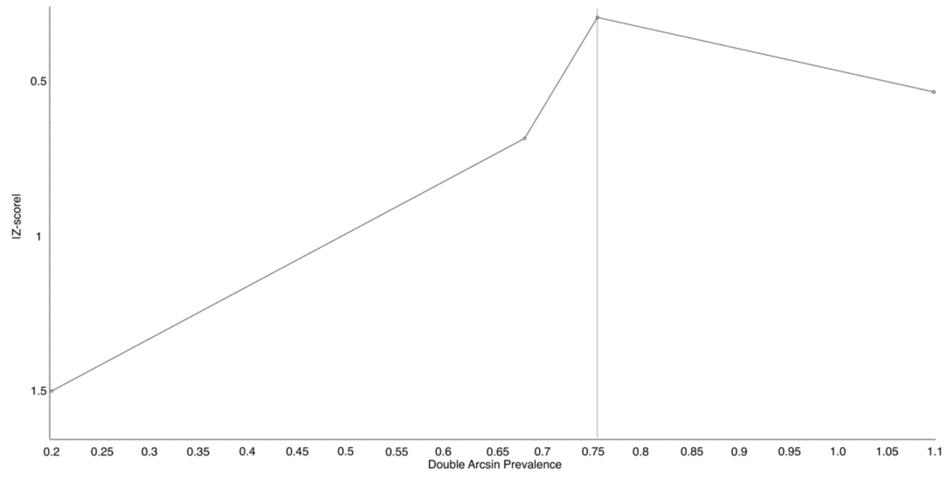

Note: At least two studies are required to generate Doi plots.

Table S1. Detailed search strategy

We used search terms related to any novel outbreaks that were droplet- or airborne-transmissible, including general terms such as outbreak, epidemic, pandemic, influenza, avian flu, and more specific terms such as COVID-19, EVD, MERS, avian influenza A(H7N9), pandemic influenza A(H1N1), avian influenza A(H5N1), and SARS. The search strategy also included any terms related to mental disorders. MeSH terms and explosions of terms were applied where appropriate. Such search strategies casted a broader net to minimise the chance of missing key studies.

Database	Search string
CINAHL Plus	(TX ("coronavirus" OR "COVID" OR "COVID-19" OR "SARS-CoV-2" OR "2019-nCoV" OR "severe acute respiratory syndrome" OR "SARS" OR "Middle East respiratory syndrome" OR "MERS" OR "ebola" OR "swine flu" OR "H1N1" OR "avian flu" OR "H5N1" OR "H7N9" OR "influenza") OR MH (Disease Outbreak OR outbreak* OR epidemic* OR pandemic*)) AND (TX ("mental health" OR "mental disorders" OR "mental illness" OR "depression" OR "depressive symptoms" OR "anxiety" OR "PTSD" OR "PTSS" OR "posttraumatic stress symptoms" OR "acute stress disorder" OR "acute stress symptoms" OR "suicide" OR "suicidality" OR "suicidal" OR "deliberate self-harm" OR "psychological wellbeing" OR "psychological well-being" OR "mental wellbeing" OR "mental well-being" OR "distress" OR "psychological" OR "psychiatric diagnoses" OR "alcohol" OR "substance use") OR MH (mental health OR mental disorder OR mental illness OR mood disorder OR depression OR anxiety disorder OR trauma and stressor related disorder OR PTSD OR neurotic disorder OR suicide OR stress, psychological OR behavioural symptom OR schizophrenia OR psychotic disorder))
Embase	(((coronavirus or COVID or COVID-19 or SARS-CoV-2 or 2019-nCoV or severe acute respiratory syndrome or SARS or Middle East respiratory syndrome or MERS or ebola or swine flu or H1N1 or avian flu or H5N1 or H7N9 or influenza).mp. [mp=title, abstract, heading word, drug trade name, original title, device manufacturer, drug manufacturer, device trade name, keyword, floating subheading word, candidate term word]) or (*Disease Outbreak/ or *outbreak*/ or *epidemic*/ or *pandemic*/)) AND (("mental health" or "mental disorders" or "mental illness" or "depression" or "depressive symptoms" or "anxiety" or "PTSD" or "PTSS" or "posttraumatic stress symptoms" or "acute stress disorder" or "acute stress symptoms" or "suicide" or "suicidality" or "suicidal" or "deliberate self-harm" or "psychological wellbeing" or "psychological well-being" or "mental wellbeing" or "mental well-being" or "distress" or "psychological" or "psychiatric diagnoses" or "alcohol" or "substance use").mp. [mp=title, abstract, heading word, drug trade name, original title, device manufacturer, drug manufacturer, device trade name, keyword, floating subheading word, candidate term word] or ((*mental health/ or *mental disorder/ or *mental illness/ or *mood disorder/ or *depression/ or *anxiety disorder/ or *trauma/) and stressor related disorder/) or *PTSD/ or *neurotic disorder/ or *suicide/ or *stress, psychological/ or *behavioural symptom/ or *schizophrenia/ or *psychotic disorder/))

Database	Search string
PsycINFO	(coronavirus OR COVID OR COVID-19 OR SARS-CoV-2 OR 2019-nCoV OR severe acute respiratory syndrome OR SARS OR Middle East respiratory syndrome OR MERS OR ebola OR swine flu OR H1N1 OR avian flu OR H5N1 OR H7N9 OR Disease Outbreak OR outbreak* OR epidemic* OR pandemic*) AND (mental health OR mental disorder OR mental disorders OR mental illness OR mood disorder OR depression OR depressive symptoms OR anxiety disorder OR anxiety OR trauma and stressor related disorder OR PTSD OR PTSS OR posttraumatic stress symptoms OR acute stress disorder OR acute stress symptoms OR neurotic disorder OR suicide OR suicide OR suicidality OR suicidal OR deliberate self-harm OR psychological wellbeing OR psychological well-being OR mental wellbeing OR mental well-being OR distress OR stress, psychological OR behavioural symptom OR psychological OR psychiatric diagnoses OR alcohol OR substance use OR schizophrenia OR psychotic disorder)
PubMed	(coronavirus [All fields] OR COVID [All fields] OR COVID-19 [All fields] OR SARS-CoV-2 [All fields] OR 2019-nCoV [All fields] OR severe acute respiratory syndrome [All fields] OR SARS [All fields] OR Middle East respiratory syndrome [All fields] OR MERS [All fields] OR ebola [All fields] OR swine flu [All fields] OR H1N1 [All fields] OR avian flu [All fields] OR H5N1 [All fields] OR H7N9 [All fields] OR influenza [All fields] OR Disease Outbreak [MeSH] OR outbreak* [All fields] OR epidemic [MeSH] OR epidemic* [All fields] OR pandemic [MeSH] OR pandemic* [All fields]) AND (mental health [MeSH] OR “mental health” [All fields] OR mental disorder [MeSH] OR “mental disorders” [All fields] OR mental illness [MeSH] OR “mental illness” [All fields] OR mood disorder [MeSH] OR depression [MeSH] OR “depression” [All fields] OR “depressive symptoms” [All fields] OR anxiety disorder [MeSH] OR “anxiety” [All fields] OR trauma and stressor related disorder [MeSH] OR PTSD [MeSH] OR “PTSD” [All fields] OR “PTSS” [All fields] OR “posttraumatic stress symptoms” [All fields] OR “acute stress disorder” [All fields] OR “acute stress symptoms” [All fields] OR neurotic disorder [MeSH] OR suicide [MeSH] OR “suicide” [All fields] OR “suicidality” [All fields] OR “suicidal” [All fields] OR “deliberate self-harm” [All fields] OR “psychological wellbeing” [All fields] OR “psychological well-being” [All fields] OR “mental wellbeing” [All fields] OR “mental well-being” [All fields] OR “distress” [All fields] OR stress, psychological [MeSH] OR behavioural symptom [MeSH] OR “psychological” [All fields] OR “psychiatric diagnoses” [All fields] OR “alcohol” [All fields] OR “substance use” [All fields] OR schizophrenia [MeSH] OR psychotic disorder [MeSH])
Web of Science	((ALL=("coronavirus" OR "COVID" OR "COVID-19" OR "SARS-CoV-2" OR "2019-nCoV" OR "severe acute respiratory syndrome" OR "SARS" OR "Middle East respiratory syndrome" OR "MERS" OR "ebola" OR "swine flu" OR "H1N1" OR "avian flu" OR "H5N1" OR "H7N9" OR "influenza")) or (TS=(Disease Outbreak OR outbreak* OR epidemic* OR pandemic*))) AND ((TS=(mental health OR mental disorder OR mental illness OR mood disorder OR depression OR anxiety disorder OR trauma and stressor related disorder OR PTSD OR neurotic disorder OR suicide OR stress, psychological OR behavioural symptom OR schizophrenia OR psychotic disorder)) OR (ALL=("mental health" OR "mental disorders" OR "mental illness" OR "depression" OR "depressive symptoms" OR "anxiety" OR "PTSD" OR "PTSS" OR "posttraumatic stress symptoms" OR "acute stress disorder" OR "acute stress symptoms" OR "suicide" OR "suicidality" OR "suicidal" OR "deliberate self-harm" OR "psychological wellbeing" OR "psychological well-being" OR "mental wellbeing" OR "mental well-being" OR "distress" OR "psychological" OR "psychiatric diagnoses" OR "alcohol" OR "substance use")))

Table S2. Selection criteria of studies

Inclusion criteria

1) sampled a community exposed to the threat of novel epidemics
2) examined general population
3) reported prevalence or risk factors for mental disorders (assessed with clinical interviews or validated, standardised instruments that could provide a provisional diagnosis based on DSM or ICD) or suicidality (assessed with validated measures)
4) original research studies in peer-reviewed journals
5) published in English, Chinese, and Portuguese

Exclusion criteria

1) only examined emotional responses or other transdiagnostic outcomes (e.g. fear, insomnia)
2) conference abstracts
3) qualitative studies
4) modelling studies
5) examined specific subgroups (e.g. patients, quarantined individuals, and health-care professionals) which would have very different levels of exposure compared to the general population, and have been covered in prior reviews)
6) examined population subgroups (e.g. college students, elderly, pregnant women, people with specific occupations)

Table S3. Summary of validated, standardised mental health measures

Definitions of mental disorders

We use the term probable as all except one study used screening instruments as opposed to diagnostic interviews. We used the term suspected PTSD as nearly all studies were conducted while the epidemic was ongoing, whereas PTSD is often assessed after single, well-defined events (e.g. terrorist attacks, natural disasters, physical or sexual abuse). Further, medical conditions such as life-threatening infections or being quarantined do not meet the current criteria for trauma for PTSD diagnosis[186, 187]. Nevertheless, PTSD has been reported for COVID-19 and other epidemics[11-13], and the term suspected PTSD would be consistent with the World Health Organization's (WHO) likelihood of diagnosis (lowest to highest) ranging from suspected to probable to confirmed[188]. Below lists the measures used by the identified studies.

Probable anxiety

Probable anxiety was assessed using validated screening instruments that could provide a provisional diagnosis, including Beck Anxiety Inventory (BAI)[189], Generalized Anxiety Disorder-2 Scale (GAD-2)[190], Generalized Anxiety Disorder-7 Scale (GAD-7)[191], Hamilton Anxiety Rating Scale (HAM-A)[192], Hospital Anxiety and Depression Scale (HADS)[193], Mini-International Neuropsychiatric Interview (M.I.N.I.)[194], PROMIS Emotional Distress, Anxiety, Short Form[195], Zung's Self-rating Anxiety Scale (SAS)[196]. All instruments and their cut-offs were validated against diagnostic interviews and had high sensitivity and specificity for probable anxiety.

Probable depression

Probable depression was assessed using validated screening instruments that could provide a provisional diagnosis, including Beck Depression Inventory (BDI)[197], Beck Depression Inventory (BDI-II)[198], Centre for Epidemiological Studies-Depression Scale (CES-D)[199], Five-item World Health Organization-Well-Being Index (WHO-5)[200], Hospital Anxiety and Depression Scale (HADS)[193], Mini-International Neuropsychiatric Interview (M.I.N.I.)[194], Patient Health Questionnaire-2 (PHQ-2)[201], Patient Health Questionnaire-8 (PHQ-8)[202], Patient Health Questionnaire-9 (PHQ-9)[203], Taiwanese Depression Questionnaire (TDQ)[204], Zung's Self-rating Depression Scale (SDS)[205]. All instruments and their cut-offs were validated against diagnostic interviews and had high sensitivity and specificity for probable depression.

Suspected post-traumatic stress disorder (PTSD)

Suspected PTSD was assessed using validated screening instruments that could provide a provisional diagnosis, including International Trauma Questionnaire (ITQ)[206], Mini-International Neuropsychiatric Interview (M.I.N.I.)[194], Primary Care Post-Traumatic Stress Disorder Screen for DSM-5 (PC-PTSD-5)[207], variations of Impact of Event Scale-Revised (IES-R)[208], Post-traumatic Stress Disorder Checklist-Civilian Version (PCL-C)[209], and Posttraumatic Stress Disorder Checklist for DSM-5 (PCL-5)[210]. In some studies, the DSM diagnostic algorithm was used by the authors to indicate suspected PTSD. All instruments and their cut-offs were validated against diagnostic interviews and had high sensitivity and specificity for PTSD.

Psychological distress

Psychological distress was assessed using validated screening instruments, including Brief Symptom Inventory-18 (BSI-18)[211], Brief Symptom Inventory-53 (BSI-53)[212], COVID-19 Peri-traumatic Distress Index (CPDI)[213], Five-item Brief Symptom Rating Scale (BSRS-5)[214], General Health Questionnaire-12 (GHQ-12)[215], General Health Questionnaire-28 (GHQ-28)[216], Hopkins Symptoms Checklist-10 (HSCL-10)[217], Kessler Psychological Distress Scale-6 (K6)[218], Kessler Psychological Distress Scale-10 (K10)[218], Mini-International Neuropsychiatric Interview (M.I.N.I.)[194], Patient Health Questionnaire-4 (PHQ-4)[219], Symptom Checklist-90 (SCL-90)[220], Self-reporting Questionnaire-20 (SRQ-20)[221]. All instruments and their cut-offs were validated against diagnostic interviews and had high sensitivity and specificity for psychological distress.

Other mental disorders

Acute stress disorder was assessed using Acute Stress Disorder Scale (ASDS)[222]. Alcohol use disorders were assessed using Alcohol Use Disorder Identification Test (AUDIT)[223], Alcohol Use Disorder Identification Test-Concise (AUDIT-C)[223], and Mini-International Neuropsychiatric Interview (M.I.N.I.)[194]. Agoraphobia was assessed using Mini-International Neuropsychiatric Interview (M.I.N.I.)[194]. Obsessive-compulsive disorder was assessed using Obsessive Compulsive Inventory-Revised (OCI-R)[224]. Panic disorder and social phobia were assessed by Mini-International Neuropsychiatric Interview (M.I.N.I.)[194]. All instruments and their cut-offs were validated against diagnostic interviews and had high sensitivity and specificity for the specific disorders.

Suicidality

Suicide rates were estimated using official data from national registers. Suicidal ideation and suicide attempts were assessed using Centre for Epidemiological Studies-Depression Scale (CES-D)[225], Depressive Symptom Inventory Suicidality Subscale (DSI-SS)[226], Mini-International Neuropsychiatric Interview (M.I.N.I.)[194], Suicide Behaviors Questionnaire-Revised (SBQ-R)[227], Suicidal Ideation Attributes Scale (SIDAS)[228], and Self-Injurious Thoughts and Behaviors Interview (SITBI)[229]. All instruments and their cut-offs were validated for assessing suicidal ideation.

Table S4. Adapted Newcastle-Ottawa scale

Total score: 0-9 (Low 0-3, Medium 4-6, High 7-9)[12, 20]

Cross-sectional studies (*Patra et al 2015*[20])

1) Representativeness of the exposed sample
    - 1 mark for truly representative of the average in the community (all subjects or random sampling)
    - 1 mark for somewhat representative of the average in the community (non-random sampling)
    - 0 mark for selected group of users
    - 0 mark if no description of sampling strategy
2) Selection of the nonexposed sample (*Anglin et al 2013*[21])
    - 1 mark if drawn from the same community as the exposed cohort
    - 0 mark if drawn from a different source
    - 0 mark if no description of the derivation of the non-exposed cohort
3) Ascertainment of exposure (*revised*)
    - 1 mark for validated measurement tool for an exposure
    - 0 mark for non-validated measurement tool, but the tool is available or described
    - 0 mark if no description of the measurement tool
4) Comparability (controlled for age)
    - 1 mark for yes
    - 0 mark for no
5) Comparability (controlled for additional important factors, e.g., sex, education, occupation, income, marital status, baseline health or medical history (mental or physical)/pre-existing illness, or other appropriate confounders given the exposure and outcome)
    - 1 mark for yes
    - 0 mark for no
6) Assessment of outcome (*Rogers et al 2020*[12]; *Ni et al 2020*[22])
    - 1 mark for independent blind assessment
    - 1 mark for record linkage
    - 1 mark for clinical diagnosis using ICD/DSM or a validated measure with a clinical cutoff indicating any psychiatric diagnosis
    - 0 mark for measures without clinical cutoff or newly conceived measures
    - 0 mark if no description

Cohort/ longitudinal studies (*Patra et al 2015*[20])

1) Representativeness of the exposed cohort
    - 1 mark for truly representative of the average in the community (all subjects or random sampling)
    - 1 mark for somewhat representative of the average in the community (non-random sampling)
    - 0 mark for selected group of users
    - 0 mark if no description of sampling strategy
2) Selection of the nonexposed cohort (*Ni et al 2020*[22])
    - 1 mark if drawn from the same community as the exposed cohort
    - 0 mark if drawn from a different source
    - 0 mark if no description of the derivation of the non-exposed cohort

3) Ascertainment of exposure (*Ni et al 2020*[22])
    - 1 mark for secure record (e.g., surgical records)
    - 1 mark for structured interview (e.g each interview is presented with exactly the same questions in the same order and/or validated measurement tool)
    - 0 mark for written self report
    - 0 mark if no description
4) Outcome of interest not present at the start of the study
    - 1 mark for yes
    - 0 mark for no
5) Comparability (controlled for age)
    - 1 mark for yes
    - 0 mark for no
6) Comparability (controlled for additional important factors, e.g., sex, education, occupation, income, marital status, baseline health or medical history (mental or physical)/pre-existing illness, or other appropriate confounders given the exposure and outcome)
    - 1 mark for yes
    - 0 mark for no
7) Assessment of outcome (*revised*)
    - 1 mark for independent blind assessment
    - 1 mark for record linkage
    - 1 mark for clinical diagnosis using ICD/DSM or a validated measure with a clinical cutoff indicating any psychiatric diagnosis
    - 0 mark for measures without clinical cutoff or newly conceived measures
    - 0 mark if no description
8) Lengths of follow-up (*Ni et al 2020*[22])
    - 1 mark for yes (follow-up long enough for outcomes to occur e.g., 2 weeks)
    - 0 mark for no
9) Adequacy of follow-up
    - 1 mark for complete follow up - all subjects accounted for
    - 1 mark if subjects lost to follow up unlikely to introduce bias - small number lost (i.e., >=70% follow up), or description provided of those lost, or evidence that no significant differences between subjects retained and subjects lost
    - 0 mark if follow up rate <70% and no description of those lost
    - 0 mark for no statement

Case-control studies (*Patra et al 2015*[20])

1) Is the case definition adequate?
    - 1 mark for yes, with independent validation
    - 0 mark for yes, e.g., record linkage or based on self-reports
    - 0 mark if no description
2) Representativeness of the cases
    - 1 mark for consecutive or obviously representative series of cases
    - 0 mark for potential for selection biases or not stated
3) Selection of controls
    - 1 mark for community controls
    - 0 mark for hospital controls
    - 0 mark if no description
4) Definition controls
    - 1 mark for no history of exposure
    - 0 mark if no description of source
5) Comparability (controlled for age)
    - 1 mark for yes
    - 0 mark for no

6) Comparability (controlled for additional factors, e.g., sex, education, occupation, income, marital status, baseline health or medical history (mental or physical)/pre-existing illness, or other appropriate confounders given the exposure and outcome)
    - 1 mark for yes
    - 0 mark for no
7) Ascertainment of exposure
    - 1 mark for secure record (e.g., surgical records)
    - 1 mark for structured interview where blind to case/control status (e.g each interview is presented with exactly the same questions in the same order and/or validated measurement tool)
    - 0 mark for interview not blinded to case/control status
    - 0 mark for written self-report or medical record only
    - 0 mark if no description
8) Same method of ascertainment for cases and controls
    - 1 mark for yes
    - 0 mark for no
9) Non-response rate
    - 1 mark if same rate for both groups
    - 0 mark if non respondents described
    - 0 mark if rate different and no designation

Table S5. Raw data of probability samples included in meta-analysis

Study	Setting	Disease	Data collection period	Cohort study	Sample size	Prevalence	Meta-analysis
Anxiety							
Qian et al 2020**[32]**	Wuhan, China	COVID-19	Feb 1–10, 2020	n/a	510	32.8%	+
Qian et al 2020**[32]**	Shanghai, China	COVID-19	Feb 1–10, 2020	n/a	501	20.5%	+
Zhao et al 2020**[34]**	Hong Kong	COVID-19	Apr 9–23, 2020	n/a	1 501	15.8%†	
Zhao et al 2020**[35]**	Hong Kong	COVID-19	Apr 9–23, 2020	n/a	1 501	15.8%†	+
Choi et al 2020**[30]**	Hong Kong	COVID-19	Apr 24–May 3, 2020	n/a	500	14.0%	+
Holingue et al 2020**[31]**	United States	COVID-19	Mar 10–16, 2020	UAS	5 065	14.7%	+
Bruine de Bruin 2020**[29]**	United States	COVID-19	Mar 10–31, 2020	UAS	6 666	15.5%*	
Twenge et al 2020**[33]**	United States	COVID-19	Apr 23–May 5, 2020	HPS	69 316	30.8%	+
Twenge et al 2020**[33]**	United States	COVID-19	May 7–12, 2020	HPS	39 447	30.0%	
Twenge et al 2020**[33]**	United States	COVID-19	May 14–19, 2020	HPS	119 897	28.2%	
Twenge et al 2020**[33]**	United States	COVID-19	May 21–26, 2020	HPS	90 798	29.4%	
Depression							
Li et al 2020**[41]**	Hong Kong	COVID-19	Feb 25–Apr 29, 2020	n/a	3 011	21.3%	+
Zhao et al 2020**[34]**	Hong Kong	COVID-19	Apr 9–23, 2020	n/a	1 501	14.8%¶	
Zhao et al 2020**[35]**	Hong Kong	COVID-19	Apr 9–23, 2020	n/a	1 501	14.8%¶	+
Choi et al 2020**[30]**	Hong Kong	COVID-19	Apr 24–May 3, 2020	n/a	500	19.8%	+
Garre-Olmo et al 2020**[39]**	Girona, Spain	COVID-19	Apr 8–May 4, 2020	n/a	692	12.7%	+
Holingue et al 2020**[31]**	United States	COVID-19	Mar10–16, 2020	UAS	5 065	9.5%	
Bruine de Bruin 2020**[29]**	United States	COVID-19	Mar 10–31, 2020	UAS	6 666	10.3%*	

Daly et al 2020**[36]**	United States	COVID-19	Mar 10–31, 2020	UAS	6 819	10.5%	+
Ettman et al 2020**[37]**	United States	COVID-19	Mar 31–Apr 13, 2020	AP	1 441	27.8%‡	+
Ettman et al 2020**[38]**	United States	COVID-19	Mar 31–Apr 13, 2020	AP	1 441	27.8%‡	
Daly et al 2020**[36]**	United States	COVID-19	Apr 1–17, 2020	UAS	5 428	14.2%	
Twenge et al 2020**[33]**	United States	COVID-19	Apr 23–May 5, 2020	HPS	69 316	23.5%	+
Twenge et al 2020**[33]**	United States	COVID-19	May 7–12, 2020	HPS	39 447	24.1%	
Twenge et al 2020**[33]**	United States	COVID-19	May 14–19, 2020	HPS	119 897	24.4%	
Twenge et al 2020**[33]**	United States	COVID-19	May 21–26, 2020	HPS	90 798	24.9%	
Ko et al 2006**[40]**	Taiwan	SARS	After epidemic	n/a	1 499	3.7%	
PTSD							
Lau et al 2005**[43]**	Hong Kong	SARS	Toward end of epidemic	n/a	818	15.7%§	+
Lau et al 2006**[44]**	Hong Kong	SARS	Toward end of epidemic	n/a	818	15.7%§	
Jalloh et al 2018**[42]**	Sierra Leone	Ebola	Toward end of epidemic	n/a	3 564	16.0%	+
Psychological distress							
Harris et al 2020**[48]**	Norway	COVID-19	Mar 20–27, 2020	n/a	4 008	1.0%	+
Daly et al 2020**[47]**	United Kingdom	COVID-19	Apr 2020	UKHLS	14 985	37.8%	
Chandola et al 2020**[46]**	United Kingdom	COVID-19	Apr 2020	UKHLS	17 761	37.2%	+
Pierce et al 2020**[55]**	United Kingdom	COVID-19	Apr 23–30, 2020	UKHLS	17 452	27.3%	
Li et al 2020**[50]**	United Kingdom	COVID-19	Apr 24–30, 2020	UKHLS	15 530	29.2%	
Niedzwiedz et al 2020**[53]**	United Kingdom	COVID-19	Apr 24–30, 2020	UKHLS	10 977	30.6%	
Daly et al 2020**[47]**	United Kingdom	COVID-19	May 2020	UKHLS	15 809	34.7%	
Chandola et al 2020**[46]**	United Kingdom	COVID-19	May 2020	UKHLS	14 811	34.7%	
Daly et al 2020**[47]**	United Kingdom	COVID-19	Jun 2020	UKHLS	15 842	31.9%	

Chandola et al 2020**[46]**	United Kingdom	COVID-19	Jun 2020	UKHLS	14 123	32.1%	
Chandola et al 2020**[46]**	United Kingdom	COVID-19	Jul 2020	UKHLS	13 754	25.8%	
Robinson et al 2020**[57]**	United States	COVID-19	Mar 10–18, 2020	UAS	5 549	10.5%	
Bruine de Bruin 2020**[29]**	United States	COVID-19	Mar 10–31, 2020	UAS	6 666	11.2%*	
Riehm et al 2020**[56]**	United States	COVID-19	Mar 10–31, 2020	UAS	6 329	11.3%	
Kämpfen et al 2020**[49]**	United States	COVID-19	Mar 10–31, 2020	UAS	6 585	11.2%	+
Robinson et al 2020**[57]**	United States	COVID-19	Apr 1–14, 2020	UAS	5 146	16.0%	
McGinty et al 2020**[51]**	United States	COVID-19	Apr 7–13, 2020	AP	1 468	13.6%	+
McGinty et al 2020**[52]**	United States	COVID-19	Apr 7–13, 2020	AP	1 337	14.2%	
Robinson et al 2020**[57]**	United States	COVID-19	May 27–Jun 9, 2020	UAS	5 784	9.8%	
McGinty et al 2020**[52]**	United States	COVID-19	Jul 7–22, 2020	AP	1 337	13.0%	
Cénat et al 2020**[45]**	Équateur, Congo	EVD	After epidemic	n/a	1 614	45.6%	
Jalloh et al 2018**[42]**	Sierra Leone	EVD	Toward end of epidemic	n/a	3 564	6%	
Peng et al 2010**[54]**	Taiwan	SARS	After epidemic	n/a	1 278	11.7%	
Suicidality							
Leske, et al 2020**[59]**	Queensland, Australia	COVID-19	Feb–Aug, 2020	n/a	Whole population	Monthly suicide rate: 14.1/100 000	
Qin et al 2020**[60]**	Norway	COVID-19	Mar–May, 2020	n/a	Whole population	Three-month suicide rate: 2.8/100 000	
Wasserman 1992**[61]**	United States	1918 Influenza	During epidemic	n/a	Whole population	Numeric data not available	
Alcohol use disorders							
Jackson et al 2020**[58]**	England, United Kingdom	COVID-19	April, 2020	n/a	1,674	38.3%	

EVD= Ebola Virus Disease. HPS=Household Pulse Survey. SARS= Severe acute respiratory syndrome. AP=AmeriSpeak Panel. UAS=Understanding America Study. UKHLS=UK Household Longitudinal Study. *Data was obtained by contacting the corresponding author. [†,‡,§,¶] Same data reported by the same author.

Table S6. List of countries with published studies on novel epidemics and mental health

**COVID-19**	**Asia (n=104)**
	64 China (2 Entire China, 54 Mainland China, 8 Hong Kong), 8 India, 4 Iran, 4 Japan, 4 Nepal, 4 Saudi Arabia, 3 Israel, 2 Bangladesh, 2 Jordan, 2 South Korea, 1 Kuwait, 1 Malaysia, 1 Oman, 1 Philippines, 1 Singapore, 1 United Arab Emirates, 1 Vietnam
	**Europe (n=84)**
	20 United Kingdom, 14 Italy, 12 Spain, 11 Germany, 4 Greece, 4 Turkey, 3 Austria, 3 Norway, 2 Ireland, 2 Poland, 2 Portugal, 1 Bosnia and Herzegovina, 1 Cyprus, 1 Czech, 1 Denmark, 1 France, 1 Georgia, 1 Sweden
	**North America (n=30)**
	23 United States, 1 United States and Canada, 4 Canada, 2 Mexico
	**Oceania (n=9)**
	6 Australia, 3 New Zealand
	**South America (n=7)**
	4 Brazil, 2 Argentina, 1 Colombia,
	**Africa (n=5)**
	2 Egypt, 1 Morocco, 1 South Africa, 1 Tunisia
	**International (n=11)**
**Ebola**	**Africa (n=2)**
	1 Congo, 1 Sierra Leone
**SARS**	**Asia (n=5)**
	4 China (2 Hong Kong, 2 Taiwan), 1 Singapore
**1918 Influenza**	**North America (n=1)**
	1 United States

Note: The number of studies may exceed 255 as some studies reported on more than one country.

Table S7. Prevalence of mental health outcomes during and after novel epidemics in probability samples or whole populations

Study	Setting	Disease	Phase of epidemic	Study design	Survey method	Participation rate	Age, years	Sample size	Measure	Prevalence (95% CI)
Anxiety										
**Twenge et al 2020[33]**	United States	COVID-19	Before and During	Longitudinal	In-person; Online	NR	≥18	Before: 17 067; During: 39 447-119 897	GAD-2≥3	Before: 8.2%; During: T1: 30.8%, T2: 30.0%, T3: 28.2%, T4: 29.4%[†]
**Zhao et al 2020[35]**	Hong Kong	COVID-19	Before and During	Serial cross-sectional	Online; Telephone	Before: 70.2-74.4%; During: 61.3%	≥18	Before: 4 036-4 051; During: 1 501	GAD-2≥3	Before: T1: 11.3%, T2: 9.3%; During: 15.8%[a]
**Qian et al 2020[32]**	Wuhan and Shanghai, China	COVID-19	During	Cross-sectional	Telephone	13.8%	≥18	Wuhan: 510, Shanghai: 501	GAD-7≥10	Wuhan: 32.8%; Shanghai: 20.5%
**Choi et al 2020[30]**	Hong Kong	COVID-19	During	Cross-sectional	Online	64.6%	≥18	500	GAD-7≥10	14.0%
**Holingue et al 2020[31]**	United States	COVID-19	During	Cross-sectional	Online	63%	≥18	5,065	GAD-2≥3	14.7%[‡]
**Bruine de Bruin 2020[29]**	United States	COVID-19	During	Cross-sectional	Online	79%	18-100	6,666	GAD-2≥3	15.5%[b, ‡]
Depression										
**Twenge et al 2020[33]**	United States	COVID-19	Before and During	Longitudinal	In-person; Online	NR	≥18	Before: 17 067; During: 39 447-119 897	PHQ-2≥3	Before: 6.6%; During: T1: 23.5%, T2: 24.1%, T3: 24.4%, T4: 24.9%[†]
**Daly et al 2020[36]**	United States	COVID-19	Before and During	Longitudinal	Before: In-person; During: Online	Before: NR; During: 63.9-80.2%	≥18	5 428-6 819	PHQ-2≥3	Before: 8.9% (7.8-10.1); During: T1: 10.5%, T2: 14.2%[‡]
**Zhao et al 2020[35]**	Hong Kong	COVID-19	Before and During	Serial Cross-sectional	Online; Telephone	Before: 70.2-74.4%; During: 61.3%	≥18	Before: 4 036-4 051; During: 1 501	PHQ-2≥3	Before: T1: 7.2%, T2: 6.3%; During: 14.8%
**Ettman et al 2020[37]**	United States	COVID-19	Before and During	Serial Cross-sectional	Online	64.3%	≥18	Before: 5 065; During: 1 441	PHQ-9≥10	Before: 8.5%; During: 27.8%[a],[*]
**Choi et al 2020[30]**	Hong Kong	COVID-19	During	Cross-sectional	Online	64.6%	≥18	500	PHQ-9≥10	19.8%
**Li et al 2020[41]**	Hong Kong	COVID-19	During	Cross-sectional	Telephone	71.4%	≥15	3 011	PHQ-9≥10	21.3% (19.9-22.8)
**Garre-Olmo et al 2020[39]**	Girona, Spain	COVID-19	During	Cross-sectional	Online	90.4%	≥18	692	PHQ-9≥10	12.7% (10.3-15.4)
**Holingue et al 2020[31]**	United States	COVID-19	During	Cross-sectional	Online	63.0%	≥18	5 065	PHQ-2≥3	9.5%[‡]

**Bruine de Bruin 2020[29]**	United States	COVID-19	During	Cross-sectional	Online	79%	18-100	6 666	PHQ-2≥3	10.3%[b, ‡]
**Ko et al 2006[40]**	Taiwan	SARS	After	Cross-sectional	Telephone	NR	≥15	1 499	TDQ≥18	3.7%
Post-traumatic stress disorder										
**Jalloh et al 2018[42]**	Sierra Leone	EVD	During	Cross-sectional	In-person	97.9%	Median: 35	3 564	IES-R≥33	16% (14.7-17.1)
**Lau et al 2005[43]**	Hong Kong	SARS	During	Serial cross-sectional	Telephone	57.7%	18-60	818	IES≥28	15.7%[a]
Psychological distress										
**Chandola et al 2020[46]**	United Kingdom	COVID-19	During	Longitudinal	Online	39.2-49%	NR	13 754-17 761	GHQ-12≥3	T1: 37.2%, T2: 34.7%, T3: 32.1%, T4: 25.8%[§]
**Daly et al 2020[47]**	United Kingdom	COVID-19	Before and During	Longitudinal	Before: In-person/online; During: Online	Before: 67.9%; During: 46.0-48.6%	18-96	14 393	GHQ-12≥3	Before: 24.3%; During: T1: 37.8%, T2: 34.7%, T3: 31.9%[§]
**Niedzwiedz et al 2020[53]**	United Kingdom	COVID-19	Before and During	Longitudinal	Online	Before: ≥80%; During: 48.6%	≥18	Before: 22 823; During: 9 748	GHQ-12≥4	Before: 19.4% (18.7-20.1); During: 30.6% (29.1-32.3)[§]
**Pierce et al 2020[55]**	United Kingdom	COVID-19	Before and During	Longitudinal	Before: In-person; During: Online	Before: NR; During: 41.2%	≥16	Before: 12 312; During: 17 452	GHQ-12≥4	Before: 18.9% (17.8-20.0); During: 27.3% (26.3-28.2)[§]
**McGinty et al 2020[52]**	United States	COVID-19	During	Longitudinal	Online	T1: NR, T2: 91.2%	≥18	1 337	K6≥13	T1: 14.2% (11.3-17.7), T2: 13.0% (10.1-16.5)[*]
**Robinson et al 2020[57]**	United States	COVID-19	During	Longitudinal	Online	NR	≥18	5 146-5 784	PHQ-4≥6	T1: 10.5%, T2: 16.0%, T6: 9.8%[‡]
**McGinty et al 2020[51]**	United States	COVID-19	Before and During	Serial cross-sectional	Online	Before: 64.2%, During: 70.4%	≥18	Before: 25 417; During: 1 468	K6≥13	Before: 3.9% (3.6-4.2), During: 13.6% (11.1-16.5)[*]
**Harris et al 2020[48]**	Norway	COVID-19	During	Cross-sectional	Online	NR	≥18	4 008	HSCL-10≥1.85	<1%
**Li et al 2020[50]**	United Kingdom	COVID-19	During	Cross-sectional	Online	41.2%	≥18	15 530	GHQ-12≥4	29.2%
**Kämpfen et al 2020[49]**	United States	COVID-19	During	Cross-sectional	Online	78.1%	≥18	6 585	PHQ-4≥6	11.2%[‡]
**Bruine de Bruin 2020[29]**	United States	COVID-19	During	Cross-sectional	Online	79%	18-100	6 666	PHQ-4≥6	11.2%[b, ‡]
**Riehm et al 2020[56]**	United States	COVID-19	During	Cross-sectional	In-person	81.6%	≥18	6 329	PHQ-4≥6	11.3%[‡]

**Cénat et al 2020[45]**	Équateur, Congo	EVD	After	Cross-sectional	In-person	98.6%	18-85	1 614	K10≥22	45.6% (42.0-49.2)
**Jalloh et al 2018[42]**	Sierra Leone	EVD	During	Cross-sectional	In-person	97.9%	≥15	3 564	PHQ-4≥6	6% (5.4-7.0)
**Peng et al 2010[54]**	Taiwan	SARS	After	Cross-sectional	Telephone	68.3%	18-89	1 278	BSRS-5≥6	11.7%
Suicidality										
**Leske, et al 2020[59]**	Queensland, Australia	COVID-19	Before and During	Time series	Secondary data analysis	NA	NA	NA	Monthly suicide rates	Before: 14.9/100 000; During: 14.1/100 000
**Qin et al 2020[60]**	Norway	COVID-19	Before and During	Time series	Secondary data analysis	NA	NA	NA	3-month suicide rates	Year 2014-2018: 2.9-4.1/100 000; Year 2020: 2.8/100 000
**Wasserman 1992[61]**	United States	1918 Influenza	Before, During and After	Time series	Secondary data analysis	NA	NA	NA	Monthly suicide rates	Increased with mortality due to outbreak.
Alcohol use disorders										
**Jackson et al 2020[58]**	England, United Kingdom	COVID-19	Before and During	Serial cross-sectional	Before lockdown: In-person; During lockdown: Telephone	NR	≥16	Before lockdown: 18 884; During lockdown: 1 674	AUDIT-C≥5	Before lockdown: 25.1%; During lockdown: 38.3%

COVID-19=Coronavirus Disease 2019. EVD=Ebola Virus Disease. SARS=Severe acute respiratory syndrome. NA=not applicable. NR=not reported. Measures: AUDIT-C=Alcohol Use Disorders Identification Test-Concise. BRSR-5=5-item Brief Symptom Rating Scale. GAD-2=Generalized Anxiety Disorder-2 Scale. GAD-7=Generalized Anxiety Disorder-7 Scale. GHQ-12=General Health Questionnaire-12. HSCL-10=10-item Hopkins Symptom Checklist. IES=Impact of Event Scale. IES-R=Impact of Event Scale-Revised. K6=Kessler Psychological Distress Scale-6. K10=Kessler Psychological Distress Scale-10. PHQ-2=Patient Health Questionnaire-2. PHQ-4=Patient Health Questionnaire-4. PHQ-9=Patient Health Questionnaire-9. TDQ=Taiwanese Depression Questionnaire. [a]Same data was reported by the same author in another study, which is omitted from the table.[b]Numerical data was obtained by contacting the corresponding author. [*]NORC's AmeriSpeak panel. [†]Household Pulse Survey. [‡]Understanding America Study. [§]UK Household Longitudinal Study.

Table S8. Prevalence of mental health outcomes during and after novel epidemics in non-probability samples of general population

Study	Setting	Disease	Study design	Survey method	Age range, years	Sample size	Measure	Cut-off (Binary)	Prevalence (95% CI)
Anxiety									
Dawel et al 2020[230]	Australia	COVID-19	Longitudinal	Online	≥18	1 295	GAD-7	≥10	20.3%
Bendau et al 2020[231]	Germany	COVID-19	Longitudinal	Online	18-82	1 822	GAD-2	≥3	T1: 36.4%, T2: 29.2%, T3: 24.5%, T4: 24.9%
Gopal et al 2020[232]	India	COVID-19	Longitudinal	Online	≥18	159	GAD-7	≥10	T1: 29.2%, T4: 38.7%
O'Connor et al 2020[96]	United Kingdom	COVID-19	Longitudinal	Online	≥18	3 077	GAD-7	≥10	T1: 21.0%, T2: 18.6%, T3: 16.8%
Winkler et al 2020[98]	Czech	COVID-19	Serial cross-sectional	Online; Telephone	≥18	6 327	M.I.N.I.		Before: 3.1% (2.52-3.72); During: 5.2% (4.31-5.95)
Abba-Aji et al 2020[233]	Alberta, Canada	COVID-19	Cross-sectional	Online	11-88	6 041	GAD-7	≥10	46.7%
Badellino et al 2020[147]	Argentina	COVID-19	Cross-sectional	Online	≥18	1 985	GAD-7	≥10	15.1%
Fisher et al 2020[165]	Australia	COVID-19	Cross-sectional	Online	≥18	13 829	GAD-7	≥10	21.0%
Hammarberg et al 2020[152]	Australia	COVID-19	Cross-sectional	Online	≥18	13 829	GAD-7	≥10	20.0%
Pieh et al 2020[234]	Austria	COVID-19	Cross-sectional	Online	≥18	1 005	GAD-7	≥10	19.0%
Pieh et al 2020[235]	Austria	COVID-19	Cross-sectional	Online	≥18	1 005	GAD-7	≥10	19.0%
Hossain et al 2020[163]	Bangladesh	COVID-19	Cross-sectional	Online	≥18	880	GAD-7	≥10	49.1%
Islam et al 2020[153]	Bangladesh	COVID-19	Cross-sectional	Online	13-63	1 311	GAD-7	≥10	37.3%
Martinez et al 2020[154]	Brazil	COVID-19	Cross-sectional	Online	≥18	1 613	HADS	≥8	82.6%
Elton-Marshall et al 2020[63]	Canada	COVID-19	Cross-sectional	Online	≥18	1 005	GAD-7	≥10	25.5%
Nwachukwu et al 2020[236]	Canada	COVID-19	Cross-sectional	Online	≥18	8 267	GAD-7	≥10	47.2%
Ahmed et al 2020[237]	China	COVID-19	Cross-sectional	Online	14-68	1 074	BAI	≥16	18.9%
Zhao et al 2020[161]	China	COVID-19	Cross-sectional	Online	≥13	2 003	BAI	≥19	9.4%
Hou et al 2020[238]	China	COVID-19	Cross-sectional	Online	≥18	3 088	GAD-2	≥3	13.3%
Ni et al 2020[66]	Wuhan, China	COVID-19	Cross-sectional	Online	≥18	1 577	GAD-2	≥3	23.8% (21.8-26.0)
Zhang et al 2020[239]	China	COVID-19	Cross-sectional	Online	≥18	1 255	GAD-2	≥3	8.5%
Ni et al 2020[164]	China	COVID-19	Cross sectional	Online	≥18	2 551	GAD-7	≥10	40.3%
Li et al 2020[240]	China	COVID-19	Cross-sectional	Online	NR	3 001	GAD-7	≥10	2.9%
Huang et al 2020[168]	China	COVID-19	Cross-sectional	Online	Mean: 35.3	7 236	GAD-7	≥9	35.1%
Gao et al 2020[64]	China	COVID-19	Cross-sectional	Online	Mean: 32.3	4 872	GAD-7	≥10	22.6% (21.4-23.8)
Lin et al 2020[241]	China	COVID-19	Cross sectional	Online	NR	5 641	GAD-7	≥10	18.5%
Zhang et al 2020[242]	China	COVID-19	Cross-sectional	Online	Mean: 29.6	98	GAD-7	≥10	23.4%
Ren et al 2020[243]	China	COVID-19	Cross-sectional	Online	NR	1 172	GAD-7	NR	13.3%

Liang et al 2020[244]	China	COVID-19	Cross-sectional	Online	NR	Hubei: 30; Others: 1 074	GAD-7	≥10	Hubei: 16.7%; Others: 9.2%
Ren et al 2020[171]	China	COVID-19	Cross sectional	Online	≥16	6 130	GAD-7	≥10	7.1%
Shi et al 2020[94]	China	COVID-19	Cross-sectional	Online	≥18	56 932	GAD-7	≥10	10.4%
Su et al 2020[245]	China	COVID-19	Cross-sectional	Online	≥18	403	GAD-7	≥10	14.7%
Wang et al 2020[79]	China	COVID-19	Cross sectional	Online; Telephone	≥11	19 372	GAD-7	≥10	12.2% (11.8-12.7)
Zhong et al 2020[166]	China	COVID-19	Cross-sectional	Online	16-87	7 741	GAD-7	≥6	23.6%
Zhang et al 2020[80]	Shandong, China	COVID-19	Cross sectional	Online	≥11	3 237	GAD-7	≥10	20.8%
Fu et al 2020[150]	Wuhan, China	COVID-19	Cross-sectional	Online	≥18	1 242	GAD-7	≥10	27.5%
Lu et al 2020[167]	Wuhan, China	COVID-19	Cross-sectional	Online	≥18	1 035	GAD-7	≥10	21.2%
Wu et al 2020[246]	China	COVID-19	Cross-sectional	Online	NR	24 789	HADS	≥11	17.8%
Guo et al 2020[91]	China	COVID-19	Cross-sectional	Online	≥18	2 331	HADS	≥8	25.4%
Huang et al 2020[74]	China	COVID-19	Cross-sectional	Online	≥18	6 261	SAS	≥50	13.5%
Liu et al 2020[247]	China	COVID-19	Cross-sectional	Online	≥18	4 911	SAS	≥50	20.6%
Lei et al 2020[248]	Southwest, China	COVID-19	Cross-sectional	Online	Mean: 32.3	1 593	SAS	≥50	8.3%
Wang et al 2020[144]	China	COVID-19	Cross-sectional	Online	18-72	600	SAS	≥50	6.3%
Zhao et al 2020[249]	China	COVID-19	Cross sectional	Online	NR	515	SAS	≥50	14.4%
Zhu et al 2020[250]	China	COVID-19	Cross sectional	Online	11-75	992	SAS	≥50	9.6%
Elhai et al 2020[251]	Tianjin, China	COVID-19	Cross sectional	Online	17-64	908	GAD-7	≥9	7.9%
Lau et al 2020[252]	Hong Kong	COVID-19	Cross-sectional	Online	18-79	761	GAD-2	≥2	26.9%
Solomou et al 2020[253]	Cyprus	COVID-19	Cross-sectional	Online	≥18	1 642	GAD-7	≥10	23.1%
Makhashvili et al 2020[72]	Georgia	COVID-19	Cross-sectional	Online	≥18	2 088	GAD-7	≥10	23.5%
Benke et al 2020[67]	Germany	COVID-19	Cross-sectional	Online	Mean: 40.5	4 335	PROMIS-ED-SF	≥20	30.8%
Bauerle et al 2020[254]	Germany	COVID-19	Cross sectional	Online	≥18	15 037	GAD-2	≥3	19.7%
Petzold et al 2020[255]	Germany	COVID-19	Cross-sectional	Online	18-99	6 509	GAD-2	≥3	25%
Bäuerle et al 2020[256]	Germany	COVID-19	Cross-sectional	Online	≥18	15 037	GAD-7	≥10	16.8%
Benke et al 2020[257]	Germany	COVID-19	Cross-sectional	Online	18-95	4 335	GAD-7	≥10	21.2%
Hetkamp et al 2020[258]	Germany	COVID-19	Cross-sectional	Online	≥18	16 245	GAD-7	≥10	7.2%
Teufel et al 2020[259]	Germany	COVID-19	Cross-sectional	Online	NR	12 244	GAD-7	NR	10%
Skoda et al 2020[260]	Germany	COVID-19	Cross-sectional	Online	NR	10 629	GAD-7	≥10	16.0%
Skapinakis et al 2020[76]	Greece	COVID-19	Cross-sectional	Online	≥18	3 379	GAD-2	≥3	27.7%
Parlapani et al 2020[261]	Greece	COVID-19	Cross-sectional	Online	≥18	3 029	GAD-7	≥11	35.6%
Papandreou et al 2020[262]	Greece	COVID-19	Cross-sectional	Online	≥18	839	GAD-7	≥10	13.2%
Parimala et al 2020[263]	India	COVID-19	Cross-sectional	Online	16-81	956	GAD-7	NR	7.11%
Gupta et al 2020[264]	India	COVID-19	Cross-sectional	Online	≥18	958	GAD-2	≥3	11.7%
Grover et al 2020[265]	India	COVID-19	Cross-sectional	Online	≥18	1 685	GAD-7	≥10	38.2%

Shukla et al 2020[266]	India	COVID-19	Cross-sectional	Online	14-87	1 685	GAD-7	≥10	23.7%
Mirhosseini et al 2020[267]	Shahroud, Iran	COVID-19	Cross-sectional	Online	Mean: 35.3	3 565	GAD-7	≥10	18.5%
Hyland et al 2020[181]	Ireland	COVID-19	Cross-sectional	Online	18-88	1 041	GAD-7	≥10	20.0%
Palgi et al 2020[71]	Israel	COVID-19	Cross-sectional	Online	≥18	1 059	GAD-7	≥10	19.0%
Gualano et al 2020[151]	Italy	COVID-19	Cross-sectional	Online	≥18	1 515	GAD-2	≥3	23.2%
Casagrande et al 2020[149]	Italy	COVID-19	Cross-sectional	Online	18-89	2 291	GAD-7	≥10	32.1%
Landi et al 2020[268]	Italy	COVID-19	Cross-sectional	Online	≥18	944	GAD-7	≥10	18.1%
Pakenham et al 2020[269]	Italy	COVID-19	Cross-sectional	Online	≥18	1 035	GAD-7	≥10	15.3%
Mollaioli et al 2020[155]	Italy	COVID-19	Cross-sectional	Online	≥18	6 821	GAD-7	≥10	23.8%
Ueda et al 2020[160]	Japan	COVID-19	Cross-sectional	Online	≥18	2 000	GAD-7	≥10	10.9%
Massad et al 2020[270]	Jordan	COVID-19	Cross-sectional	Online	≥18	5 274	BAI	≥16	16.9%
Naser et al 2020[145]	Jordan	COVID-19	Cross-sectional	Online	≥18	1 798	GAD-7	≥10	22.8%
Burhamah et al 2020[148]	Kuwait	COVID-19	Cross-sectional	Online	≥18	4 132	GAD-7	≥8	25.3%
Janati Idrissi et al 2020[158]	Morocco	COVID-19	Cross-sectional	Online	Mean: 35	827	HAM-A	≥18	29.5%
Gupta et al 2020[271]	Nepal	COVID-19	Cross-sectional	Online	NR	62	GAD-7	≥10	19.4%
Every-Palmer et al 2020[183]	New Zealand	COVID-19	Cross-sectional	Online	18-90	2 010	GAD-7	≥10	15.6%
Havnen et al 2020[272]	Norway	COVID-19	Cross-sectional	Online	≥18	617	GAD-7	≥10	12.0%
Al Sinawi et al 2020[180]	Oman	COVID-19	Cross-sectional	Online	≥18	1 538	GAD-7	≥10	22.0%
Alhalafi et al 2020[273]	Riyadh, Saudi Arabia	COVID-19	Cross-sectional	Online	≥18	651	GAD-7	≥10	25.5%
Shatla et al 2020[159]	Saudi Arabia	COVID-19	Cross-sectional	Online	NR	1 921	HADS	NR	26.4%
Lee et al 2020[274]	Seoul, South Korea	COVID-19	Cross-sectional	Online	≥20	1 049	GAD-2	≥3	18.6%
Ozdemir et al 2020[275]	Singapore	COVID-19	Cross-sectional	Online	≥21	897	GAD-7	≥10	23.8%
González-Sanguino et al 2020[276]	Spain	COVID-19	Cross-sectional	Online	18-80	3 480	GAD-2	≥3	21.6%
Fullana et al 2020[277]	Spain	COVID-19	Cross-sectional	Online	≥18	5 545	GAD-7	≥10	15.0%
Jacques-Avino et al 2020[69]	Spain	COVID-19	Cross-sectional	Online	≥18	7 053	GAD-7	≥10	27.3%
Papandreou et al 2020[262]	Spain	COVID-19	Cross-sectional	Online	≥18	1 002	GAD-7	≥10	12.3%
McCracken et al 2020[278]	Sweden	COVID-19	Cross-sectional	Online	≥18	1 212	GAD-7	≥10	24.2%
Ozdemir et al 2020[279]	Turkey	COVID-19	Cross-sectional	Online	20-75	2 301	BAI	≥19	24.7%
Özdin et al 2020[280]	Turkey	COVID-19	Cross-sectional	Online	≥18	343	HADS	≥7	45.1%
Thomas et al 2020[157]	United Arab Emirates	COVID-19	Cross-sectional	Online	≥18	1 039	GAD-7	≥10	55.7%
Shevlin et al 2020[281]	United Kingdom	COVID-19	Cross-sectional	Online	≥18	2 025	GAD-7	≥10	21.6%
Dawson et al 2020[282]	United Kingdom	COVID-19	Cross-sectional	Online	18-76	555	GAD-7	≥10	27.0%

Iob et al 2020[283]	United Kingdom	COVID-19	Cross-sectional	Online	≥18	44 775	GAD-7	≥10	20.8%
Jia et al 2020[70]	United Kingdom	COVID-19	Cross-sectional	Online	≥18	3 097	GAD-7	≥10	26.0%
Groarke et al 2020[284]	United Kingdom	COVID-19	Cross-sectional	Online	18-87	1 964	GAD-7	≥10	30.3%
Pieh et al 2020[285]	United Kingdom	COVID-19	Cross-sectional	Online	≥18	1 006	GAD-7	≥10	39.0%
Rettie et al 2020[286]	United Kingdom	COVID-19	Cross-sectional	Online	≥18	842	GAD-7	≥10	24.3%
Shevlin et al 2020[89]	United Kingdom	COVID-19	Cross-sectional	Online	≥18	2 025	GAD-7	≥10	21.6 % (19.8–23.4)
Iob et al 2020[78]	United Kingdom	COVID-19	Cross-sectional	Online	≥18	51 417	PHQ-9	≥10	40.0%
Meyer et al 2020[287]	United States	COVID-19	Cross-sectional	Online	≥18	3 052	BAI	≥22	7.1%
Lee et al 2020[274]	United States	COVID-19	Cross-sectional	Online	18-65	256	GAD-2	≥3	57.4%
Czeisler et al 2020[115]	United States	COVID-19	Cross-sectional	Online	≥18	5 470	GAD-2	≥3	25.5%
Lee et al 2020[288]	United States	COVID-19	Cross-sectional	Online	≥18	453	GAD-2	≥3	47.0%
Fitzpatrick et al 2020[289]	United States	COVID-19	Cross-sectional	Online	≥18	10 368	GAD-7	≥10	>25%
Sherman et al 2020[162]	United States	COVID-19	Cross-sectional	Online	≥18	591	GAD-7	≥10	16.6%
Passos et al 2020[290]	Brazil and Portugal	COVID-19	Cross-sectional	Online	≥18	550	GAD-7	≥10	28.1%
Taylor et al 2020[291]	United States and Canada	COVID-19	Cross-sectional	Online	18-94	6 854	GAD-2	≥3	28%
Nelson et al 2020[292]	International	COVID-19	Cross-sectional	Online	≥18	2 062	GAD-2	≥3	60.7%
Pouso et al 2020[68]	International	COVID-19	Cross-sectional	Online	≥18	5 218	GAD-2	≥3	23.9%
Alzueta et al 2020[293]	International	COVID-19	Cross-sectional	Online	18-94	6 882	GAD-7	≥10	19.5%
Barzilay et al 2020[73]	International	COVID-19	Cross-sectional	Online	18-79	1 350	GAD-7	≥10	22.0%
Généreux et al 2020[294]	International	COVID-19	Cross-sectional	Online	≥18	8 806	GAD-7	≥10	21.0%
Prout et al 2020[295]	International	COVID-19	Cross-sectional	Online	≥18	2 236	GAD-7	≥8	4.6%
Lee et al 2020[296]	International	COVID-19	Cross-sectional	Online	18-65	1 237	GAD-7	≥10	36.0%
Ng et al 2020[297]	Hong Kong	COVID-19	Case control	Telephone	Mean: 57.8	45	HADS	≥11	6.7%
Salari et al 2020[298]	Iran	COVID-19	Case-control	Online	NR	900	BAI	≥24	22.2%
Depression									
Dawel et al 2020[230]	Australia	COVID-19	Longitudinal	Online	≥18	1 295	PHQ-9	≥10	16.4%
Bendau et al 2020[231]	Germany	COVID-19	Longitudinal	Online	18-82	1 822	PHQ-2	≥3	T1: 32.7%, T2: 30.5%, T3: 25.2%, T4: 25.3%
Gopal et al 2020[232]	India	COVID-19	Longitudinal	Online	≥18	159	PHQ-2	≥3	T2: 14.8%, T4: 26.1%,
O'Connor et al 2020[96]	United Kingdom	COVID-19	Longitudinal	Online	≥18	3 077	PHQ-9	≥10	T1: 26.1%, T2: 24.3%, T3: 23.7%
Schmitz et al 2020[299]	Quebec, Canada	COVID-19	Serial cross-sectional	Online	≥18	Before: 52 996; During: 1 607	PHQ-8	≥10	Before: 6.8%; During: 19.2%
Winkler et al 2020[98]	Czech	COVID-19	Serial cross-sectional	Online	≥18	6 327	M.I.N.I.	NR	Before: 4.0% (3.3-4.6); During: 11.8% (10.6-13.0)
Sønderskov et al 2020[300]	Denmark	COVID-19	Serial cross-sectional	Online	Mean: 49.1	2 458	WHO-5	≤49	25.4%
Badellino et al 2020[147]	Argentina	COVID-19	Cross-sectional	Online	≥18	1 985	PHQ-9	≥9	24.4%

Fisher et al 2020[165]	Australia	COVID-19	Cross-sectional	Online	≥18	13 829	PHQ-9	≥10	27.6%
Hammarberg et al 2020[152]	Australia	COVID-19	Cross-sectional	Online	≥18	13 829	PHQ-9	≥10	24.8%
Pieh et al 2020[234]	Austria	COVID-19	Cross-sectional	Online	≥18	1 005	PHQ-9	≥10	21.0%
Pieh et al 2020[235]	Austria	COVID-19	Cross-sectional	Online	≥18	1 005	PHQ-9	≥10	21.0%
Sljivo et al 2020[169]	Bosnia and Herzegovina	COVID-19	Cross-sectional	Online	≥18	1 213	PHQ-9	≥10	28.4%
Martinez et al 2020[154]	Brazil	COVID-19	Cross-sectional	Online	≥18	1 613	HADS	≥8	48.8%
Passos et al 2020[290]	Brazil	COVID-19	Cross-sectional	Online	≥18	289	PHQ-2	≥3	26.6%
Nwachukwu et al 2020[236]	Canada	COVID-19	Cross-sectional	Online	≥18	8 267	PHQ-9	≥10	44.1%
Abba-Aji et al 2020[233]	Alberta, Canada	COVID-19	Cross-sectional	Online	11-88	6 041	PHQ-9	≥10	41.4%
Ahmed et al 2020[237]	China	COVID-19	Cross-sectional	Online	14-68	1 074	BDI	≥14	37.1%
Tang et al 2020[170]	China	COVID-19	Cross-sectional	Online	≥18	941	CES-D	≥15	19.8%
Huang et al 2020[168]	China	COVID-19	Cross-sectional	Online	Mean: 35.3	7 236	CES-D	≥28	20.1%
Wu et al 2020[246]	China	COVID-19	Cross-sectional	Online	NR	24 789	HADS	≥11	14.2%
Hou et al 2020[238]	China	COVID-19	Cross-sectional	Online	≥18	3 088	PHQ-2	≥3	14.1%
Ni et al 2020[66]	Wuhan, China	COVID-19	Cross-sectional	Online	≥18	1 577	PHQ-2	≥3	19.2% (17.3-21.2)
Zhang et al 2020[239]	China	COVID-19	Cross-sectional	Online	≥18	1 255	PHQ-2	≥3	9.5%
Lin et al 2020[241]	China	COVID-19	Cross-sectional	Online	NR	5 641	PHQ-9	≥10	24.5%
Zhang et al 2020[242]	China	COVID-19	Cross-sectional	Online	Mean: 29.6	98	PHQ-9	≥10	34.7%
Ren et al 2020[243]	China	COVID-19	Cross-sectional	Online	NR	1 172	PHQ-9	NR	18.8%
Huang et al 2020[74]	China	COVID-19	Cross-sectional	Online	≥18	6 261	PHQ-9	≥10	17.2%
Li et al 2020[240]	China	COVID-19	Cross-sectional	Online	NR	3 001	PHQ-9	≥10	3.0%
Liang et al 2020[244]	China	COVID-19	Cross-sectional	Online	NR	Hubei: 30; Others: 1 074	PHQ-9	≥10	Hubei: 23.3%; Others: 18.3%
Ren et al 2020[171]	China	COVID-19	Cross-sectional	Online	≥16	6 130	PHQ-9	≥10	12%
Shi et al 2020[94]	China	COVID-19	Cross-sectional	Online	≥18	56 932	PHQ-9	≥10	10.8%
Wang et al 2020[79]	China	COVID-19	Cross-sectional	Online; Telephone	≥11	19 372	PHQ-9	≥10	11.0% (10.6-11.5)
Zhang et al 2020[174]	China	COVID-19	Cross-sectional	Online	≥18	1 342	PHQ-9	≥10	13.6%
Zhong et al 2020[166]	China	COVID-19	Cross-sectional	Online	16-87	7 741	PHQ-9	≥7	21.5%
Liu et al 2020[301]	Guangdong, China	COVID-19	Cross-sectional	Online	NR	727	PHQ-9	≥10	11.4%
Zhang et al 2020[80]	Shandong, China	COVID-19	Cross-sectional	Online	≥11	3 237	PHQ-9	≥10	19.5%
Peng et al 2020[302]	Shenzhen, China	COVID-19	Cross-sectional	In-person	18-70	2 237	SDS	≥50	6.2%
Jiang et al 2020[303]	China	COVID-19	Cross-sectional	Online	≥18	60 199	SDS	≥48	64.3%
Liu et al 2020[304]	China	COVID-19	Cross-sectional	Online	NR	608	SDS	≥50	27.1%
Wang et al 2020[144]	China	COVID-19	Cross-sectional	Online	18-72	600	SDS	≥53	17.2%
Zhao et al 2020[249]	China	COVID-19	Cross-sectional	Online	NR	515	SDS	≥53	29.7%

Lei et al 2020[248]	Southwest, China	COVID-19	Cross-sectional	Online	Mean: 32.3	1 593	SDS	≥40	14.6%
Gao et al 2020[64]	China	COVID-19	Cross-sectional	Online	Mean: 32.3	4 872	WHO-5	≤12	48.3% (46.9-49.7)
Chen et al 2020[305]	Wuhan and surrounding areas, China	COVID-19	Cross-sectional	Online	NR	1 071	SDS	≥53	2.9%
Fu et al 2020[150]	Wuhan, China	COVID-19	Cross-sectional	Online	≥18	1 242	PHQ-9	≥10	29.3%
Lu et al 2020[167]	Wuhan, China	COVID-19	Cross-sectional	Online	≥18	1 035	PHQ-9	≥10	16.7%
Lau et al 2020[252]	Hong Kong	COVID-19	Cross-sectional	Online	18-79	761	PHQ-2	≥3	18.3%
Bressington et al 2020[75]	Hong Kong	COVID-19	Cross-sectional	Online	18-59	11 072	PHQ-9	≥10	46.5%
Caballero-Dominguez et al 2020[97]	Colombia	COVID-19	Cross-sectional	Online	18-76	700	WHO-5	≤9	61.1%
Peretti-Watel et al 2020[65]	France	COVID-19	Cross-sectional	Online	≥18	2 003	PHQ-9	≥15	8.8% (7.6-10.0)
Makhashvili et al 2020[72]	Georgia	COVID-19	Cross-sectional	Online	≥18	2 088	PHQ-9	≥10	29.6%
Munk et al 2020[95]	Germany	COVID-19	Cross-sectional	Online	≥18	949	BDI-II	≥13	35.5%
Bäuerle et al 2020[254]	Germany	COVID-19	Cross-sectional	Online	≥18	15 037	PHQ-2	≥3	14.3%
Bäuerle et al 2020[256]	Germany	COVID-19	Cross-sectional	Online	≥18	15 037	PHQ-2	≥3	14.3%
Tuefel et al 2020[259]	Germany	COVID-19	Cross-sectional	Online	NR	12 244	PHQ-2	NR	11.9%
Petzold et al 2020[255]	Germany	COVID-19	Cross-sectional	Online	18-99	6 509	PHQ-2	≥3	25%
Benke et al 2020[257]	Germany	COVID-19	Cross-sectional	Online	18-95	4 335	PHQ-9	≥10	31.1%
Benke et al 2020[67]	Germany	COVID-19	Cross-sectional	Online	Mean: 40.5	4 335	PHQ-9	≥10	31.0%
Parlapani et al 2020[261]	Greece	COVID-19	Cross-sectional	Online	≥18	3 029	PHQ-9	≥10	22.7%
Skapinakis et al 2020[76]	Greece	COVID-19	Cross-sectional	Online	≥18	3 379	PHQ-9	≥10	27.7%
Voitsidis et al 2020[306]	Greece	COVID-19	Cross-sectional	Online	≥18	2 827	PHQ-9	≥10	23.2%
Papandreou et al 2020[262]	Greece	COVID-19	Cross-sectional	Online	≥18	839	PHQ-9	≥10	18.8%
Gupta et al 2020[264]	India	COVID-19	Cross-sectional	Online	≥18	958	PHQ-2	≥3	11.1%
Grover et al 2020[265]	India	COVID-19	Cross-sectional	Online	≥18	1 685	PHQ-9	≥10	10.5%
Shukla et al 2020[266]	India	COVID-19	Cross-sectional	Online	14-87	1 685	PHQ-9	≥10	8.8%
Singh et al 2020[307]	India	COVID-19	Cross-sectional	Online	≥18	234	PHQ-9	≥10	14.1%
Hyland et al 2020[181]	Ireland	COVID-19	Cross-sectional	Online	18-88	1 041	PHQ-9	≥10	22.8%
Castelli et al 2020[175]	Italy	COVID-19	Cross-sectional	Online	Mean: 35.1	1 321	BDI-II	NR	31%
Shapiro et al 2020[308]	Israel	COVID-19	Cross-sectional	Online	18-90	503	PHQ-2	≥3	12.5%
Palgi et al 2020[71]	Israel	COVID-19	Cross-sectional	Online	≥18	1 059	PHQ-9	≥10	14.4%
Gualano et al 2020[151]	Italy	COVID-19	Cross-sectional	Online	≥18	1 515	PHQ-2	≥3	24.7%
Landi et al 2020[268]	Italy	COVID-19	Cross-sectional	Online	≥18	944	PHQ-9	≥10	23.4%
Pakenham et al 2020[269]	Italy	COVID-19	Cross-sectional	Online	≥18	1 035	PHQ-9	≥10	21.7%
Mollaioli et al 2020[155]	Italy	COVID-19	Cross-sectional	Online	≥18	6 821	PHQ-9	≥10	30.0%
Sugaya et al 2020[309]	Japan	COVID-19	Cross-sectional	Online	≥18	11 333	PHQ-9	≥10	17.9%
Stickley et al 2020[310]	Japan	COVID-19	Cross-sectional	Online	≥18	2 000	PHQ-9	≥10	17.4%

Ueda et al 2020[160]	Japan	COVID-19	Cross-sectional	Online	≥18	2 000	PHQ-9	≥10	17.3%
Naser et al 2020[145]	Jordan	COVID-19	Cross-sectional	Online	≥18	1 798	PHQ-9	≥10	32.1%
Burhamah et al 2020[148]	Kuwait	COVID-19	Cross-sectional	Online	≥18	4 132	PHQ-9	≥10	30.1%
Janati Idrissi et al 2020[158]	Morocco	COVID-19	Cross-sectional	Online	Mean: 35	827	BDI	≥8	14.0%
Gupta et al 2020[271]	Nepal	COVID-19	Cross-sectional	Online	NR	62	PHQ-9	≥10	8.1%
Ghimire et al 2020[311]	Nepal	COVID-19	Cross-sectional	Online	≥18	556	WHO-5	≤13	40.1%
Shrestha et al 2020[312]	Nepal	COVID-19	Cross-sectional	Online	NR	556	WHO-5	≤13	40.1%
Havnen et al 2020[272]	Norway	COVID-19	Cross-sectional	Online	≥18	617	PHQ-9	≥10	20.6%
Al Sinawi et al 2020[180]	Oman	COVID-19	Cross-sectional	Online	≥18	1 538	PHQ-9	≥12	35.0%
Bodecka et al 2020[313]	Poland	COVID-19	Cross-sectional	Online	15-73	230	PHQ-9	≥10	45.6%
Passos et al 2020[290]	Portugal	COVID-19	Cross-sectional	Online	≥18	261	PHQ-2	≥3	22.6%
Alhalafi et al 2020[273]	Riyadh, Saudi Arabia	COVID-19	Cross-sectional	Online	≥18	651	PHQ-9	≥10	28.7%
Shatla et al 2020[159]	Saudi Arabia	COVID-19	Cross-sectional	Online	NR	1 921	HADS	NR	37.3%
Kim et al 2020[314]	South Africa	COVID-19	Cross-sectional	Online	≥25	221	CES-D	≥10	14.5%
Lee et al 2020[315]	Seoul, South Korea	COVID-19	Cross-sectional	Online	≥20	1 049	PHQ-2	≥3	29.6%
Kim et al 2020[316]	South Korea	COVID-19	Cross-sectional	Online	19-65	550	PHQ-9	≥10	20.2%
González-Sanguino et al 2020[276]	Spain	COVID-19	Cross-sectional	Online	18-80	3 480	PHQ-2	≥3	18.7%
Fullana et al 2020[277]	Spain	COVID-19	Cross-sectional	Online	≥18	5 545	PHQ-9	≥10	15.0%
Jacques-Avino et al 2020[69]	Spain	COVID-19	Cross-sectional	Online	≥18	7 053	PHQ-9	≥10	25.0%
Papandreou et al 2020[262]	Spain	COVID-19	Cross-sectional	Online	≥18	1 002	PHQ-9	≥10	13.6%
McCracken et al 2020[278]	Sweden	COVID-19	Cross-sectional	Online	≥18	1 212	PHQ-9	≥10	30.0%
Ozdemir et al 2020[279]	Turkey	COVID-19	Cross-sectional	Online	20-75	2 301	BDI	≥18	21.8%
Karasar et al 2020[317]	Turkey	COVID-19	Cross-sectional	Online	≥18	518	BDI	≥17	16.6%
Ustun 2020[318]	Turkey	COVID-19	Cross-sectional	Online	18-65	1 115	BDI	≥17	27.3%
Özdin et al 2020[280]	Turkey	COVID-19	Cross-sectional	Online	≥18	343	HADS	≥10	23.6%
Thomas et al 2020[157]	United Arab Emirates	COVID-19	Cross-sectional	Online	≥18	1 039	PHQ-8	≥10	58.4%
Dawson et al 2020[282]	United Kingdom	COVID-19	Cross-sectional	Online	18-76	555	PHQ-9	≥10	37.0%
Iob et al 2020[283]	United Kingdom	COVID-19	Cross-sectional	Online	≥18	44 775	PHQ-9	≥10	29.5%
Jia et al 2020[70]	United Kingdom	COVID-19	Cross-sectional	Online	≥18	3 097	PHQ-9	≥10	31.6%
Groarke et al 2020[284]	United Kingdom	COVID-19	Cross-sectional	Online	18-87	1 964	PHQ-9	≥10	34.0%
Pieh et al 2020[234]	United Kingdom	COVID-19	Cross-sectional	Online	≥18	1 006	PHQ-9	≥10	41.2%
Rettie et al 2020[286]	United Kingdom	COVID-19	Cross-sectional	Online	≥18	842	PHQ-9	≥10	25.8%
Shevlin et al 2020[89]	United Kingdom	COVID-19	Cross-sectional	Online	≥18	2 025	PHQ-9	≥10	22.1 % (20.3–23.9)

Meyer et al 2020[287]	United States	COVID-19	Cross-sectional	Online	≥18	3 052	BDI-II	≥20	10.1%
Lee et al 2020[274]	United States	COVID-19	Cross-sectional	Online	18-65	256	PHQ-2	≥3	53.9%
Czeisler et al 2020[115]	United States	COVID-19	Cross-sectional	Online	≥18	5 470	PHQ-2	≥3	24.3%
Kolacz et al 2020[84]	United States	COVID-19	Cross-sectional	Online	≥18	1 666	PHQ-2	≥3	28.7%
Lee et al 2020[288]	United States	COVID-19	Cross-sectional	Online	≥18	453	PHQ-2	≥3	45.3%
Bryan et al 2020[77]	United States	COVID-19	Cross-sectional	Online	≥18	10 625	PHQ-9	≥14	19.5%
Knell et al 2020[319]	United States	COVID-19	Cross-sectional	Online	≥18	1 809	PHQ-9	≥6	18.5%
Sherman et al 2020[162]	United States	COVID-19	Cross-sectional	Online	≥18	591	PHQ-9	≥10	21%
Taylor et al 2020[291]	United States and Canada	COVID-19	Cross-sectional	Online	18-94	6 854	PHQ-2	≥3	22%
Lee et al 2020[296]	International	COVID-19	Cross-sectional	Online	18-65	1 237	PHQ-9	≥10	40.3%
Barzilay et al 2020[73]	International	COVID-19	Cross-sectional	Online	18-79	1 350	PHQ-2	≥3	16.0%
Nelson et al 2020[292]	International	COVID-19	Cross-sectional	Online	≥18	2 062	PHQ-2	≥3	44.5%
Pouso et al 2020[68]	International	COVID-19	Cross-sectional	Online	≥18	5 218	PHQ-2	≥3	27.8%
Généreux et al 2020[294]	International	COVID-19	Cross-sectional	Online	≥18	8 806	PHQ-9	≥10	25.5%
Prout et al 2020[295]	International	COVID-19	Cross-sectional	Online	≥18	2 236	PHQ-9	≥10	7.0%
Ng et al 2020[297]	Hong Kong	COVID-19	Case-control	Telephone	Mean: 57.8	45	HADS	≥11	6.7%
Post-traumatic Stress Disorder									
Planchuelo-Gomez et al 2020[320]	Spain	COVID-19	Longitudinal	Online	≥18	3 668	IES	NR	T1: NR, T2: 11.8%
Winkler et al 2020[98]	Czech	COVID-19	Serial cross-sectional	Online	≥18	6 327	M.I.N.I.	NR	Before: 1.0% (0.6-1.3); During: 1.7% (1.2-2.2)
Shuwiekh et al 2020[321]	Arab countries	COVID-19	Cross-sectional	Online	≥18	1 374	PCL-5	≥31	36.6%
Gurvich et al 2020[322]	Australia	COVID-19	Cross-sectional	Online	≥18	1 495	IES-R	≥33	47.0%
Traunmuller et al 2020[323]	Austria	COVID-19	Cross-sectional	Online	16-82	4 126	IES-R	≥33	43.3%
Campos et al 2020[176]	Brazil	COVID-19	Cross-sectional	Online	≥18	12 196	IES-R	≥33	35.2%
Cao et al 2020[324]	Shanghai, China	COVID-19	Cross-sectional	NR	≥18	430	IES	≥26	68.4%
Ma et al 2020[325]	China	COVID-19	Cross-sectional	Online	≥18	728	IES	≥26	25.5%
Zhang et al 2020[326]	Liaoning, China	COVID-19	Cross-sectional	Online	Mean: 37.7	263	IES	≥26	7.6%
Wang et al 2020[327]	China	COVID-19	Cross-sectional	Paper	12-59	1 210	IES-R	≥33	53.8%
Jiang et al 2020[328]	China	COVID-19	Cross-sectional	Online	Mean: 25.8	338	PCL-5	≥38	3.5%
Liu et al 2020[329]	Wuhan and surrounding cities, China	COVID-19	Cross-sectional	Online	≥18	285	PCL-5	≥33	7%
Lu et al 2020[167]	Wuhan, China	COVID-19	Cross-sectional	Online	≥18	1 035	PCL-C	≥50	4.5%
Zhao et al 2020[249]	China	COVID-19	Cross-sectional	Online	NR	515	PCL-5	DSM diagnostic algorithm	5.6%

Ren et al 2020[243]	China	COVID-19	Cross-sectional	Online	NR	458	PCL-5	NR	7.0%
Lau et al 2020[252]	Hong Kong	COVID-19	Cross-sectional	Online	18-79	761	IES-R	≥33	28.6%
El-Zoghby et al 2020[330]	Egypt	COVID-19	Cross-sectional	Online	≥18	510	IES-R	≥33	52.0%
Ahuja et al 2020[331]	India	COVID-19	Cross-sectional	Online	≥18	325	IES	≥26	100.0%
Singh et al 2020[307]	India	COVID-19	Cross-sectional	Online	≥18	234	IES-R	>33	13.5%
Varshney et al 2020[332]	India	COVID-19	Cross-sectional	Online	18-82	653	IES-R	≥33	18.2%
Mohammadi et al 2020[81]	Iran	COVID-19	Cross-sectional	Online	NR	1 881	IES-R	≥33	52.7%
Karatzias et al 2020[178]	Ireland	COVID-19	Cross-sectional	Online	≥18	1 041	ITQ	≥2	17.7%
Lahav et al 2020[333]	Israel	COVID-19	Cross-sectional	Online	≥18	976	PCL-5	≥33	11.5%
Forte et al 2020[82]	Italy	COVID-19	Cross-sectional	Online	18-89	2 291	IES-R	≥33	27.7%
Costantini et al 2020[179]	Italy	COVID-19	Cross-sectional	Online	21-71	329	IES-R	≥50	13.4%
Di Giuseppe et al 2020[334]	Italy	COVID-19	Cross-sectional	Online	≥18	5 683	IES-R	≥33	29.4%
Micarelli et al 2020[335]	Italy	COVID-19	Cross-sectional	Online	23-72	1 380	IES-R	≥33	21.5%
Forte et al 2020[336]	Italy	COVID-19	Cross-sectional	Online	18-74	2 286	PCL-5	DSM diagnostic algorithm	27.5%
Castelli et al 2020[175]	Italy	COVID-19	Cross-sectional	Online	Mean: 35.1	1 321	PCL-5	NR	20%
Cortés-Álvarez et al 2020[337]	Mexico	COVID-19	Cross-sectional	Online	≥18	1 105	IES-R	≥33	50.3%
Ramirez et al 2020[177]	Mexico	COVID-19	Cross-sectional	Online	≥18	3 932	IES-R	≥35	27.7%
Tee et al 2020[338]	Philippines	COVID-19	Cross-sectional	Online	≥12	1 879	IES-R	≥34	16.3%
Paulino et al 2020[339]	Portugal	COVID-19	Cross-sectional	Online	18-95	10 529	IES-R	≥33	49.2%
Alkhamees et al 2020[340]	Saudi Arabia	COVID-19	Cross-sectional	Online	≥18	1 160	IES-R	≥33	23.6%
Alshehri et al 2020[85]	Saudi Arabia	COVID-19	Cross-sectional	Online	≥18	1 374	PCL-S	≥45	19.6%
Lee et al 2020[315]	Seoul, South Korea	COVID-19	Cross-sectional	Online	≥20	1 049	PC-PTSD-5	≥3	14.0%
Odriozola-Gonzalez et al 2020[341]	Spain	COVID-19	Cross-sectional	Online	≥18	3 550	IES-R	NR	47.5%
Rodriguez-Rey et al 2020[342]	Spain	COVID-19	Cross-sectional	Online	≥18	3 055	IES-R	≥33	36.6%
Rodríguez-Rey et al 2020[343]	Spain	COVID-19	Cross-sectional	Online	18-88	3 055	IES-R	≥33	36.6%
Fekih-Romdhane et al 2020[83]	Tunisia	COVID-19	Cross-sectional	Online	≥18	603	IES-R	≥33	33.0%
Groarke et al 2020[284]	United Kingdom	COVID-19	Cross-sectional	Online	18-87	1 964	PCL-5	≥34	19.4%
Czeisler et al 2020[115]	United States	COVID-19	Cross-sectional	Online	≥18	5 470	IES-6	≥1.75 out of 4	26.3%
Kolacz et al 2020[84]	United States	COVID-19	Cross-sectional	Online	≥18	1 666	PCL-5	DSM Diagnostic algorithm	27.8%

Sherman et al 2020[162]	United States	COVID-19	Cross-sectional	Online	≥18	591	PCL-5	≥33	5.4%
Le et al 2020[344]	Vietnam	COVID-19	Cross-sectional	Online	Mean: 35	1 423	IES-R	≥33	10.8%
Prout et al 2020[295]	International	COVID-19	Cross-sectional	Online	≥18	2 236	IES-R	≥33	4.3%
Hao et al 2020[345]	Chongqing, China	COVID-19	Case-control	Online	Mean: 33.1	109	IES-R	≥24	13.8%
Guo et al 2020[346]	China	COVID-19	Case-control	Online	18-75	103	PCL-5	≥33	1.9%
Sim et al 2010[347]	Singapore	SARS	Cross-sectional	Paper	Mean: 36.6	415	IES-R	DSM diagnostic algorithm	25.8%
Psychological Distress									
Kikuchi et al 2020[185]	Japan	COVID-19	Longitudinal	Online	≥18	2 078	K6	≥13	Before: 9.3% During: 11.3%
Sibley et al 2020[348]	New Zealand	COVID-19	Longitudinal	Online	NR	1 003	K6	≥13	Before: 6.6%; After: 5.8%
Winkler et al 2020[98]	Czech	COVID-19	Serial cross-sectional	Online; Telephone	≥18	6 327	M.I.N.I.	NR	Before: 20.0% (18.6-21.4); During: 29.6% (27.9-31.4)
Nicolson et al 2020[349]	New Zealand	COVID-19	Serial cross-sectional	Online	≥18	During: 1 190; After: 925	PHQ-4	NR	During: 8%; After: 5%
Gray et al 2020[350]	United Kingdom	COVID-19	Serial cross-sectional	Online	≥16	Before: 11 922; During: 12 989	K10	≥25	36.9%
Twenge et al 2020[351]	United States	COVID-19	Serial cross-sectional	In-person; Online	≥18	Before: 2 032; During: 19330	K6	≥13	Before: 3.4%; During: 27.7%
Fernandez et al 2020[352]	Argentina	COVID-19	Cross-sectional	Online	18-92	4 408	BSI-53	*T*≥63	27.1%
Rahman et al 2020[182]	Australia	COVID-19	Cross-sectional	Online	≥18	587	K10	≥22	33.3%
Duarte et al 2020[88]	Rio Grande do Sul, Brazil	COVID-19	Cross-sectional	Online	18-75	799	SRQ-20	≥7	40.9%
Qiu et al 2020[213]	China	COVID-19	Cross-sectional	Online	NR	52 730	CPDI	≥52	5.1%
Zhong et al 2020[166]	China	COVID-19	Cross-sectional	Online	16-87	7 741	GHQ-12	≥3	30.0%
Yu et al 2020[353]	China	COVID-19	Cross-sectional	Online	≥18	1 588	K6	≥13	22.8%
Goodwin et al 2020[184]	China	COVID-19	Cross-sectional	Online	≥18	1 135	K6	≥13	19.1%
Ben-Ezra et al 2020[93]	China	COVID-19	Cross-sectional	Online	18-59	1 134	K6	≥13	19.1%
Goodwin et al 2020[354]	China	COVID-19	Cross-sectional	Online	Mean: 31.0	1 134	K6	≥13	19.1%
Liu et al 2020[304]	China	COVID-19	Cross-sectional	Online	NR	455	SCL-90	≥160	7.7%
Tian et al 2020[355]	China	COVID-19	Cross-sectional	Online	13-76	1 060	SCL-90	≥63	12.5%
Zhu et al 2020[90]	China	COVID-19	Cross-sectional	Online	NR	836	SRQ-20	≥7	13.4%
Lau et al 2020[356]	Hong Kong	COVID-19	Cross-sectional	Online	18-79	761	PHQ-4	≥6	17.8%
El-Abasiri et al 2020[357]	Egypt	COVID-19	Cross-sectional	Online	≥18	257	CPDI	≥52	21.8%
Liu et al 2020[358]	Germany	COVID-19	Cross-sectional	Online	18-81	1 007	CPDI	≥52	3.6%
Petzold et al 2020[255]	Germany	COVID-19	Cross-sectional	Online	18-99	6 509	PHQ-4	≥6	25%
Venugopal et al 2020[359]	India	COVID-19	Cross-sectional	Online	≥18	453	GHQ-28	≥24	42.2%
Jahanshahi et al 2020[360]	Iran	COVID-19	Cross-sectional	Online	≥18	1 058	CPDI	≥52	61.1%

Mohammadi et al 2020[81]	Iran	COVID-19	Cross-sectional	Online	NR	1 881	GHQ-28	≥6	38.5%
Costantini et al 2020[179]	Italy	COVID-19	Cross-sectional	Online	21-71	329	CPDI	≥52	4.9%
Pakenham et al 2020[269]	Italy	COVID-19	Cross-sectional	Online	≥18	1 035	CPDI	≥52	1.6%
Fiorillo et al 2020[361]	Italy	COVID-19	Cross-sectional	Online	≥18	20 720	GHQ-12	≥4	91.2%
Di Giuseppe et al 2020[334]	Italy	COVID-19	Cross-sectional	Online	≥18	5 683	SCL-90	GSI>0.8	35.6%
Moccia et al 2020[362]	Italy	COVID-19	Cross-sectional	Online	≥18	500	K10	≥25	18.6%
Forte et al 2020[82]	Italy	COVID-19	Cross-sectional	Online	≥18	2 291	SCL-90	≥0.9	31.4%
Sugaya et al 2020[309]	Japan	COVID-19	Cross-sectional	Online	18-89	11 333	K6	≥13	11.5%
Shrestha et al 2020[363]	Nepal	COVID-19	Cross-sectional	Online	17-83	410	CPDI	≥52	0.5%
Every-Palmer et al 2020[183]	New Zealand	COVID-19	Cross-sectional	Online	18-90	2 010	K10	≥12	30.3%
Al Sinawi et al 2020[180]	Oman	COVID-19	Cross-sectional	Online	≥18	1 538	GAD-7 and PHQ-9	GAD-7≥10 or PHQ-9≥12	30.0%
Perez et al 2020[364]	Spain	COVID-19	Cross-sectional	Online	18-91	1 781	BSI-18	GSI≥63	24.6%
Dominguez-Salas et al 2020[87]	Spain	COVID-19	Cross-sectional	Online	≥18	4 180	GHQ-12	≥3	72.0%
Gómez-Salgado et al 2020[86]	Spain	COVID-19	Cross-sectional	Online	≥18	4 180	GHQ-12	≥3	72.0%
Shevlin et al 2020[89]	United Kingdom	COVID-19	Cross-sectional	Online	≥18	2 025	GAD-7 and PHQ-9	both≥10	27.6%
Goodwin et al 2020[184]	United Kingdom	COVID-19	Cross-sectional	Online	≥18	1 293	K6	≥13	16.6%
Ben-Ezra et al 2020[92]	United Kingdom	COVID-19	Cross-sectional	Online	18-75	1 293	K6	≥13	16.6%
Pouso et al 2020[68]	International	COVID-19	Cross-sectional	Online	≥18	5 218	PHQ-4	≥6	22.2%
Schnell et al 2020[365]	International	COVID-19	Cross-sectional	Online	≥18	1 538	PHQ-4	≥6	19%
Sim et al 2010[347]	Singapore	SARS	Cross-sectional	Paper	Mean: 36.6	415	GHQ-28	≥5	22.9%
Acute Stress Disorder									
Shi et al 2020[94]	China	COVID-19	Cross-sectional	Online	≥18	56 932	ASDS	Dissociation score ≥9 and Cumulative reexperiencing, avoidance, and arousal score ≥28	24.4%
Lin et al 2020[241]	China	COVID-19	Cross-sectional	Online	NR	5 641	ASDS	Disassociation ≥5, and total score≥28	15.8%
Agoraphobia									

Winkler et al 2020[98]	Czech	COVID-19	Serial cross-sectional	Online; Telephone	≥18	6 327	M.I.N.I.	NR	Before: 5.2% (4.4-5.9); During: 8.0% (7.0-9.0)
Alcohol Use Disorders									
Ahmed et al 2020[237]	China	COVID-19	Cross-sectional	Online	Mean: 33.5	1 074	AUDIT	≥20	1.6%
Winkler et al 2020[98]	Czech	COVID-19	Serial cross-sectional	Online; Telephone	≥18	6 327	M.I.N.I.	NR	Before: 10.8% (9.8-11.9); During: 9.9% (8.7-11.0)
Chodkiewicz et al 2020[366]	Poland	COVID-19	Cross-sectional	Online	18-68	443	AUDIT	≥20	0.9%
Obsessive-Complusive disorder									
Munk et al 2020[95]	Germany	COVID-19	Cross-sectional	Online	≥18	949	OCI-R	≥21	21.4%
Fiorillo et al 2020[361]	Italy	COVID-19	Cross-sectional	Online	≥18	20 720	OCI-R	≥21	11.3%
Panic Disorder									
Winkler et al 2020[98]	Czech	COVID-19	Serial cross-sectional	Online; Telephone	≥18	6 327	M.I.N.I.	NR	Before: 0.2% (0.04-0.36); During: 0.9% (0.53-1.18)
Social Phobia									
Winkler et al 2020[98]	Czech	COVID-19	Serial cross-sectional	Online; Telephone	≥18	6 327	M.I.N.I.	NR	Before: 1.7% (1.2-2.1); During: 2.5% (1.9-3.1)
Suicidality (Suicidal Ideation)									
Ren et al 2020[243]	China	COVID-19	Cross-sectional	Online	NR	1 172	M.I.N.I.	NR	2.8%
Caballero-Domínguez et al 2020[97]	Colombia	COVID-19	Cross-sectional	Online	18-76	700	CES-D	≥9	7.6%
Fiorillo et al 2020[361]	Italy	COVID-19	Cross-sectional	Online	≥18	20 720	SIDAS	≥21	14.2%
Gratz et al 2020[367]	United States	COVID-19	Cross-sectional	Online	20-74	500	DSI-SS	≥3	11.6%
Suicidality (Suicide Attempts)									
Fitzpatrick et al 2020[368]	United States	COVID-19	Cross-sectional	Online	≥18	10 368	SBQ-R	≥5	25.0%
Bryan et al 2020[77]	United States	COVID-19	Cross-sectional	Online	≥18	10 625	SITBI	Positive response	1.2%

ASDS=Acute Stress Disorder Scale. AUDIT=Alcohol Use Disorder Identification Test. BAI=Beck Anxiety Inventory. BDI=Beck Depression Inventory. BDI-II=Beck Depression Inventory-II. BSI-18=Brief Symptom Inventory-18. BSI-53=Brief Symptom Inventory-53. CES-D=Centre for Epidemiological Studies-Depression Scale. COVID-19=Coronavirus Disease 2019. CPDI=COVID-19 Peri-traumatic Distress Index. DSI-SS=Depression Symptom Index-Suicide Subscale. GAD-2=Generalized Anxiety Disorder-2. GAD-7=Generalized Anxiety Disorder-7. GHQ-12=General Health Questionnaire-12. GHQ-28=General Health Questionnaire-28. HADS=Hospital Anxiety and Depression Scale. HAM-A=Hamilton Anxiety Rating Scale. IES=Impact of Event Scale. IES-6=Impact of Event Scale-6. IES-R=Impact of Event Scale-Revised. ITQ=International Trauma Questionnaire. K6=Kessler Psychological Distress Scale-6. K10=Kessler Psychological Distress Scale-10. M.I.N.I.=Mini-International Neuropsychiatric Interview. OCI-R=Obsessive-Compulsive Inventory-Revised. PC-PTSD-5=Primary Care Post-Traumatic Stress Disorder Screen for DSM-5. PCL-5= Post-traumatic Stress Disorder Checklist for DSM-5. PCL-C=Post-traumatic Stress Disorder Checklist-Civilian Version. PCL-S=Post-traumatic Stress Disorder Checklist-Specific Version. PHQ-2=Patient Health Questionnaire-2. PHQ-4=Patient Health Questionnaire-4. PHQ-8=Patient Health Questionnaire-8. PHQ-9=Patient Health Questionnaire-9. PROMIS-ED-SF=PROMIS-Emotional Distress-Short Form. SARS=severe acute respiratory syndrome. SAS=Zung's Self-Rating Anxiety Scale. SBQ-R=Suicide Behaviors Questionnaire-Revised. SCL-90=Symptom Checklist-90. SDS=Zung's Self-Rating Depression Scale. SIDAS=Suicidal Ideation Attributes Scale. SITBI=Self-Injurious Thoughts and Behaviors Interview. SRQ-20=Self-Reporting Questionnaire-20. WHO-5=Five-item World Health Organization-Well-Being Index. NA=not applicable. NR=not reported.

Table S9. Study quality ratings based on adapted Newcastle-Ottawa Scale

Cross-Sectional Studies								
**Study**	**Total NOS Score**	**Quality (Low 0-3, Medium 4-6, High 7-9)**	**Representativeness of exposed sample**	**Selection of nonexposed sample**	**Ascertainment of exposure**	**Comparability (controlled for age)**	**Comparability (controlled for additional factors)**	**Assessment of outcome**
Abba-Aji et al 2020**[233]**	2	Low	+	-	-	-	-	+
Ahmed et al 2020**[237]**	2	Low	+	-	-	-	-	+
Ahuja et al 2020**[331]**	2	Low	+	-	-	-	-	+
Al Sinawi et al 2020**[180]**	4	Medium	+	-	-	+	+	+
Alhalafi 2020**[273]**	4	Medium	+	-	-	+	+	+
Alkhamees et al 2020**[340]**	2	Low	+	-	-	-	-	+
Alshehri et al 2020**[85]**	4	Medium	+	-	-	+	+	+
Alzueta et al 2020**[293]**	4	Medium	+	-	-	+	+	+
Badellino et al 2020**[147]**	4	Medium	+	-	-	+	+	+
Barzilay et al 2020**[73]**	4	Medium	+	-	-	+	+	+
Bäuerle et al 2020**[254]**	4	Medium	+	-	-	+	+	+
Bäuerle et al 2020**[256]**	2	Low	+	-	-	-	-	+
Ben-Ezra et al 2020**[92]**	4	Medium	+	-	-	+	+	+
Ben-Ezra et al 2020**[93]**	4	Medium	+	-	-	+	+	+
Benke et al**[257]**	4	Medium	+	-	-	+	+	+
Benke et al 2020**[67]**	4	Medium	+	-	-	+	+	+
Bodecka et al 2020**[313]**	2	Low	+	-	-	-	-	+
Bressington et al 2020**[75]**	4	Medium	+	-	-	+	+	+

Bruine de Bruin 2020**[29]**	4	Medium	+	-	-	+	+	+
Bryan et al 2020**[77]**	4	Medium	+	-	-	+	+	+
Burhamah et al 2020**[148]**	4	Medium	+	-	-	+	+	+
Caballero-Domínguez et al 2020**[97]**	3	Low	+	-	-	+	+	-
Campos et al 2020**[176]**	4	Medium	+	-	-	+	+	+
Cao et al 2020**[324]**	2	Low	+	-	-	-	-	+
Casagrande et al 2020**[149]**	4	Medium	+	-	-	+	+	+
Castelli et al 2020**[175]**	4	Medium	+	-	-	+	+	+
Cénat et al 2020**[45]**	4	Medium	+	-	-	+	+	+
Chen et al 2020**[305]**	4	Medium	+	-	-	+	+	+
Chodkiewicz et al 2020**[366]**	2	Low	+	-	-	-	-	+
Choi et al 2020**[30]**	4	Medium	+	-	-	+	+	+
Cortés-Álvarez et al 2020**[337]**	2	Low	+	-	-	-	-	+
Costantini et al 2020**[179]**	4	Medium	+	-	-	+	+	+
Czeisler et al 2020**[115]**	4	Medium	+	-	-	+	+	+
Dai et al 2020**[369]**	4	Medium	+	-	-	+	+	+
Dawel et al 2020**[230]**	4	Medium	+	-	-	+	+	+
Dawson et al 2020**[282]**	4	Medium	+	-	-	+	+	+
Di Giuseppe et al 2020**[334]**	4	Medium	+	-	-	+	+	+
Dominguez-Salas et al 2020**[87]**	4	Medium	+	-	-	+	+	+
Duarte et al 2020**[88]**	4	Medium	+	-	-	+	+	+
El-Abasiri et al 2020**[357]**	4	Medium	+	-	-	+	+	+

Elhai et al 2020**[251]**	2	Low	+	-	-	-	-	+
El-Zoghby et al 2020**[330]**	4	Medium	+	-	-	+	+	+
Elton-Marshall et al 2020 [61]	4	Medium	+	-	-	+	+	+
Ettman et al 2020 [35]	4	Medium	+	-	-	+	+	+
Ettman et al 2020 [36]	4	Medium	+	-	-	+	+	+
Every-Palmer et al 2020**[183]**	4	Medium	+	-	-	+	+	+
Fekih-Romdhane et al 2020**[83]**	4	Medium	+	-	-	+	+	+
Fernandez et al 2020**[352]**	4	Medium	+	-	-	+	+	+
Fiorillo et al 2020**[361]**	4	Medium	+	-	-	+	+	+
Fisher et al 2020**[165]**	4	Medium	+	-	-	+	+	+
Fitzpatrick et al 2020**[289]**	2	Low	+	-	-	-	-	+
Fitzpatrick et al 2020**[368]**	4	Medium	+	-	-	+	+	+
Forte et al 2020**[336]**	2	Low	+	-	-	-	-	+
Forte et al 2020**[82]**	4	Medium	+	-	-	+	+	+
Fu et al 2020**[150]**	4	Medium	+	-	-	+	+	+
Fullana et al 2020**[277]**	4	Medium	+	-	-	+	+	+
Gao et al 2020**[64]**	4	Medium	+	-	-	+	+	+
Garre-Olmo et al 2020**[39]**	4	Medium	+	-	-	+	+	+
Généreux et al 2020**[294]**	4	Medium	+	-	-	+	+	+
Ghimire et al 2020**[311]**	2	Low	+	-	-	-	-	+
Gómez-Salgado et al 2020**[86]**	4	Medium	+	-	-	+	+	+
González-Sanguino et al 2020**[276]**	4	Medium	+	-	-	+	+	+
Goodwin et al 2020**[184]**	4	Medium	+	-	-	+	+	+

Goodwin et al 2020**[354]**	4	Medium	+	-	-	+	+	+
Gratz et al 2020**[367]**	3	Low	+	-	-	+	+	-
Gray et al 2020**[350]**	2	Low	+	-	-	-	-	+
Grey et al 2020**[370]**	4	Medium	+	-	-	+	+	+
Groarke et al 2020**[284]**	4	Medium	+	-	-	+	+	+
Grover et al 2020**[265]**	2	Low	+	-	-	-	-	+
Gualano et al 2020**[151]**	4	Medium	+	-	-	+	+	+
Guo et al 2020**[172]**	4	Medium	+	-	-	+	+	+
Guo et al 2020**[91]**	4	Medium	+	-	-	+	+	+
Gupta et al 2020**[271]**	2	Low	+	-	-	-	-	+
Gupta et al 2020**[264]**	2	Low	+	-	-	-	-	+
Gurvich et al 2020**[322]**	4	Medium	+	-	-	+	+	+
Hammarberg et al 2020**[152]**	4	Medium	+	-	-	+	+	+
Harris et al 2020**[48]**	4	Medium	+	-	-	+	+	+
Havnen et al 2020**[272]**	4	Medium	+	-	-	+	+	+
Hetkamp et al 2020**[258]**	2	Low	+	-	-	-	-	+
Holingue et al 2020**[31]**	4	Medium	+	-	-	+	+	+
Hossain et al 2020**[163]**	4	Medium	+	-	-	+	+	+
Hou et al 2020**[238]**	4	Medium	+	-	-	+	+	+
Huang et al 2020**[168]**	4	Medium	+	-	-	+	+	+
Huang et al 2020**[74]**	4	Medium	+	-	-	+	+	+
Hyland et al 2020**[181]**	4	Medium	+	-	-	+	+	+
Iob et al 2020**[283]**	2	Low	+	-	-	-	-	+

Iob et al 2020**[78]**	4	Medium	+	-	-	+	+	+
Islam et al 2020**[153]**	4	Medium	+	-	-	+	+	+
Jackson et al 2020**[58]**	4	Medium	+	-	-	+	+	+
Jacques-Avino et al 2020**[69]**	4	Medium	+	-	-	+	+	+
Jahanshahi et al 2020**[360]**	4	Medium	+	-	-	+	+	+
Jalloh et al 2018**[42]**	4	Medium	+	-	-	+	+	+
Janati Idrissi et al 2020**[158]**	4	Medium	+	-	-	+	+	+
Jia et al 2020**[70]**	4	Medium	+	-	-	+	+	+
Jiang et al 2020**[303]**	4	Medium	+	-	-	+	+	+
Jiang et al 2020**[328]**	2	Low	+	-	-	-	-	+
Kämpfen et al 2020**[49]**	4	Medium	+	-	-	+	+	+
Karasar et al 2020**[317]**	2	Low	+	-	-	-	-	+
Karatzias et al 2020**[178]**	4	Medium	+	-	-	+	+	+
Kim et al 2020**[316]**	2	Low	+	-	-	-	-	+
Kim et al 2020**[314]**	4	Medium	+	-	-	+	+	+
Knell et al 2020**[319]**	2	Low	+	-	-	-	-	+
Ko et al 2006**[40]**	4	Medium	+	-	-	+	+	+
Kolacz et al 2020**[84]**	4	Medium	+	-	-	+	+	+
Lahav et al 2020**[333]**	4	Medium	+	-	-	+	+	+
Landi et al 2020**[268]**	4	Medium	+	-	-	+	+	+
Lau et al 2005**[43]**	4	Medium	+	-	-	+	+	+
Lau et al 2006**[44]**	4	Medium	+	-	-	+	+	+
Lau et al 2020**[252]**	4	Medium	+	-	-	+	+	+

Lau et al 2020**[356]**	4	Medium	+	-	-	+	+	+
Le et al 2020**[344]**	4	Medium	+	-	-	+	+	+
Lee et al 2020**[371]**	4	Medium	+	-	-	+	+	+
Lee et al 2020**[296]**	4	Medium	+	-	-	+	+	+
Lee et al 2020**[274]**	4	Medium	+	-	-	+	+	+
Lee et al 2020**[315]**	4	Medium	+	-	-	+	+	+
Lee et al 2020**[173]**	4	Medium	+	-	-	+	+	+
Lei et al 2020**[248]**	4	Medium	+	-	-	+	+	+
Leske et al 2020**[59]**	4	Medium	+	-	-	+	+	+
Li et al 2020**[62]**	4	Medium	+	-	-	+	+	+
Li et al 2020**[50]**	4	Medium	+	-	-	+	+	+
Li et al 2020**[240]**	4	Medium	+	-	-	+	+	+
Li et al 2020**[41]**	4	Medium	+	-	-	+	+	+
Liang et al 2020**[244]**	2	Low	+	-	-	-	-	+
Lin et al 2020**[241]**	2	Low	+	-	-	-	-	+
Liu et al 2020**[329]**	4	Medium	+	-	-	+	+	+
Liu et al 2020**[358]**	2	Low	+	-	-	-	-	+
Liu et al 2020**[304]**	2	Low	+	-	-	-	-	+
Liu et al 2020**[247]**	4	Medium	+	-	-	+	+	+
Liu et al 2020**[301]**	4	Medium	+	-	-	+	+	+
Lu et al 2020**[167]**	4	Medium	+	-	-	+	+	+
Ma et al 2020**[325]**	4	Medium	+	-	-	+	+	+
Makhashvili et al 2020**[72]**	4	Medium	+	-	-	+	+	+

Martinez et al 2020**[154]**	4	Medium	+	-	-	+	+	+
Massad et al 2020**[270]**	4	Medium	+	-	-	+	+	+
McCracken et al 2020**[278]**	4	Medium	+	-	-	+	+	+
McGinty et al 2020**[51]**	4	Medium	+	-	-	+	+	+
Meyer et al 2020**[287]**	4	Medium	+	-	-	+	+	+
Micarelli et al 2020**[335]**	2	Low	+	-	-	-	-	+
Mirhosseini et al 2020**[267]**	4	Medium	+	-	-	+	+	+
Moccia et al 2020**[362]**	4	Medium	+	-	-	+	+	+
Mohammadi et al 2020**[81]**	4	Medium	+	-	-	+	+	+
Mollaioli et al 2020**[155]**	4	Medium	+	-	-	+	+	+
Munk et al 2020**[95]**	4	Medium	+	-	-	+	+	+
Naser et al 2020**[145]**	4	Medium	+	-	-	+	+	+
Nelson et al 2020**[292]**	4	Medium	+	-	-	+	+	+
Ni et al 2020**[66]**	4	Medium	+	-	-	+	+	+
Ni et al 2020**[164]**	4	Medium	+	-	-	+	+	+
Nicolson et al 2020**[349]**	4	Medium	+	-	-	+	+	+
Nwachukwu et al 2020**[236]**	4	Medium	+	-	-	+	+	+
Odriozola-Gonzalez et al 2020**[341]**	4	Medium	+	-	-	+	+	+
Ozdemir et al 2020**[275]**	4	Medium	+	-	-	+	+	+
Ozdemir et al 2020**[279]**	4	Medium	+	-	-	+	+	+
Özdin et al 2020**[280]**	3	Low	+	-	-	-	+	+
Pakenham et al 2020**[269]**	4	Medium	+	-	-	+	+	+
Palgi et al 2020**[71]**	4	Medium	+	-	-	+	+	+

Papandreou et al 2020**[262]**	4	Medium	+	-	-	+	+	+
Parimala et al 2020**[263]**	2	Low	+	-	-	-	-	+
Parlapani et al 2020**[261]**	2	Low	+	-	-	-	-	+
Passos et al 2020**[290]**	4	Medium	+	-	-	+	+	+
Paulino et al 2020**[339]**	2	Low	+	-	-	-	-	+
Peng et al 2010**[54]**	4	Medium	+	-	-	+	+	+
Peng et al 2020**[302]**	4	Medium	+	-	-	+	+	+
Peretti-Watel et al 2020**[65]**	4	Medium	+	-	-	+	+	+
Perez et al 2020**[364]**	4	Medium	+	-	-	+	+	+
Petzold et al 2020**[255]**	2	Low	+	-	-	-	-	+
Pieh et al 2020**[234]**	3	Low	+	-	-	+	-	+
Pieh et al 2020**[285]**	3	Low	+	-	-	+	-	+
Pieh et al 2020**[235]**	3	Low	+	-	-	+	-	+
Pouso et al 2020**[68]**	4	Medium	+	-	-	+	+	+
Prout et al 2020**[295]**	4	Medium	+	-	-	+	+	+
Qian et al 2020**[32]**	4	Medium	+	-	-	+	+	+
Qin et al 2020**[60]**	4	Medium	+	-	-	+	+	+
Qiu et al 2020**[213]**	4	Medium	+	-	-	+	+	+
Rahman et al 2020**[182]**	4	Medium	+	-	-	+	+	+
Ramirez et al 2020**[177]**	4	Medium	+	-	-	+	+	+
Ren et al 2020**[243]**	2	Low	+	-	-	-	-	+
Ren et al**[171]**	4	Medium	+	-	-	+	+	+
Rettie et al 2020**[286]**	2	Low	+	-	-	-	-	+

Riehm et al 2020**[56]**	4	Medium	+	-	-	+	+	+
Rodríguez-Rey et al 2020**[343]**	4	Medium	+	-	-	+	+	+
Rodriguez-Rey et al 2020**[342]**	2	Low	+	-	-	-	-	+
Rossi et al 2020**[156]**	4	Medium	+	-	-	+	+	+
Schmitz et al 2020**[299]**	3	Low	+	-	-	+	-	+
Schnell et al 2020**[365]**	4	Medium	+	-	-	+	+	+
Shapiro et al 2020**[308]**	2	Low	+	-	-	-	-	+
Shatla et al 2020**[159]**	3	Low	+	-	-	-	+	+
Sherman et al 2020**[162]**	4	Medium	+	-	-	+	+	+
Shevlin et al 2020**[281]**	4	Medium	+	-	-	+	+	+
Shevlin et al 2020**[89]**	4	Medium	+	-	-	+	+	+
Shi et al 2020**[94]**	4	Medium	+	-	-	+	+	+
Shrestha et al 2020**[312]**	2	Low	+	-	-	-	-	+
Shrestha et al 2020**[363]**	4	Medium	+	-	-	+	+	+
Shukla et al 2020**[266]**	2	Low	+	-	-	-	-	+
Shuwiekh et al 2020**[321]**	2	Low	+	-	-	-	-	+
Sim et al 2010**[347]**	4	Medium	+	-	-	+	+	+
Singh et al 2020**[307]**	2	Low	+	-	-	-	-	+
Skapinakis et al 2020**[76]**	4	Medium	+	-	-	+	+	+
Skoda et al 2020**[260]**	2	Low	+	-	-	-	-	+
Sljivo et al 2020**[169]**	3	Low	+	-	-	-	+	+
Smith et al 2020**[146]**	4	Medium	+	-	-	+	+	+
Solomou et al 2020**[253]**	3	Low	+	-	-	-	+	+

Sønderskov et al 2020**[300]**	4	Medium	+	-	-	+	+	+
Stickley et al 2020**[310]**	4	Medium	+	-	-	+	+	+
Su et al 2020**[245]**	4	Medium	+	-	-	+	+	+
Sugaya et al 2020**[309]**	4	Medium	+	-	-	+	+	+
Tang et al 2020**[170]**	4	Medium	+	-	-	+	+	+
Taylor et al 2020**[291]**	4	Medium	+	-	-	+	+	+
Tee et al 2020**[338]**	2	Low	+	-	-	-	-	+
Thomas et al 2020**[157]**	4	Medium	+	-	-	+	+	+
Tian et al 2020**[355]**	2	Low	+	-	-	-	-	+
Traunmuller et al 2020**[323]**	2	Low	+	-	-	-	-	+
Teufel et al 2020**[259]**	2	Low	+	-	-	-	-	+
Twenge et al 2020**[351]**	4	Medium	+	-	-	+	+	+
Ueda et al 2020**[160]**	4	Medium	+	-	-	+	+	+
Ustun 2020**[318]**	2	Low	+	-	-	-	-	+
Varshney et al 2020**[332]**	2	Low	+	-	-	-	-	+
Venugopal et al 2020**[359]**	2	Low	+	-	-	-	-	+
Voitsidis et al 2020**[306]**	2	Low	+	-	-	-	-	+
Wang et al 2020**[144]**	4	Medium	+	-	-	+	+	+
Wang et al 2020**[79]**	4	Medium	+	-	-	+	+	+
Wang et al 2020**[327]**	2	Low	+	-	-	-	-	+
Wasserman 1992**[61]**	4	Medium	+	-	-	+	+	+
Winkler et al 2020**[98]**	4	Medium	+	-	-	+	+	+
Wu et al 2020**[246]**	4	Medium	+	-	-	+	+	+

Yu et al 2020**[353]**	4	Medium	+	-	-	+	+	+
Zhang et al 2020**[242]**	3	Low	+	-	-	-	+	+
Zhang et al 2020**[372]**	2	Low	+	-	-	-	-	+
Zhang et al 2020**[326]**	4	Medium	+	-	-	+	+	+
Zhang et al 2020**[174]**	4	Medium	+	-	-	+	+	+
Zhang et al 2020**[80]**	4	Medium	+	-	-	+	+	+
Zhao et al 2020**[249]**	2	Low	+	-	-	-	-	+
Zhao et al 2020**[161]**	4	Medium	+	-	-	+	+	+
Zhao et al 2020**[35]**	4	Medium	+	-	-	+	+	+
Zhao et al 2020**[34]**	4	Medium	+	-	-	+	+	+
Zhong et al 2020**[166]**	4	Medium	+	-	-	+	+	+
Zhu et al 2020**[90]**	4	Medium	+	-	-	+	+	+
Zhu et al 2020**[250]**	2	Low	+	-	-	-	-	+

Cohort studies											
**Study**	**Total NOS Score**	**Quality (Low 0-3, Medium 4-6, High 7-9)**	**Adequacy of case definition**	**Represent-ativeness of cases**	**Selection of controls**	**Adequacy of control definition**	**Comparability (controlled for age)**	**Comparability (controlled for additional factors)**	**Ascertain-ment of exposure**	**Same method of ascertainment for cases and controls**	**Non-response rate**
Bendau et al 2020**[231]**	6	Medium	+	-	-	+	+	+	+	+	-
Chandola et al 2020**[46]**	6	Medium	+	-	-	+	+	+	+	+	-
Daly et al 2020**[36]**	7	High	+	-	-	+	+	+	+	+	+
Daly et al 2020**[47]**	7	High	+	-	-	+	+	+	+	+	+
Gopal et al 2020**[232]**	7	High	+	-	-	+	+	+	+	+	+
Kikuchi et al 2020**[185]**	6	Medium	+	-	-	+	+	+	+	+	-
McGinty et al 2020**[52]**	7	High	+	-	-	+	+	+	+	+	+
Niedzwiedz et al 2020**[53]**	6	Medium	+	-	-	+	+	+	+	+	-
O'Connor et al 2020**[96]**	6	Medium	+	-	-	+	+	+	+	+	-
Pierce et al 2020**[55]**	7	High	+	-	-	+	+	+	+	+	+
Planchuelo-Gomez et al 2020**[320]**	6	Medium	+	-	-	+	+	+	+	+	-
Robinson et al 2020**[57]**	7	High	+	-	-	+	+	+	+	+	+
Sibley et al 2020**[348]**	6	Medium	+	-	-	+	+	+	+	+	-
Twenge et al 2020**[33]**	5	Medium	+	-	-	+	+	+	+	-	-

Case-control studies											
**Study**	**Total NOS Score**	**Quality (Low 0-3, Medium 4-6, High 7-9)**	**Adequacy of case definition**	**Represent-ativeness of cases**	**Selection of controls**	**Adequacy of control definition**	**Comparability (controlled for age)**	**Comparability (controlled for additional factors)**	**Ascertain-ment of exposure**	**Same method of ascertainment for cases and controls**	**Non-response rate**
Guo et al 2020**[346]**	4	Medium	-	+	+	+	-	-	-	+	-
Hao et al 2020**[345]**	4	Medium	-	+	+	+	-	-	-	+	-
Ng et al 2020**[297]**	4	Medium	-	+	+	+	-	-	-	+	-
Salari et al 2020**[298]**	4	Medium	-	+	+	+	-	-	-	+	-

References

1. Brooks SK, Smith LE, Webster RK, Weston D, Woodland L, Hall I, et al. The impact of unplanned school closure on children's social contact: rapid evidence review. Euro Surveill. 2020;25:2000188.
2. Chu IY, Alam P, Larson HJ, Lin L. Social consequences of mass quarantine during epidemics: a systematic review with implications for the COVID-19 response. J Travel Med. 2020;27:taaa192.
3. Cooke JE, Eirich R, Racine N, Madigan S. Prevalence of posttraumatic and general psychological stress during COVID-19: a rapid review and meta-analysis. Psychiatry Res. 2020;292:113347.
4. Luo M, Guo L, Yu M, Jiang W, Wang H. The psychological and mental impact of coronavirus disease 2019 (COVID-19) on medical staff and general public - a systematic review and meta-analysis. Psychiatry Res. 2020;291:113190.
5. Salari N, Hosseinian-Far A, Jalali R, Vaisi-Raygani A, Rasoulpoor S, Mohammadi M, et al. Prevalence of stress, anxiety, depression among the general population during the COVID-19 pandemic: a systematic review and meta-analysis. Global Health. 2020;16:57.
6. Wu T, Jia X, Shi H, Niu J, Yin X, Xie J, et al. Prevalence of mental health problems during the COVID-19 pandemic: a systematic review and meta-analysis. J Affect Disord. 2021;281:91-8.
7. Cénat JM, Blais-Rochette C, Kokou-Kpolou CK, Noorishad P-G, Mukunzi JN, McIntee S-E, et al. Prevalence of symptoms of depression, anxiety, insomnia, posttraumatic stress disorder, and psychological distress among populations affected by the COVID-19 pandemic: a systematic review and meta-analysis. Psychiatry Res. 2021;295:113599.
8. Fan FC, Zhang SY, Cheng Y. Incidence of psychological illness after coronavirus outbreak: a meta-analysis study. J Epidemiol Community Health. 2021;75:836-42.
9. Yuan K, Gong YM, Liu L, Sun YK, Tian SS, Wang YJ, et al. Prevalence of posttraumatic stress disorder after infectious disease pandemics in the twenty-first century, including COVID-19: a meta-analysis and systematic review. Mol Psychiatry. 2021;26:4982–98.
10. Pierce M, McManus S, Jessop C, John A, Hotopf M, Ford T, et al. Says who? The significance of sampling in mental health surveys during COVID-19. Lancet Psychiatry. 2020;7:567-8.

11. Brooks SK, Webster RK, Smith LE, Woodland L, Wessely S, Greenberg N, et al. The psychological impact of quarantine and how to reduce it: rapid review of the evidence. Lancet. 2020;395:912-20.
12. Rogers JP, Chesney E, Oliver D, Pollak TA, McGuire P, Fusar-Poli P, et al. Psychiatric and neuropsychiatric presentations associated with severe coronavirus infections: a systematic review and meta-analysis with comparison to the COVID-19 pandemic. Lancet Psychiatry. 2020;7:611-27.
13. Kisely S, Warren N, McMahon L, Dalais C, Henry I, Siskind D. Occurrence, prevention, and management of the psychological effects of emerging virus outbreaks on healthcare workers: rapid review and meta-analysis. BMJ 2020;369:m1642.
14. Leung C, Ho M, Bharwani A, Ni MY. Mental health consequences of viral epidemics in the community: a systematic review. 2020. https://www.crd.york.ac.uk/prospero/display_record.php?RecordID=179105. Accessed 1 September 2020.
15. Else H. How a torrent of COVID science changed research publishing - in seven charts. Nature. 2020;588:553.
16. North CS, Pfefferbaum B. Mental health response to community disasters: a systematic review. JAMA. 2013;310:507-18.
17. Kendell R, Jablensky A. Distinguishing between the validity and utility of psychiatric diagnoses. Am J Psychiatry. 2003;160:4-12.
18. Holmes EA, O'Connor RC, Perry VH, Tracey I, Wessely S, Arseneault L, et al. Multidisciplinary research priorities for the COVID-19 pandemic: a call for action for mental health science. Lancet Psychiatry. 2020;7:547-60.
19. Higgins JPT, Green S. Tools for assessing methodological quality or risk of bias in non-randomized studies. 2012. http://handbook.cochrane.org/chapter_13/13_5_2_3_tools_for_assessing_methodological_quality_or_risk_of.htm. Accessed 1 September 2020.
20. Patra J, Bhatia M, Suraweera W, Morris SK, Patra C, Gupta PC, et al. Exposure to second-hand smoke and the risk of tuberculosis in children and adults: a systematic review and meta-analysis of 18 observational studies. PLoS Med. 2015;12:e1001835.

21. Anglin RES, Samaan Z, Walter SD, McDonald SD. Vitamin D deficiency and depression in adults: systematic review and meta-analysis. Br J Psychiatry. 2013;202:100-7.
22. Ni MY, Kim Y, McDowell I, Wong S, Qiu H, Wong IOL, et al. Mental health during and after protests, riots and revolutions: a systematic review. Aust N Z J Psychiatry. 2020;54:232-43.
23. Guyatt GH, Oxman AD, Vist GE, Kunz R, Falck-Ytter Y, Alonso-Coello P, et al. GRADE: an emerging consensus on rating quality of evidence and strength of recommendations. BMJ. 2008;336:924-6.
24. Rothman KJ, Gallacher JE, Hatch EE. Why representativeness should be avoided. Int J Epidemiol. 2013;42:1012-4.
25. Barendregt JJ, Doi SA, Lee YY, Norman RE, Vos T. Meta-analysis of prevalence. J Epidemiol Community Health. 2013;67:974-8.
26. Deeks JJ, Altman DG, Bradburn MJ. Statistical methods for examining heterogeneity and combining results from several studies in meta-analysis. In: Egger, M, Smith, GD, and Altman, DG (ed). *Systematic Reviews in Health Care: Meta‐Analysis in Context*. BMJ Publishing Group: London, 2001, pp 285-312.
27. Furuya-Kanamori L, Barendregt JJ, Doi SAR. A new improved graphical and quantitative method for detecting bias in meta-analysis. Int J Evid Based Healthc. 2018;16:195-203.
28. Doi SA, Barendregt JJ, Khan S, Thalib L, Williams GM. Advances in the meta-analysis of heterogeneous clinical trials II: the quality effects model. Contemp Clin Trials. 2015;45:123-9.
29. Bruine de Bruin W. Age differences in COVID-19 risk perceptions and mental health: evidence from a national U.S. survey conducted in March 2020. J Gerontol B Psychol Sci Soc Sci. 2021;76:e24-9.
30. Choi EPH, Hui BPH, Wan EYF. Depression and anxiety in Hong Kong during COVID-19. Int J Environ Res Public Health. 2020;17:3740.
31. Holingue C, Kalb LG, Riehm KE, Bennett D, Kapteyn A, Veldhuis CB, et al. Mental distress in the United States at the beginning of the COVID-19 pandemic. Am J Public Health. 2020;110:1628-34.
32. Qian M, Wu Q, Wu P, Hou Z, Liang Y, Cowling BJ, et al. Anxiety levels, precautionary behaviours and public perceptions during the early phase of the COVID-19 outbreak in China: a population-based cross-sectional survey. BMJ Open. 2020;10:e040910.

33. Twenge JM, Joiner TE. U.S. Census Bureau-assessed prevalence of anxiety and depressive symptoms in 2019 and during the 2020 COVID-19 pandemic. Depress Anxiety. 2020;37:954-6.

34. Zhao SZ, Wong JYH, Wu YD, Choi EPH, Wang MP, Lam TH. Social distancing compliance under COVID-19 pandemic and mental health impacts: a population-based study. Int J Environ Res Public Health. 2020;17:6692.

35. Zhao SZ, Wong JYH, Luk TT, Wai AKC, Lam TH, Wang MP. Mental health crisis under COVID-19 pandemic in Hong Kong, China. Int J Infect Dis. 2020;100:431-3.

36. Daly M, Sutin AR, Robinson E. Depression reported by US adults in 2017-2018 and March and April 2020. J Affect Disord. 2021;278:131-5.

37. Ettman CK, Abdalla SM, Cohen GH, Sampson L, Vivier PM, Galea S. Prevalence of depression symptoms in US adults before and during the COVID-19 pandemic. JAMA Netw Open. 2020;3:e2019686.

38. Ettman CK, Abdalla SM, Cohen GH, Sampson L, Vivier PM, Galea S. Low assets and financial stressors associated with higher depression during COVID-19 in a nationally representative sample of US adults. J Epidemiol Community Health. 2021;75:501-8.

39. Garre-Olmo J, Turro-Garriga O, Marti-Lluch R, Zacarias-Pons L, Alves-Cabratosa L, Serrano-Sarbosa D, et al. Changes in lifestyle resulting from confinement due to COVID-19 and depressive symptomatology: a cross-sectional a population-based study. Compr Psychiatry. 2021;104:152214.

40. Ko C-H, Yen C-F, Yen J-Y, Yang M-J. Psychosocial impact among the public of the severe acute respiratory syndrome epidemic in Taiwan. Psychiatry Clin Neurosci. 2006;60:397-403.

41. Li TW, Lee TMC, Goodwin R, Ben-Ezra M, Liang L, Liu H, et al. Social capital, income loss, and psychobehavioral responses amid COVID-19: a population-based analysis. Int J Environ Res Public Health. 2020;17:8888.

42. Jalloh MF, Li WS, Bunnell RE, Ethier KA, O'Leary A, Hageman KM, et al. Impact of Ebola experiences and risk perceptions on mental health in Sierra Leone, July 2015. BMJ Glob Health. 2018;3:e000471.

43. Lau JTF, Yang X, Pang E, Tsui HY, Wong E, Wing YK. SARS-related perceptions in Hong Kong. Emerg Infect Dis. 2005;11:417-24.

44. Lau JT, Yang X, Tsui HY, Pang E, Wing YK. Positive mental health-related impacts of the SARS epidemic on the general public in Hong Kong and their associations with other negative impacts. J Infect. 2006;53:114-24.

45. Cenat JM, McIntee SE, Guerrier M, Derivois D, Rousseau C, Dalexis RD, et al. Psychological distress among adults from the urban and rural areas affected by the Ebola virus disease in the Democratic Republic of the Congo. Soc Psychiatry Psychiatr Epidemiol. 2021;56:57-62.

46. Chandola T, Kumari M, Booker CL, Benzeval M. The mental health impact of COVID-19 and lockdown-related stressors among adults in the UK. Psychol Med. 2020. https://doi.org/10.1017/S0033291720005048.

47. Daly M, Sutin A, Robinson E. Longitudinal changes in mental health and the COVID-19 pandemic: evidence from the UK Household Longitudinal Study. Psychol Med. 2020. https://doi.org/10.1017/S0033291720004432.

48. Harris SM, Sandal GM. COVID-19 and psychological distress in Norway: the role of trust in the healthcare system. Scand J Public Health. 2021;49:96-103.

49. Kämpfen F, Kohler IV, Ciancio A, Bruine de Bruin W, Maurer J, Kohler HP. Predictors of mental health during the Covid-19 pandemic in the US: role of economic concerns, health worries and social distancing. PLoS One. 2020;15:e0241895.

50. Li LZ, Wang S. Prevalence and predictors of general psychiatric disorders and loneliness during COVID-19 in the United Kingdom. Psychiatry Res. 2020;291:113267.

51. McGinty EE, Presskreischer R, Han H, Barry CL. Psychological distress and loneliness reported by US adults in 2018 and April 2020. JAMA. 2020;324:93-4.

52. McGinty EE, Presskreischer R, Anderson KE, Han H, Barry CL. Psychological distress and COVID-19-related stressors reported in a longitudinal cohort of US adults in April and July 2020. JAMA. 2020;324:2555-7.

53. Niedzwiedz CL, Green MJ, Benzeval M, Campbell D, Craig P, Demou E, et al. Mental health and health behaviours before and during the initial phase of the COVID-19 lockdown: longitudinal analyses of the UK Household Longitudinal Study. J Epidemiol Community Health. 2021;75:224-31.

54. Peng EY, Lee MB, Tsai ST, Yang CC, Morisky DE, Tsai LT, et al. Population-based post-crisis psychological distress: an example from the SARS outbreak in Taiwan. J Formos Med Assoc. 2010;109:524-32.

55. Pierce M, Hope H, Ford T, Hatch S, Hotopf M, John A, et al. Mental health before and during the COVID-19 pandemic: a longitudinal probability sample survey of the UK population. Lancet Psychiatry. 2020;7:883-92.

56. Riehm KE, Holingue C, Kalb LG, Bennett D, Kapteyn A, Jiang Q, et al. Associations between media exposure and mental distress among us adults at the beginning of the COVID-19 pandemic. Am J Prev Med. 2020;59:630-8.

57. Robinson E, Daly M. Explaining the rise and fall of psychological distress during the COVID-19 crisis in the United States: longitudinal evidence from the Understanding America Study. Br J Health Psychol. 2021;26:570-87.

58. Jackson SE, Garnett C, Shahab L, Oldham M, Brown J. Association of the COVID-19 lockdown with smoking, drinking and attempts to quit in England: an analysis of 2019-20 data. Addiction. 2021;116:1233-44.

59. Leske S, Kolves K, Crompton D, Arensman E, de Leo D. Real-time suicide mortality data from police reports in Queensland, Australia, during the COVID-19 pandemic: an interrupted time-series analysis. Lancet Psychiatry. 2021;8:58-63.

60. Qin P, Mehlum L. National observation of death by suicide in the first 3 months under COVID-19 pandemic. Acta Psychiatr Scand. 2021;143:92-3.

61. Wasserman IM. The impact of epidemic, war, prohibition and media on suicide: United States, 1910–1920. Suicide Life Threat Behav. 1992;22:240-54.

62. Li J, Yang Z, Qiu H, Wang Y, Jian L, Ji J, et al. Anxiety and depression among general population in China at the peak of the COVID-19 epidemic. World Psychiatry. 2020;19:249-50.

63. Elton-Marshall T, Wells S, Jankowicz D, Nigatu YT, Wickens CM, Rehm J, et al. Multiple COVID-19 risk factors increase the likelihood of experiencing anxiety symptoms in Canada. Can J Psychiatry. 2020;66:56-8.

64. Gao J, Zheng P, Jia Y, Chen H, Mao Y, Chen S, et al. Mental health problems and social media exposure during COVID-19 outbreak. PLoS One. 2020;15:e0231924.

65. Peretti-Watel P, Alleaume C, Leger D, Beck F, Verger P, Grp C. Anxiety, depression and sleep problems: a second wave of COVID-19. Gen Psychiatr. 2020;33:e100299.

66. Ni MY, Yang L, Leung CMC, Li N, Yao XI, Wang Y, et al. Mental health, risk factors, and social media use during the COVID-19 epidemic and cordon sanitaire among the community and health professionals in Wuhan, China: cross-sectional survey. JMIR Ment Health. 2020;7:e19009.

67. Benke C, Autenrieth LK, Asselmann E, Pane-Farre CA. Stay-at-home orders due to the COVID-19 pandemic are associated with elevated depression and anxiety in younger, but not older adults: results from a nationwide community sample of adults from Germany. Psychol Med. 2020. https://doi.org/10.1017/S0033291720003438.

68. Pouso S, Borja A, Fleming LE, Gomez-Baggethun E, White MP, Uyarra MC. Contact with blue-green spaces during the COVID-19 pandemic lockdown beneficial for mental health. Sci Total Environ. 2021;756:143984.

69. Jacques-Avino C, Lopez-Jimenez T, Medina-Perucha L, de Bont J, Goncalves AQ, Duarte-Salles T, et al. Gender-based approach on the social impact and mental health in Spain during COVID-19 lockdown: a cross-sectional study. BMJ Open. 2020;10:e044617.

70. Jia R, Ayling K, Chalder T, Massey A, Broadbent E, Coupland C, et al. Mental health in the UK during the COVID-19 pandemic: cross-sectional analyses from a community cohort study. BMJ Open. 2020;10:e040620.

71. Palgi Y, Shrira A, Ring L, Bodner E, Avidor S, Bergman Y, et al. The loneliness pandemic: loneliness and other concomitants of depression, anxiety and their comorbidity during the COVID-19 outbreak. J Affect Disord. 2020;275:109-11.

72. Makhashvili N, Javakhishvili JD, Sturua L, Pilauri K, Fuhr DC, Roberts B. The influence of concern about COVID-19 on mental health in the Republic of Georgia: a cross-sectional study. Global Health. 2020;16:111.

73. Barzilay R, Moore TM, Greenberg DM, DiDomenico GE, Brown LA, White LK, et al. Resilience, COVID-19-related stress, anxiety and depression during the pandemic in a large population enriched for healthcare providers. Transl Psychiatry. 2020;10:291.

74. Huang J, Liu F, Teng Z, Chen J, Zhao J, Wang X, et al. Public behavior change, perceptions, depression, and anxiety in relation to the COVID-19 outbreak. Open Forum Infect Dis. 2020;7:ofaa273.

75. Bressington DT, Cheung TCC, Lam SC, Suen LKP, Fong TKH, Ho HSW, et al. Association between depression, health beliefs, and face mask use during the COVID-19 pandemic. Front Psychiatry. 2020;11:571179.

76. Skapinakis P, Bellos S, Oikonomou A, Dimitriadis G, Gkikas P, Perdikari E, et al. Depression and its relationship with coping strategies and illness perceptions during the COVID-19 lockdown in Greece: a cross-sectional survey of the population. Depress Res Treat. 2020;2020:3158954.

77. Bryan CJ, Bryan AO, Baker JC. Associations among state-level physical distancing measures and suicidal thoughts and behaviors among U.S. adults during the early COVID-19 pandemic. Suicide Life Threat Behav. 2020;26:e12653.

78. Iob E, Frank P, Steptoe A, Fancourt D. Levels of severity of depressive symptoms among at-risk groups in the UK during the COVID-19 pandemic. JAMA Netw Open. 2020;3:e2026064.

79. Wang S, Zhang Y, Ding W, Meng Y, Hu H, Liu Z, et al. Psychological distress and sleep problems when people are under interpersonal isolation during an epidemic: a nationwide multicenter crosssectional study. Eur Psychiatry. 2020;63:e77.

80. Zhang Y, Wang S, Ding W, Meng Y, Hu HT, Liu ZH, et al. Status and influential factors of anxiety depression and insomnia symptoms in the work resumption period of COVID-19 epidemic: a multicenter cross-sectional study. J Psychosom Res. 2020;138:110253.

81. Mohammadi MR, Zarafshan H, Bashi SK, Mohammadi F, Khaleghi A. The role of public trust and media in the psychological and behavioral responses to the COVID-19 pandemic. Iran J Psychiatry. 2020;15:189-204.

82. Forte G, Favieri F, Tambelli R, Casagrande M. The enemy which sealed the world: effects of COVID-19 diffusion on the psychological state of the Italian population. J Clin Med. 2020;9:1802.

83. Fekih-Romdhane F, Ghrissi F, Abbassi B, Cherif W, Cheour M. Prevalence and predictors of PTSD during the COVID-19 pandemic: findings from a Tunisian community sample. Psychiatry Res. 2020;290:113131.

84. Kolacz J, Dale LP, Nix EJ, Roath OK, Lewis GF, Porges SW. Adversity history predicts self-reported autonomic reactivity and mental health in US residents during the COVID-19 pandemic. Front Psychiatry. 2020;11:577728.

85. Alshehri FS, Alatawi Y, Alghamdi BS, Alhifany AA, Alharbi A. Prevalence of post-traumatic stress disorder during the COVID-19 pandemic in Saudi Arabia. Saudi Pharm J. 2020;28:1666-73.

86. Gómez-Salgado J, Andrés-Villas M, Domínguez-Salas S, Díaz-Milanés D, Ruiz-Frutos C. Related health factors of psychological distress during the COVID-19 pandemic in Spain. Int J Environ Res Public Health. 2020;17:3947.

87. Dominguez-Salas S, Gomez-Salgado J, Andres-Villas M, Diaz-Milanes D, Romero-Martin M, Ruiz-Frutos C. Psycho-emotional approach to the psychological distress related to the COVID-19 pandemic in Spain: a cross-sectional observational study. Healthcare. 2020;8:190.

88. Duarte MQ, Santo M, Lima CP, Giordani JP, Trentini CM. Covid-19 and the impacts on mental health: a sample from Rio Grande do Sul, Brazil. Cien Saude Colet. 2020;25:3401-11.

89. Shevlin M, McBride O, Murphy J, Miller JG, Hartman TK, Levita L, et al. Anxiety, depression, traumatic stress and COVID-19-related anxiety in the UK general population during the COVID-19 pandemic. BJPsych Open. 2020;6:e125.

90. Zhu S, Wu Y, Zhu CY, Hong WC, Yu ZX, Chen ZK, et al. The immediate mental health impacts of the COVID-19 pandemic among people with or without quarantine managements. Brain Behav Immun. 2020;87:56-8.

91. Guo Y, Cheng C, Zeng Y, Li YR, Zhu MT, Yang WX, et al. Mental health disorders and associated risk factors in quarantined adults during the COVID-19 outbreak in China: cross-sectional study. J Med Internet Res. 2020;22:e20328.

92. Ben-Ezra M, Cary N, Goodwin R. The association between COVID-19 WHO non-recommended behaviors with psychological distress in the UK population: a preliminary study. J Psychiatr Res. 2020;130:286-8.

93. Ben-Ezra M, Sun S, Hou WK, Goodwin R. The association of being in quarantine and related COVID-19 recommended and non-recommended behaviors with psychological distress in Chinese population. J Affect Disord. 2020;275:66-8.

94. Shi L, Lu Z-A, Que J-Y, Huang X-L, Liu L, Ran M-S, et al. Prevalence of and risk factors associated with mental health symptoms among the general population in China during the coronavirus disease 2019 pandemic. JAMA Netw Open. 2020;3:e2014053.
95. Munk AJL, Schmidt NM, Alexander N, Henkel K, Hennig J. COVID-19-beyond virology: potentials for maintaining mental health during lockdown. PLoS One. 2020;15:e0236688.
96. O'Connor RC, Wetherall K, Cleare S, McClelland H, Melson AJ, Niedzwiedz CL, et al. Mental health and wellbeing during the COVID-19 pandemic: longitudinal analyses of adults in the UK COVID-19 Mental Health & Wellbeing study. Br J Psychiatry. 2021;218:326-33.
97. Caballero-Dominguez CC, Jimenez-Villamizar MP, Campo-Arias A. Suicide risk during the lockdown due to coronavirus disease (COVID-19) in Colombia. Death Stud. 2022;46:885-90.
98. Winkler P, Formanek T, Mlada K, Kagstrom A, Mohrova Z, Mohr P, et al. Increase in prevalence of current mental disorders in the context of COVID-19: analysis of repeated nationwide cross-sectional surveys. Epidemiol Psychiatr Sci. 2020;29:e173.
99. Galea S, Tracy M. Participation rates in epidemiologic studies. Ann Epidemiol. 2007;17:643-53.
100. Goldmann E, Galea S. Mental health consequences of disasters. Annu Rev Public Health. 2014;35:169-83.
101. Charlson F, van Ommeren M, Flaxman A, Cornett J, Whiteford H, Saxena S. New WHO prevalence estimates of mental disorders in conflict settings: a systematic review and meta-analysis. Lancet. 2019;394:240-8.
102. Cénat JM, Felix N, Blais-Rochette C, Rousseau C, Bukaka J, Derivois D, et al. Prevalence of mental health problems in populations affected by the Ebola virus disease: a systematic review and meta-analysis. Psychiatry Res. 2020;289:113033.
103. Santomauro DF, Mantilla Herrera AM, Shadid J, Zheng P, Ashbaugh C, Pigott DM, et al. Global prevalence and burden of depressive and anxiety disorders in 204 countries and territories in 2020 due to the COVID-19 pandemic. Lancet. 2021;398:1700-12.
104. Pierce M, McManus S, Hope H, Hotopf M, Ford T, Hatch SL, et al. Mental health responses to the COVID-19 pandemic: a latent class trajectory analysis using longitudinal UK data. Lancet Psychiatry. 2021;8:610-9.

105. Shevlin M, Butter S, McBride O, Murphy J, Gibson-Miller J, Hartman TK, et al. Refuting the myth of a 'tsunami' of mental ill-health in populations affected by COVID-19: evidence that response to the pandemic is heterogeneous, not homogeneous. Psychol Med. 2021. https://doi.org/10.1017/s0033291721001665.

106. Shanafelt T, Ripp J, Trockel M. Understanding and addressing sources of anxiety among health care professionals during the COVID-19 pandemic. JAMA. 2020;323:2133-4.

107. The Lancet. The truth is out there, somewhere. Lancet. 2020;396:291.

108. FT Visual & Data Journalism Team, Financial TImes. Exiting lockdowns: tracking governments' changing coronavirus responses. 2020. https://ig.ft.com/coronavirus-lockdowns/. Accessed 1 September 2020.

109. Ni MY, Li TK, Pang H, Chan BHY, Kawachi I, Viswanath K, et al. Longitudinal patterns and predictors of depression trajectories related to the 2014 Occupy Central/Umbrella Movement in Hong Kong. Am J Public Health. 2017;107:593-600.

110. Ni MY, Yao XI, Leung KSM, Yau C, Leung CMC, Lun P, et al. Depression and post-traumatic stress during major social unrest in Hong Kong: a 10-year prospective cohort study. Lancet. 2020;395:273-84.

111. Ni MY, Li TK, Pang H, Chan BHY, Yuan BY, Kawachi I, et al. Direct participation in and indirect exposure to the Occupy Central Movement and depressive symptoms: a longitudinal study of Hong Kong adults. Am J Epidemiol. 2016;184:636-43.

112. Kramer ADI, Guillory JE, Hancock JT. Experimental evidence of massive-scale emotional contagion through social networks. Proc Natl Acad Sci U S A. 2014;111:8788-90.

113. Holman EA, Thompson RR, Garfin DR, Silver RC. The unfolding COVID-19 pandemic: a probability-based, nationally representative study of mental health in the United States. Sci Adv. 2020;6:eabd5390.

114. World Health Organization. Mental health and psychosocial considerations during the COVID-19 outbreak. 2020. https://www.who.int/docs/default-source/coronaviruse/mental-health-considerations.pdf?sfvrsn=6d3578af_2. Accessed 30 August 2020.

115. Czeisler MÉ, Lane RI, Petrosky E, Wiley JF, Christensen A, Njai R, et al. Mental health, substance use, and suicidal ideation during the COVID-19 pandemic - United States, June 24-30, 2020. MMWR Morb Mortal Wkly Rep. 2020;69:1049-57.

116. Baumann C, Rousseau H, Tarquinio C, Batt M, Tarquinio P, Lebreuilly R, et al. Effect of the COVID-19 outbreak and lockdown on mental health among post-secondary students in the Grand Est region of France: results of the PIMS-CoV19 study. Health Qual Life Outcomes. 2021;19:265.

117. Moreno C, Wykes T, Galderisi S, Nordentoft M, Crossley N, Jones N, et al. How mental health care should change as a consequence of the COVID-19 pandemic. Lancet Psychiatry. 2020;7:813-24.

118. Zürcher SJ, Kerksieck P, Adamus C, Burr CM, Lehmann AI, Huber FK, et al. Prevalence of mental health problems during virus epidemics in the general public, health care workers and survivors: a rapid review of the evidence. Front Public Health. 2020;8:560389.

119. Norris FH, Tracy M, Galea S. Looking for resilience: understanding the longitudinal trajectories of responses to stress. Soc Sci Med. 2009;68:2190-8.

120. Van Emmerik AAP, Kamphuis JH, Hulsbosch AM, Emmelkamp PMG. Single session debriefing after psychological trauma: a meta-analysis. Lancet. 2002;360:766-71.

121. Galea S, Merchant RM, Lurie N. The mental health consequences of COVID-19 and physical distancing: the need for prevention and early intervention. JAMA Intern Med. 2020;180:817-8.

122. Fu Z, Burger H, Arjadi R, Bockting CLH. Effectiveness of digital psychological interventions for mental health problems in low-income and middle-income countries: a systematic review and meta-analysis. Lancet Psychiatry. **2020**;7:851-64.

123. Galea S. Compassion in a time of COVID-19. Lancet. 2020;395:1897-8.

124. Kamara S, Walder A, Duncan J, Kabbedijk A, Hughes P, Muana A. Mental health care during the Ebola virus disease outbreak in Sierra Leone. Bull World Health Organ. 2017;95:842-7.

125. The Lancet Planetary Health. Post-COVID-19 spending. Lancet Planet Health. 2020;4:e168.

126. Du J, Fan N, Zhao M, Hao W, Liu T, Lu L, et al. Expert consensus on the prevention and treatment of substance use and addictive behaviour-related disorders during the COVID-19 pandemic. Gen Psychiatr. 2020;33:e100252.

127. Chandan JS, Taylor J, Bradbury-Jones C, Nirantharakumar K, Kane E, Bandyopadhyay S. COVID-19: a public health approach to manage domestic violence is needed. Lancet Public Health. 2020;5:e309.

128. Leung GM, Cowling BJ, Wu JT. From a sprint to a marathon in Hong Kong. N Engl J Med. 2020;382:e45.

129. Ni MY, Yao XI, Cheung F, Wu JT, Schooling CM, Pang H, et al. Determinants of physical, mental and social well-being: a longitudinal environment-wide association study. Int J Epidemiol. 2020;49:380-9.

130. Wu JT, Leung K, Lam TTY, Ni MY, Wong CKH, Peiris JSM, et al. Nowcasting epidemics of novel pathogens: lessons from COVID-19. Nat Med. 2021;27:388-95.

131. Phillips MR. Perspectives: World Mental Health Day 2020: promoting global mental health during COVID-19. China CDC Weekly. 2020;2:844-7.

132. Van Bavel JJ, Baicker K, Boggio PS, Capraro V, Cichocka A, Cikara M, et al. Using social and behavioural science to support COVID-19 pandemic response. Nat Hum Behav. 2020;4:460-71.

133. Lau LS, Samari G, Moresky RT, Casey SE, Kachur SP, Roberts LF, et al. COVID-19 in humanitarian settings and lessons learned from past epidemics. Nat Med. 2020;26:647-8.

134. Cheung CK, Tse JW. Institutional trust as a determinant of anxiety during the SARS crisis in Hong Kong. Soc Work Public Health. 2008;23:41-54.

135. Singer M, Bulled N, Ostrach B, Mendenhall E. Syndemics and the biosocial conception of health. Lancet. 2017;389:941-50.

136. Ni MY, Leung CMC, Leung GM. The epidemiology of population mental wellbeing in China. Lancet Public Health. 2020;5:e631-2.

137. Kola L. Global mental health and COVID-19. Lancet Psychiatry. 2020;7:655-7.

138. North CS. Current research and recent breakthroughs on the mental health effects of disasters. Curr Psychiatry Rep. 2014;16:481.

139. Neria Y, Nandi A, Galea S. Post-traumatic stress disorder following disasters: a systematic review. Psychol Med. 2008;38:467-80.

140. Patel V, Saxena S, Lund C, Thornicroft G, Baingana F, Bolton P, et al. The Lancet Commission on global mental health and sustainable development. Lancet. 2018;392:1553-98.

141. Fauci AS, Morens DM. The perpetual challenge of infectious diseases. N Engl J Med. 2012;366:454-61.

142. Scudellari M. How the pandemic might play out in 2021 and beyond. Nature. 2020;584:22-5.
143. Adhanom Ghebreyesus T. Addressing mental health needs: an integral part of COVID-19 response. World Psychiatry. 2020;19:129-30.
144. Wang YN, Di Y, Ye JJ, Wei WB. Study on the public psychological states and its related factors during the outbreak of coronavirus disease 2019 (COVID-19) in some regions of China. Psychol Health Med. 2021;26:13-22.
145. Naser AY, Dahmash EZ, Al-Rousan R, Alwafi H, Alrawashdeh HM, Ghoul I, et al. Mental health status of the general population, healthcare professionals, and university students during 2019 coronavirus disease outbreak in Jordan: a cross-sectional study. Brain Behav. 2020;10:e01730.
146. Smith L, Jacob L, Yakkundi A, McDermott D, Armstrong NC, Barnett Y, et al. Correlates of symptoms of anxiety and depression and mental wellbeing associated with COVID-19: a cross-sectional study of UK-based respondents. Psychiatry Res. 2020;291:113138.
147. Badellino H, Gobbo ME, Torres E, Aschieri ME. Early indicators and risk factors associated with mental health problems during COVID-19 quarantine: is there a relationship with the number of confirmed cases and deaths? Int J Soc Psychiatry. 2021;67:567-75.
148. Burhamah W, AlKhayyat A, Oroszlanyova M, AlKenane A, Almansouri A, Behbehani M, et al. The psychological burden of the COVID-19 pandemic and associated lockdown measures: experience from 4000 participants. J Affect Disord. 2020;277:977-85.
149. Casagrande M, Favieri F, Tambelli R, Forte G. The enemy who sealed the world: effects quarantine due to the COVID-19 on sleep quality, anxiety, and psychological distress in the Italian population. Sleep Med. 2020;75:12-20.
150. Fu WN, Wang C, Zou L, Guo YY, Lu ZX, Yan SJ, et al. Psychological health, sleep quality, and coping styles to stress facing the COVID-19 in Wuhan, China. Transl Psychiatry. 2020;10:225.
151. Gualano MR, Lo Moro G, Voglino G, Bert F, Siliquini R. Effects of COVID-19 lockdown on mental health and sleep disturbances in Italy. Int J Environ Res Public Health. 2020;17:4779.
152. Hammarberg K, Tran T, Kirkman M, Fisher J. Sex and age differences in clinically significant symptoms of depression and anxiety among people in Australia in the first month of COVID-19 restrictions: a national survey. BMJ Open. 2020;10:e042696.

153. Islam MS, Ferdous MZ, Potenza MN. Panic and generalized anxiety during the COVID-19 pandemic among Bangladeshi people: an online pilot survey early in the outbreak. J Affect Disord. 2020;276:30-7.

154. Martinez EZ, Silva FM, Morigi TZ, Zucoloto ML, Silva TL, Joaquim AG, et al. Physical activity in periods of social distancing due to COVID-19: a cross-sectional survey. Cien Saude Colet. 2020;25:4157-68.

155. Mollaioli D, Sansone A, Ciocca G, Limoncin E, Colonnello E, Di Lorenzo G, et al. Benefits of sexual activity on psychological, relational, and sexual health during the COVID-19 breakout. J Sex Med. 2021;18:35-49.

156. Rossi R, Socci V, Talevi D, Mensi S, Niolu C, Pacitti F, et al. COVID-19 pandemic and lockdown measures impact on mental health among the general population in Italy. Front Psychiatry. 2020;11:790.

157. Thomas J, Barbato M, Verlinden M, Gaspar C, Moussa M, Ghorayeb J, et al. Psychosocial correlates of depression and anxiety in the United Arab Emirates during the COVID-19 pandemic. Front Psychiatry. 2020;11:564172.

158. Janati Idrissi A, Lamkaddem A, Benouajjit A, Ben El Bouaazzaoui M, El Houari F, Alami M, et al. Sleep quality and mental health in the context of COVID-19 pandemic and lockdown in Morocco. Sleep Med. 2020;74:248-53.

159. Shatla MM, Khafagy AA, Bulkhi AA, Aljahdali IA. Public concerns and mental health changes related to the COVID-19 pandemic lockdown in Saudi Arabia. Clin Lab. 2020;66:2125-32.

160. Ueda M, Stickley A, Sueki H, Matsubayashi T. Mental health status of the general population in Japan during the COVID-19 pandemic. Psychiatry Clin Neurosci. 2020;74:505-6.

161. Zhao HY, He XY, Fan GH, Li LP, Huang QJ, Qiu QM, et al. COVID-19 infection outbreak increases anxiety level of general public in China: involved mechanisms and influencing factors. J Affect Disord. 2020;276:446-52.

162. Sherman AC, Williams ML, Amick BC, Hudson TJ, Messias EL. Mental health outcomes associated with the COVID-19 pandemic: prevalence and risk factors in a southern US state. Psychiatry Res. 2020;293:113476.

163. Hossain MT, Ahammed B, Chanda SK, Jahan N, Ela MZ, Islam MN. Social and electronic media exposure and generalized anxiety disorder among people during COVID-19 outbreak in Bangladesh: a preliminary observation. PLoS One. 2020;15:e0238974.

164. Ni Z, Lebowitz ER, Zou Z, Wang H, Liu H, Shrestha R, et al. Response to the COVID-19 outbreak in urban settings in China. J Urban Health. 2021;98:41-52.

165. Fisher JR, Tran TD, Hammarberg K, Sastry J, Nguyen H, Rowe H, et al. Mental health of people in Australia in the first month of COVID-19 restrictions: a national survey. Med J Aust. 2020;213:458-64.

166. Zhong BL, Zhou DY, He MF, Li Y, Li WT, Ng CH, et al. Mental health problems, needs, and service use among people living within and outside Wuhan during the COVID-19 epidemic in China. Ann Transl Med. 2020;8:1392.

167. Lu P, Li X, Lu L, Zhang Y. The psychological states of people after Wuhan eased the lockdown. PLoS One. 2020;15:e0241173.

168. Huang Y, Zhao N. Generalized anxiety disorder, depressive symptoms and sleep quality during COVID-19 outbreak in China: a web-based cross-sectional survey. Psychiatry Res. 2020;288:112954.

169. Sljivo A, Kacamakovic M, Quraishi I, Kulenovic AD. Fear and depression among residents of Bosnia and Herzegovina during covid-19 outbreak - internet survey. Psychiatria Danubina. 2020;32:266-72.

170. Tang F, Liang J, Zhang H, Kelifa MM, He Q, Wang P. COVID-19 related depression and anxiety among quarantined respondents. Psychol Health. 2021;36:164-78.

171. Ren Z, Zhou Y, Liu Y. The psychological burden experienced by Chinese citizens during the COVID-19 outbreak: prevalence and determinants. BMC Public Health. 2020;20:1617.

172. Guo J, Feng XL, Wang XH, van IMH. Coping with COVID-19: exposure to COVID-19 and negative impact on livelihood predict elevated mental health problems in Chinese adults. Int J Environ Res Public Health. 2020;17:3857.

173. Lee Y, Yang BX, Liu Q, Luo D, Kang L, Yang F, et al. Synergistic effect of social media use and psychological distress on depression in china during the COVID-19 epidemic. Psychiatry Clin Neurosci. 2020;74:552-4.

174. Zhang WY, Yang XT, Zhao JF, Yang FZ, Jia YJ, Cui C, et al. Depression and psychological-behavioral responses among the general public in China during the early stages of the COVID-19 pandemic: survey study. J Med Internet Res. 2020;22:e22227.

175. Castelli L, Di Tella M, Benfante A, Romeo A. The spread of COVID-19 in the Italian population: anxiety, depression, and post-traumatic stress symptoms. Can J Psychiatry. 2020;65:731-2.

176. Campos J, Martins BG, Campos LA, Maroco J, Saadiq RA, Ruano R. Early psychological impact of the COVID-19 pandemic in brazil: a national survey. J Clin Med. 2020;9:2976.

177. Ramirez LPG, Arriaga RJM, Hernandez-Gonzalez MA, De la Roca-Chiapas JM. Psychological distress and signs of post-traumatic stress in response to the COVID-19 health emergency in a Mexican sample. Psychol Res Behav Manag. 2020;13:589-97.

178. Karatzias T, Shevlin M, Murphy J, McBride O, Ben-Ezra M, Bentall RP, et al. Posttraumatic stress symptoms and associated comorbidity during the COVID-19 pandemic in Ireland: a population-based study. J Trauma Stress. 2020;33:365-70.

179. Costantini A, Mazzotti E. Italian validation of CoViD-19 Peritraumatic Distress Index and preliminary data in a sample of general population. Rivista Di Psichiatria. 2020;55:145-51.

180. Al Sinawi H, Al Balushi N, Al-Mahrouqi T, Al Ghailani A, McCall RK, Sultan A, et al. Predictors of psychological distress among the public in Oman amid coronavirus disease 2019 pandemic: a cross-sectional analytical study. Psychol Health Med. 2021;26:131-44.

181. Hyland P, Shevlin M, McBride O, Murphy J, Karatzias T, Bentall RP, et al. Anxiety and depression in the Republic of Ireland during the COVID-19 pandemic. Acta Psychiatr Scand. 2020;142:249-56.

182. Rahman MA, Hoque N, Alif SM, Salehin M, Islam SMS, Banik B, et al. Factors associated with psychological distress, fear and coping strategies during the COVID-19 pandemic in Australia. Global Health. 2020;16:95.

183. Every-Palmer S, Jenkins M, Gendall P, Hoek J, Beaglehole B, Bell C, et al. Psychological distress, anxiety, family violence, suicidality, and wellbeing in New Zealand during the COVID-19 lockdown: a cross-sectional study. PLoS One. 2020;15:e0241658.

184. Goodwin R, Hou WK, Sun S, Ben-Ezra M. Psychological and behavioural responses to COVID-19: a China-Britain comparison. J Epidemiol Community Health. 2021;75:189-92.

185. Kikuchi H, Machida M, Nakamura I, Saito R, Odagiri Y, Kojima T, et al. Changes in psychological distress during the COVID-19 pandemic in Japan: a longitudinal study. J Epidemiol. 2020;30:522-8.

186. American Psychiatric Association. Trauma- and stressor-related disorders. *Diagnostic and statistical manual of mental disorders, fifth edition*. American Psychiatric Association: Arlington, 2013, pp 265-90.

187. Pfefferbaum B, North CS. Mental health and the COVID-19 pandemic. N Engl J Med. 2020;383:510-2.

188. World Health Organization. Case definition recommendations for Ebola or Marburg virus diseases. 2014. https://apps.who.int/iris/bitstream/handle/10665/146397/WHO_EVD_CaseDef_14%C2%B71_eng.pdf?sequence=1. Accessed 30 August 2020.

189. Fydrich T, Dowdall D, Chambless DL. Reliability and validity of the beck anxiety inventory. J Anxiety Disord. 1992;6:55-61.

190. Kroenke K, Spitzer RL, Williams JB, Monahan PO, Löwe B. Anxiety disorders in primary care: prevalence, impairment, comorbidity, and detection. Ann Intern Med. 2007;146:317-25.

191. Spitzer RL, Kroenke K, Williams JB, Löwe B. A brief measure for assessing generalized anxiety disorder: the GAD-7. Arch Intern Med. 2006;166:1092-7.

192. Hamilton M. The assessment of anxiety states by rating. Br J Med Psychol. 1959;32:50-5.

193. Zigmond AS, Snaith RP. The Hospital Anxiety and Depression Scale. Acta Psychiatr Scand. 1983;67:361-70.

194. Sheehan DV, Lecrubier Y, Sheehan KH, Amorim P, Janavs J, Weiller E, et al. The Mini-International Neuropsychiatric Interview (M.I.N.I.): the development and validation of a structured diagnostic psychiatric interview for DSM-IV and ICD-10. J Clin Psychiatry. 1998;59 Suppl 20:22-33.

195. American Psychiatric Association. LEVEL 2, Anxiety, Adult(PROMIS Emotional Distress, Anxiety, Short Form). 2020. https://www.psychiatry.org/psychiatrists/practice/dsm/educational-resources/assessment-measures. Accessed 6 February 2021.

196. Zung WWK. A rating instrument for anxiety disorders. Psychosomatics. 1971;12:371-9.

197. Beck AT, Ward CH, Mendelson M, Mock J, Erbaugh J. An inventory for measuring depression. Arch Gen Psychiatry. 1961;4:561-71.

198. Dozois DJA, Dobson KS, Ahnberg JL. A psychometric evaluation of the Beck Depression Inventory–II. Psychol Assess. 1998;10:83-9.

199. Radloff LS. The CES-D Scale: a self-report depression scale for research in the general population. Appl Psychol Meas. 1977;1:385-401.

200. World Health Organization. Wellbeing measures in primary health care - the DepCare Project. 1998. https://www.euro.who.int/__data/assets/pdf_file/0016/130750/E60246.pdf. Accessed 1 September 2020.

201. Kroenke K, Spitzer RL, Williams JB. The Patient Health Questionnaire-2: validity of a two-item depression screener. Med Care. 2003;41:1284-92.

202. Kroenke K, Strine TW, Spitzer RL, Williams JB, Berry JT, Mokdad AH. The PHQ-8 as a measure of current depression in the general population. J Affect Disord. 2009;114:163-73.

203. Kroenke K, Spitzer RL, Williams JB. The PHQ-9: validity of a brief depression severity measure. J Gen Intern Med. 2001;16:606-13.

204. Lee Y, Yang MJ, Lai TJ, Chiu NM, Chau TT. Development of the Taiwanese Depression Questionnaire. Chang Gung Med J. 2000;23:688-94.

205. Zung WW. A self-rating depression scale. Arch Gen Psychiatry. 1965;12:63-70.

206. Cloitre M, Shevlin M, Brewin CR, Bisson JI, Roberts NP, Maercker A, et al. The International Trauma Questionnaire: development of a self-report measure of ICD-11 PTSD and complex PTSD. Acta Psychiatr Scand. 2018;138:536-46.

207. Prins A, Bovin MJ, Smolenski DJ, Marx BP, Kimerling R, Jenkins-Guarnieri MA, et al. The Primary Care PTSD Screen for DSM-5 (PC-PTSD-5): Development and evaluation within a veteran primary care sample. J Gen Intern Med. 2016;31:1206-11.

208. Weiss DS, Marmar CR. The Impact of Event Scale - Revised. In: Wilson, J and Keane, TM (ed). *Assessing psychological trauma and PTSD*. Guilford: New York, 1996, pp 399-411.

209. Blanchard EB, Jones-Alexander J, Buckley TC, Forneris CA. Psychometric properties of the PTSD Checklist (PCL). Behav Res Ther. 1996;34:669-73.

210. Blevins CA, Weathers FW, Davis MT, Witte TK, Domino JL. The Posttraumatic Stress Disorder Checklist for DSM-5 (PCL-5): development and initial psychometric evaluation. J Trauma Stress. 2015;28:489-98.

211. Derogatis LR, *BSI 18, Brief Symptom Inventory 18: Administration, Scoring and Procedures Manual*. NCS Pearson, Incorporated: Minneapolis, 2001.

212. Derogatis LR, Melisaratos N. The brief symptom inventory: an introductory report. Psychol Med. 1983;13:595-605.

213. Qiu JY, Shen B, Zhao M, Wang Z, Xie B, Xu YF. A nationwide survey of psychological distress among Chinese people in the COVID-19 epidemic: implications and policy recommendations. Gen Psychiatr. 2020;33:e100213.

214. Lee MB, Liao SC, Lee YJ, Wu CH, Tseng MC, Gau SF, et al. Development and verification of validity and reliability of a short screening instrument to identify psychiatric morbidity. J Formos Med Assoc. 2003;102:687-94.

215. Goldberg DP, *The Detection of Psychiatric Illness by Questionnaire: A Technique for the Identification and Assessment of Non-Psychotic Psychiatric Illness*. Oxford University Press: Oxford, 1972.

216. Goldberg DP, Hillier VF. A scaled version of the General Health Questionnaire. Psychol Med. 1979;9:139-45.

217. Strand BH, Dalgard OS, Tambs K, Rognerud M. Measuring the mental health status of the Norwegian population: a comparison of the instruments SCL-25, SCL-10, SCL-5 and MHI-5 (SF-36). Nord J Psychiatry. 2003;57:113-8.

218. Kessler RC, Andrews G, Colpe LJ, Hiripi E, Mroczek DK, Normand SL, et al. Short screening scales to monitor population prevalences and trends in non-specific psychological distress. Psychol Med. 2002;32:959-76.

219. Kroenke K, Spitzer RL, Williams JB, Löwe B. An ultra-brief screening scale for anxiety and depression: the PHQ-4. Psychosomatics. 2009;50:613-21.

220. Derogatis LR, Lipman RS, Covi L. SCL-90: an outpatient psychiatric rating scale - preliminary report. Psychopharmacol Bull. 1973;9:13-28.

221. Beusenberg M, Orley JH, World Health Organization. Division of Mental Health. A user's guide to the self reporting questionnaire (SRQ). 1994.

https://apps.who.int/iris/bitstream/handle/10665/61113/WHO_MNH_PSF_94.8.pdf?sequence=1&isAllowed=y. Accessed 1 September 2020.

222. Bryant RA, Moulds ML, Guthrie RM. Acute Stress Disorder Scale: a self-report measure of acute stress disorder. Psychol Assess. 2000;12:61-8.

223. Reinert DF, Allen JP. The alcohol use disorders identification test: an update of research findings. Alcohol Clin Exp Res. 2007;31:185-99.

224. Foa EB, Huppert JD, Leiberg S, Langner R, Kichic R, Hajcak G, et al. The Obsessive-Compulsive Inventory: development and validation of a short version. Psychol Assess. 2002;14:485-96.

225. Cheung YB, Liu KY, Yip PSF. Performance of the CES-D and its short forms in screening suicidality and hopelessness in the community. Suicide Life Threat Behav. 2007;37:79-88.

226. von Glischinski M, Teismann T, Prinz S, Gebauer JE, Hirschfeld G. Depressive Symptom Inventory Suicidality Subscale: optimal cut points for clinical and non-clinical samples. Clin Psychol Psychother. 2016;23:543-9.

227. Osman A, Bagge CL, Gutierrez PM, Konick LC, Kopper BA, Barrios FX. The Suicidal Behaviors Questionnaire-Revised (SBQ-R): validation with clinical and nonclinical samples. Assessment. 2001;8:443-54.

228. van Spijker BAJ, Batterham PJ, Calear AL, Farrer L, Christensen H, Reynolds J, et al. The Suicidal Ideation Attributes Scale (SIDAS): Community-based validation study of a new scale for the measurement of suicidal ideation. Suicide Life Threat Behav. 2014;44:408-19.

229. Nock MK, Holmberg EB, Photos VI, Michel BD. Self-Injurious Thoughts and Behaviors Interview: development, reliability, and validity in an adolescent sample. Psychol Assess. 2007;19:309-17.

230. Dawel A, Shou YY, Smithson M, Cherbuin N, Banfield M, Calear AL, et al. The effect of COVID-19 on mental health and wellbeing in a representative sample of Australian adults. Front Psychiatry. 2020;11:579985.

231. Bendau A, Plag J, Kunas S, Wyka S, Strohle A, Petzold MB. Longitudinal changes in anxiety and psychological distress, and associated risk and protective factors during the first three months of the COVID-19 pandemic in Germany. Brain Behav. 2021;11:e01964.

232. Gopal A, Sharma AJ, Subramanyam MA. Dynamics of psychological responses to COVID-19 in India: a longitudinal study. PLoS One. 2020;15:e0240650.

233. Abba-Aji A, Li DI, Hrabok M, Shalaby R, Gusnowski A, Vuong W, et al. COVID-19 pandemic and mental health: prevalence and correlates of new-onset obsessive-compulsive symptoms in a Canadian province. Int J Environ Res Public Health. 2020;17:6986.

234. Pieh C, Budimir S, Probst T. The effect of age, gender, income, work, and physical activity on mental health during coronavirus disease (COVID-19) lockdown in Austria. J Psychosom Res. 2020;136:110186.

235. Pieh C, T OR, Budimir S, Probst T. Relationship quality and mental health during COVID-19 lockdown. PLoS One. 2020;15:e0238906.

236. Nwachukwu I, Nkire N, Shalaby R, Hrabok M, Vuong W, Gusnowski A, et al. COVID-19 pandemic: age-related differences in measures of stress, anxiety and depression in Canada. Int J Environ Res Public Health. 2020;17:6366.

237. Ahmed MZ, Ahmed O, Aibao Z, Hanbin S, Siyu L, Ahmad A. Epidemic of COVID-19 in China and associated psychological problems. Asian J Psychiatr. 2020;51:102092.

238. Hou F, Bi F, Jiao R, Luo D, Song K. Gender differences of depression and anxiety among social media users during the COVID-19 outbreak in China:a cross-sectional study. BMC Public Health. 2020;20:1648.

239. Zhang W-R, Wang K, Yin L, Zhao W-F, Xue Q, Peng M, et al. Mental health and psychosocial problems of medical health workers during the COVID-19 epidemic in China. Psychother Psychosom. 2020;89:242-50.

240. Li S, Ye Z, Du C, Wei Q, He C. The residents' mental health status and community's role during the COVID-19 pandemic: a community-based cross-sectional study in China. Ann Transl Med. 2020;8:1321.

241. Lin LY, Wang J, Ou-yang XY, Miao Q, Chen R, Liang FX, et al. The immediate impact of the 2019 novel coronavirus (COVID-19) outbreak on subjective sleep status. Sleep Med. 2021;77:348-54.

242. Zhang J, Lu H, Zeng H, Zhang S, Du Q, Jiang T, et al. The differential psychological distress of populations affected by the COVID-19 pandemic. Brain Behav Immun. 2020;87:49-50.

243. Ren Y, Zhou Y, Qian W, Li Z, Liu Z, Wang R, et al. Letter to the Editor "A longitudinal study on the mental health of general population during the COVID-19 epidemic in China". Brain Behav Immun. 2020;87:132-3.

244. Liang YM, Wu KK, Zhou YJ, Huang X, Zhou YY, Liu ZK. Mental health in frontline medical workers during the 2019 novel coronavirus disease epidemic in China: a comparison with the general population. Int J Environ Res Public Health. 2020;17:6550.

245. Su JH, Chen XY, Yang N, Sun M, Zhou L. Proximity to people with COVID-19 and anxiety among community residents during the epidemic in Guangzhou, China. BJPsych Open. 2020;6:e75.

246. Wu MD, Han HQ, Lin TK, Chen M, Wu J, Du XF, et al. Prevalence and risk factors of mental distress in China during the outbreak of COVID-19: a national cross-sectional survey. Brain Behav. 2020;10:e01818.

247. Liu M, Zhang H, Huang H. Media exposure to COVID-19 information, risk perception, social and geographical proximity, and self-rated anxiety in China. BMC Public Health. 2020;20:1649.

248. Lei L, Huang X, Zhang S, Yang J, Yang L, Xu M. Comparison of prevalence and associated factors of anxiety and depression among people affected by versus people unaffected by quarantine during the COVID-19 epidemic in southwestern China. Med Sci Monit. 2020;26:e924609.

249. Zhao YQ, An YY, Tan X, Li XH. Mental health and its influencing factors among self-isolating ordinary citizens during the beginning epidemic of COVID-19. J Loss Trauma. 2020;25:580-93.

250. Zhu J, Su L, Zhou Y, Qiao J, Hu W. The effect of nationwide quarantine on anxiety levels during the COVID-19 outbreak in China. Brain Behav. 2021;11:e01938.

251. Elhai JD, Yang H, McKay D, Asmundson GJG. COVID-19 anxiety symptoms associated with problematic smartphone use severity in Chinese adults. J Affect Disord. 2020;274:576-82.

252. Lau BHP, Chan CLW, Ng SM. Resilience of Hong Kong people in the COVID-19 pandemic: lessons learned from a survey at the peak of the pandemic in Spring 2020. Asia Pac J Soc Work. 2021;31:105-14.

253. Solomou I, Constantinidou F. Prevalence and predictors of anxiety and depression symptoms during the COVID-19 pandemic and compliance with precautionary measures: age and sex matter. Int J Environ Res Public Health. 2020;17:4924.

254. Bauerle A, Steinbach J, Schweda A, Beckord J, Hetkamp M, Weismuller B, et al. Mental health burden of the COVID-19 outbreak in Germany: predictors of mental health impairment. J Prim Care Community Health. 2020;11:2150132720953682.

255. Petzold MB, Bendau A, Plag J, Pyrkosch L, Maricic LM, Betzler F, et al. Risk, resilience, psychological distress, and anxiety at the beginning of the COVID-19 pandemic in Germany. Brain Behav. 2020;10:e01745.

256. Bäuerle A, Teufel M, Musche V, Weismüller B, Kohler H, Hetkamp M, et al. Increased generalized anxiety, depression and distress during the COVID-19 pandemic: a cross-sectional study in Germany. J Public Health. 2020;42:672-8.

257. Benke C, Autenrieth LK, Asselmann E, Pané-Farré CA. Lockdown, quarantine measures, and social distancing: associations with depression, anxiety and distress at the beginning of the COVID-19 pandemic among adults from Germany. Psychiatry Res. 2020;293:113462.

258. Hetkamp M, Schweda A, Bauerle A, Weismuller B, Kohler H, Musche V, et al. Sleep disturbances, fear, and generalized anxiety during the COVID-19 shut down phase in Germany: relation to infection rates, deaths, and German stock index DAX. Sleep Med. 2020;75:350-3.

259. Teufel M, Schweda A, Dörrie N, Musche V, Hetkamp M, Weismüller B, et al. Not all world leaders use Twitter in response to the COVID-19 pandemic: impact of the way of Angela Merkel on psychological distress, behaviour and risk perception. J Public Health. 2020;42:644-6.

260. Skoda E-M, Teufel M, Stang A, Jöckel K-H, Junne F, Weismüller B, et al. Psychological burden of healthcare professionals in Germany during the acute phase of the COVID-19 pandemic: differences and similarities in the international context. J Public Health. 2020;42:688-95.

261. Parlapani E, Holeva V, Voitsidis P, Blekas A, Gliatas I, Porfyri GN, et al. Psychological and behavioral responses to the COVID-19 pandemic in Greece. Front Psychiatry. 2020;11:821.

262. Papandreou C, Arija V, Aretouli E, Tsilidis KK, Bullo M. Comparing eating behaviours, and symptoms of depression and anxiety between Spain and Greece during the COVID-19 outbreak: cross-sectional analysis of two different confinement strategies. Eur Eat Disord Rev. 2020;28:836-46.

263. Parimala S, Kanchibhotla D. Association between yogic breathing practice with perceived impact of COVID-19: a cross-sectional study from India. Asia Pac J Public Health. 2021;33:157-9.

264. Gupta R, Grover S, Basu A, Krishnan V, Tripathi A, Subramanyam A, et al. Changes in sleep pattern and sleep quality during COVID-19 lockdown. Indian J Psychiatry. 2020;62:370-8.

265. Grover S, Sahoo S, Dua D, Mehra A, Nehra R. Psychological impact of COVID-19 duties during lockdown on police personnel and their perception about the behavior of the people: an exploratory study from India. Int J Ment Health Addict. 2022;20:831–42.

266. Shukla A. Psychological impact of COVID-19 lockdown: an online survey from India: few concerns. Indian J Psychiatry. 2020;62:591-2.

267. Mirhosseini S, Dadgari A, Basirinezhad MH, Mohammadpourhodki R, Ebrahimi H. The role of hope to alleviate anxiety in COVID-19 outbreak among community dwellers: an online cross-sectional survey. Ann Acad Med Singap. 2020;49:723-30.

268. Landi G, Pakenham KI, Boccolini G, Grandi S, Tossani E. Health anxiety and mental health outcome during COVID-19 lockdown in Italy: the mediating and moderating roles of psychological flexibility. Front Psychol. 2020;11:2195.

269. Pakenham KI, Landi G, Boccolini G, Furlani A, Grandi S, Tossani E. The moderating roles of psychological flexibility and inflexibility on the mental health impacts of COVID-19 pandemic and lockdown in Italy. J Contextual Behav Sci. 2020;17:109-18.

270. Massad I, Al-Taher R, Massad F, Al-Sabbagh MQ, Haddad M, Abufaraj M. The impact of the COVID-19 pandemic on mental health: early quarantine-related anxiety and its correlates among Jordanians. East Mediterr Health Journal. 2020;26:1165-72.

271. Gupta AK, Sahoo S, Mehra A, Grover S. Psychological impact of 'lockdown' due to COVID-19 pandemic in Nepal: an online survey. Asian J Psychiatr. 2020;54:102243.

272. Havnen A, Anyan F, Hjemdal O, Solem S, Riksfjord MG, Hagen K. Resilience moderates negative outcome from stress during the COVID-19 pandemic: a moderated-mediation approach. Int J Environ Res Public Health. 2020;17:6461.

273. Alhalafi AH. Prevalence of anxiety and depression during the Coronavirus Disease 2019 pandemic in Riyadh, Saudi Arabia: a web-based cross-sectional survey. J Pharm Res Int. 2020;32:65-73.

274. Lee SA, Crunk EA. Fear and psychopathology during the COVID-19 crisis: neuroticism, hypochondriasis, reassurance-seeking, and coronaphobia as fear factors. Omega. 2020. https://doi.org/10.1177/0030222820949350.

275. Ozdemir S, Ng S, Chaudhry I, Finkelstein EA. Adoption of preventive behaviour strategies and public perceptions about COVID-19 in Singapore. Int J Health Policy Manag. 2022;11:579-91.

276. González-Sanguino C, Ausín B, Castellanos MÁ, Saiz J, López-Gómez A, Ugidos C, et al. Mental health consequences during the initial stage of the 2020 Coronavirus pandemic (COVID-19) in Spain. Brain Behav Immun. 2020;87:172-6.

277. Fullana MA, Hidalgo-Mazzei D, Vieta E, Radua J. Coping behaviors associated with decreased anxiety and depressive symptoms during the COVID-19 pandemic and lockdown. J Affect Disord. 2020;275:80-1.

278. McCracken LM, Badinlou F, Buhrman M, Brocki KC. Psychological impact of COVID-19 in the Swedish population: depression, anxiety, and insomnia and their associations to risk and vulnerability factors. Eur Psychiatry. 2020;63:e81.

279. Ozdemir F, Cansel N, Kizilay F, Guldogan E, Ucuz I, Sinanoglu B, et al. The role of physical activity on mental health and quality of life during COVID-19 outbreak: a cross-sectional study. Eur J Integr Med. 2020;40:101248.

280. Özdin S, Bayrak Özdin Ş. Levels and predictors of anxiety, depression and health anxiety during COVID-19 pandemic in Turkish society: the importance of gender. Int J Soc Psychiatry. 2020;66:504-11.

281. Shevlin M, Nolan E, Owczarek M, McBride O, Murphy J, Gibson Miller J, et al. COVID-19-related anxiety predicts somatic symptoms in the UK population. Br J Health Psychol. 2020;25:875-82.

282. Dawson DL, Golijani-Moghaddam N. COVID-19: psychological flexibility, coping, mental health, and wellbeing in the UK during the pandemic. J Contextual Behav Sci. 2020;17:126-34.

283. Iob E, Steptoe A, Fancourt D. Abuse, self-harm and suicidal ideation in the UK during the COVID-19 pandemic. Br J Psychiatry. 2020;217:543-6.

284. Groarke JM, Berry E, Graham-Wisener L, McKenna-Plumley PE, McGlinchey E, Armour C. Loneliness in the UK during the COVID-19 pandemic: cross-sectional results from the COVID-19 Psychological Wellbeing Study. PLoS One. 2020;15:e0239698.

285. Pieh C, Budimir S, Delgadillo J, Barkham M, Fontaine JRJ, Probst T. Mental health during COVID-19 lockdown in the United Kingdom. Psychosom Med. 2021;83:328-37.

286. Rettie H, Daniels J. Coping and tolerance of uncertainty: predictors and mediators of mental health during the COVID-19 pandemic. Am Psychol. 2021;76:427-37.

287. Meyer J, McDowell C, Lansing J, Brower C, Smith L, Tully M, et al. Changes in physical activity and sedentary behavior in response to COVID-19 and their associations with mental health in 3052 US adults. Int J Environ Res Public Health. 2020;17:6469.

288. Lee SA, Jobe MC, Mathis AA, Gibbons JA. Incremental validity of coronaphobia: coronavirus anxiety explains depression, generalized anxiety, and death anxiety. J Anxiety Disord. 2020;74:102268.

289. Fitzpatrick KM, Harris C, Drawve G. Fear of COVID-19 and the mental health consequences in America. Psychol Trauma. 2020;12:S17-21.

290. Passos L, Prazeres F, Teixeira A, Martins C. Impact on mental health due to COVID-19 pandemic: cross-sectional study in Portugal and Brazil. Int J Environ Res Public Health. 2020;17:6794.

291. Taylor S, Landry CA, Paluszek MM, Fergus TA, McKay D, Asmundson GJG. COVID stress syndrome: concept, structure, and correlates. Depress Anxiety. 2020;37:706-14.

292. Nelson BW, Pettitt A, Flannery JE, Allen NB. Rapid assessment of psychological and epidemiological correlates of COVID-19 concern, financial strain, and health-related behavior change in a large online sample. PLoS One. 2020;15:e0241990.

293. Alzueta E, Perrin P, Baker FC, Caffarra S, Ramos-Usuga D, Yuksel D, et al. How the COVID-19 pandemic has changed our lives: a study of psychological correlates across 59 countries. J Clin Psychol. 2021;77:556-70.

294. Généreux M, Schluter PJ, Hung KK, Wong CS, Pui Yin Mok C, O'Sullivan T, et al. One virus, four continents, eight countries: an interdisciplinary and international study on the psychosocial impacts of the COVID-19 pandemic among adults. Int J Environ Res Public Health. 2020;17:8390.

295. Prout TA, Zilcha-Mano S, Aafjes-van Doorn K, Bekes V, Christman-Cohen I, Whistler K, et al. Identifying predictors of psychological distress during COVID-19: a machine learning approach. Front Psychol. 2020;11:586202.

296. Lee SA, Jobe MC, Mathis AA. Mental health characteristics associated with dysfunctional coronavirus anxiety. Psychol Med. 2021;51:1403-4.

297. Ng DWL, Chan FHF, Barry TJ, Lam C, Chong CY, Kok HCS, et al. Psychological distress during the 2019 Coronavirus Disease (COVID-19) pandemic among cancer survivors and healthy controls. Psychooncology. 2020;29:1380-3.

298. Salari M, Etemadifar M, Gharagozli K, Etemad K, Ashrafi F, Ashourizadeh H. Incidence of anxiety in epilepsy during coronavirus disease (COVID-19) pandemic. Epilepsy Behav. 2020;112:107442.

299. Schmitz N, Holley P, Meng XF, Fish L, Jedwab J. COVID-19 and depressive symptoms: a community-based study in Quebec, Canada. Can J Psychiatry. 2020;65:733-5.

300. Sønderskov KM, Dinesen PT, Santini ZI, Østergaard SD. The depressive state of Denmark during the COVID-19 pandemic. Acta Neuropsychiatr. 2020;32:226-8.

301. Liu Z, Zhang X, Lü Z, Liang J, Deng Y, Feng L. Mental health status and its influencing factors among general population and medical personnel in Guangdong Province during COVID-19 pandemic. Nan Fang Yi Ke Da Xue Xue Bao. 2020;40:1530-8.

302. Peng M, Mo BR, Liu YS, Xu MM, Song XR, Liu LY, et al. Prevalence, risk factors and clinical correlates of depression in quarantined population during the COVID-19 outbreak. J Affect Disord. 2020;275:119-24.

303. Jiang W, Liu X, Zhang J, Feng Z. Mental health status of Chinese residents during the COVID-19 epidemic. BMC Psychiatry. 2020;20:580.

304. Liu X, Luo WT, Li Y, Li CN, Hong ZS, Chen HL, et al. Psychological status and behavior changes of the public during the COVID-19 epidemic in China. Infect Dis Poverty. 2020;9:58.

305. Chen B, Li QX, Zhang H, Zhu JY, Yang X, Wu YH, et al. The psychological impact of COVID-19 outbreak on medical staff and the general public. Curr Psychol. 2020. https://doi.org/10.1007/s12144-020-01109-0.

306. Voitsidis P, Nikopoulou VA, Holeva V, Parlapani E, Sereslis K, Tsipropoulou V, et al. The mediating role of fear of COVID-19 in the relationship between intolerance of uncertainty and depression. Psychol Psychother. 2021;94:884-93.

307. Singh SP, Khokhar A. Prevalence of posttraumatic stress disorder and depression in general population in India during COVID-19 pandemic home quarantine. Asia Pac J Public Health. 2021;33:154-6.

308. Shapiro E, Levine L, Kay A. Mental health stressors in Israel during the coronavirus pandemic. Psychol Trauma. 2020;12:499-501.

309. Sugaya N, Yamamoto T, Suzuki N, Uchiumi C. A real-time survey on the psychological impact of mild lockdown for COVID-19 in the Japanese population. Sci Data. 2020;7:372.

310. Stickley A, Matsubayashi T, Ueda M. Loneliness and COVID-19 preventive behaviours among Japanese adults. J Public Health. 2021;43:53-60.

311. Ghimire C, Acharya S, Shrestha C, Prabhat KC, Singh S, Sharma P. Interpersonal violence during the COVID-19 lockdown period in Nepal: a descriptive cross-sectional study. JNMA J Nepal Med Assoc. 2020;58:751-7.

312. Shrestha C, Ghimire C, Acharya S, Prabhat KC, Singh S, Sharma P. Mental wellbeing during the lockdown period following the COVID-19 pandemic in Nepal: a descriptive cross-sectional study. JNMA J Nepal Med Assoc. 2020;58:744-50.

313. Bodecka M, Nowakowska I, Zajenkowska A, Rajchert J, Kazmierczak I, Jelonkiewicz I. Gender as a moderator between Present-Hedonistic time perspective and depressive symptoms or stress during COVID-19 lock-down. Pers Individ Dif. 2021;168:110395.

314. Kim AW, Nyengerai T, Mendenhall E. Evaluating the mental health impacts of the COVID-19 pandemic: perceived risk of COVID-19 infection and childhood trauma predict adult depressive symptoms in urban South Africa. Psychol Med. 2020. https://doi.org/10.1017/S0033291720003414.

315. Lee JJ, Kang KA, Wang MP, Zhao SZ, Wong JYH, O'Connor S, et al. Associations between COVID-19 misinformation exposure and belief with COVID-19 knowledge and preventive behaviors: cross-sectional online study. J Med Internet Res. 2020;22:e22205.

316. Kim YJ, Cho JH, Kim ES. Differences in sense of belonging, pride, and mental health in the Daegu Metropolitan Region due to COVID-19: comparison between the presence and absence of national disaster relief fund. Int J Environ Res Public Health. 2020;17:4910.

317. Karasar B, Canli D. Psychological resilience and depression during the COVID-19 pandemic in Turkey Psychiatr Danub. 2020;32:273-9.

318. Ustun G. Determining depression and related factors in a society affected by COVID-19 pandemic. Int J Soc Psychiatry. 2021;67:54-63.

319. Knell G, Robertson MC, Dooley EE, Burford K, Mendez KS. Health behavior changes during COVID-19 pandemic and subsequent “stay-at-home" orders. Int J Environ Res Public Health. 2020;17:6268.

320. Planchuelo-Gomez A, Odriozola-Gonzalez P, Irurtia MJ, de Luis-Garcia R. Longitudinal evaluation of the psychological impact of the COVID-19 crisis in Spain. J Affect Disord. 2020;277:842-9.

321. Shuwiekh HAM, Kira IA, Sous MSF, Ashby JS, Alhuwailah A, Baali SBA, et al. The differential mental health impact of COVID-19 in Arab countries. Curr Psychol. 2020. https://doi.org/10.1007/s12144-020-01148-7.

322. Gurvich C, Thomas N, Thomas EH, Hudaib AR, Sood L, Fabiatos K, et al. Coping styles and mental health in response to societal changes during the COVID-19 pandemic. Int J Soc Psychiatry. 2020;67:540-9.

323. Traunmuller C, Stefitz R, Gaisbachgrabner K, Schwerdtfeger A. Psychological correlates of COVID-19 pandemic in the Austrian population. BMC Public Health. 2020;20:1395.

324. Cao Y, Ma ZF, Zhang YT, Zhang YF. Evaluation of lifestyle, attitude and stressful impact amid COVID-19 among adults in Shanghai, China. Int J Environ Health Res. 2022;32:1137-46.

325. Ma ZF, Luo X, Li X, Li Y, Liu S, Zhang Y. Increased stressful impact among general population in mainland China amid the COVID-19 pandemic: a nationwide cross-sectional study conducted after Wuhan city's travel ban was lifted. Int J Soc Psychiatry. 2020;66:770-9.

326. Zhang Y, Ma ZF. Impact of the COVID-19 pandemic on mental health and quality of life among local residents in Liaoning province, China: a cross-sectional study. Int J Environ Res Public Health. 2020;17:2381.

327. Wang CY, Pan RY, Wan XY, Tan YL, Xu LK, Ho CS, et al. Immediate psychological responses and associated factors during the initial stage of the 2019 Coronavirus Disease (COVID-19) epidemic among the general population in China. Int J Environ Res Public Health. 2020;17:1729.

328. Jiang WY, Ren ZH, Yu LX, Tan YF, Shi CR. A network analysis of post-traumatic stress disorder symptoms and correlates during the COVID-19 pandemic. Front Psychiatry. 2020;11:568037.

329. Liu N, Zhang F, Wei C, Jia Y, Shang Z, Sun L, et al. Prevalence and predictors of PTSS during COVID-19 outbreak in China hardest-hit areas: gender differences matter. Psychiatry Res. 2020;287:112921.

330. El-Zoghby SM, Soltan EM, Salama HM. Impact of the COVID-19 pandemic on mental health and social support among adult Egyptians. J Community Health. 2020;45:689-95.

331. Ahuja P, Syal G, Kaur A. Psychological stress: repercussions of COVID-19 on gender. J Public Aff. 2021;21:e2533.

332. Varshney M, Parel JT, Raizada N, Sarin SK. Initial psychological impact of COVID-19 and its correlates in Indian community: an online (FEEL-COVID) survey. PLoS One. 2020;15:e0233874.

333. Lahav Y. Psychological distress related to COVID-19—the contribution of continuous traumatic stress. J Affect Disord. 2020;277:129-37.

334. Di Giuseppe M, Zilcha-Mano S, Prout TA, Perry JC, Orru G, Conversano C. Psychological impact of Coronavirus Disease 2019 among Italians during the first week of lockdown. Front Psychiatry. 2020;11:576597.

335. Micarelli A, Granito I, Carlino P, Micarelli B, Alessandrini M. Self-perceived general and ear-nose-throat symptoms related to the COVID-19 outbreak: a survey study during quarantine in Italy. J Int Med Res. 2020;48:300060520961276.

336. Forte G, Favieri F, Tambelli R, Casagrande M. COVID-19 pandemic in the Italian population: validation of a post-traumatic stress disorder questionnaire and prevalence of PTSD symptomatology. Int J Environ Res Public Health. 2020;17:4151.
337. Cortes-Alvarez NY, Pineiro-Lamas R, Vuelvas-Olmos CR. Psychological effects and associated factors of COVID-19 in a Mexican sample. Disaster Med Public Health Prep. 2020;14:413-24.
338. Tee ML, Tee CA, Anlacan JP, Aligam KJG, Reyes PWC, Kuruchittham V, et al. Psychological impact of COVID-19 pandemic in the Philippines. J Affect Disord. 2020;277:379-91.
339. Paulino M, Dumas-Diniz R, Brissos S, Brites R, Alho L, Simoes MR, et al. COVID-19 in Portugal: exploring the immediate psychological impact on the general population. Psychol Health Med. 2021;26:44-55.
340. Alkhamees AA, Alrashed SA, Alzunaydi AA, Almohimeed AS, Aljohani MS. The psychological impact of COVID-19 pandemic on the general population of Saudi Arabia. Compr Psychiatry. 2020;102:152192.
341. Odriozola-Gonzalez P, Planchuelo-Gomez A, Irurtia MJ, de Luis-Garcia R. Psychological symptoms of the outbreak of the COVID-19 confinement in Spain. J Health Psychol. 2022;27:825-35.
342. Rodriguez-Rey R, Garrido-Hernansaiz H, Collado S. Psychological impact and associated factors during the initial stage of the Coronavirus (COVID-19) pandemic among the general population in Spain. Front Psychol. 2020;11:1540.
343. Rodríguez-Rey R, Garrido-Hernansaiz H, Collado S. Psychological impact of COVID-19 in Spain: early data report. Psychol Trauma. 2020;12:550-2.
344. Le XTT, Dang AK, Toweh J, Nguyen QN, Le HT, Do TTT, et al. Evaluating the psychological impacts related to COVID-19 of Vietnamese people under the first nationwide partial lockdown in Vietnam. Front Psychiatry. 2020;11:824.
345. Hao F, Tan W, Jiang L, Zhang L, Zhao X, Zou Y, et al. Do psychiatric patients experience more psychiatric symptoms during COVID-19 pandemic and lockdown? A case-control study with service and research implications for immunopsychiatry. Brain Behav Immun. 2020;87:100-6.

346. Guo Q, Zheng Y, Shi J, Wang J, Li G, Li C, et al. Immediate psychological distress in quarantined patients with COVID-19 and its association with peripheral inflammation: a mixed-method study. Brain Behav Immun. 2020;88:17-27.

347. Sim K, Chan YH, Chong PN, Chua HC, Soon SW. Psychosocial and coping responses within the community health care setting towards a national outbreak of an infectious disease. J Psychosom Res. 2010;68:195-202.

348. Sibley CG, Greaves LM, Satherley N, Wilson MS, Overall NC, Lee CHJ, et al. Effects of the COVID-19 pandemic and nationwide lockdown on trust, attitudes toward government, and well-being. Am Psychol. 2020;75:618-30.

349. Nicolson MN, Flett JA. The mental wellbeing of New Zealanders during and post-lockdown. N Z Med J. 2020;133:110-2.

350. Gray NS, O'Connor C, Knowles J, Pink J, Simkiss NJ, Williams SD, et al. The influence of the COVID-19 pandemic on mental well-being and psychological distress: impact upon a single country. Front Psychiatry. 2020;11:594115.

351. Twenge JM, Joiner TE. Mental distress among US adults during the COVID-19 pandemic. J Clin Psychol. 2020;76:2170-82.

352. Fernandez RS, Crivelli L, Guimet NM, Allegri RF, Pedreira ME. Psychological distress associated with COVID-19 quarantine: latent profile analysis, outcome prediction and mediation analysis. J Affect Disord. 2020;277:75-84.

353. Yu H, Li M, Li Z, Xiang W, Yuan Y, Liu Y, et al. Coping style, social support and psychological distress in the general Chinese population in the early stages of the COVID-19 epidemic. BMC Psychiatry. 2020;20:426.

354. Goodwin R, Hou WK, Sun SJ, Ben-Ezra M. Quarantine, distress and interpersonal relationships during COVID-19. Gen Psychiatr. 2020;33:e100385.

355. Tian F, Li H, Tian S, Yang J, Shao J, Tian C. Psychological symptoms of ordinary Chinese citizens based on SCL-90 during the level I emergency response to COVID-19. Psychiatry Res. 2020;288:112992.

356. Lau BHP, Chan CLW, Ng SM. Self-compassion buffers the adverse mental health impacts of COVID-19-related threats: results from a cross-sectional survey at the first peak of Hong Kong's outbreak. Front Psychiatry. 2020;11:585270.

357. El-Abasiri RA, Marzo RR, Abdelaziz H, Boraii S, Abdelaziz DH. Evaluating the psychological distress of the coronavirus disease 2019 pandemic in Egypt. Eur J Mol Clin Med. 2020;7:1-12.

358. Liu S, Heinz A. Cross-cultural validity of psychological distress measurement during the coronavirus pandemic. Pharmacopsychiatry. 2020;53:237-8.

359. Venugopal VC, Mohan A, Chennabasappa LK. Status of mental health and its associated factors among the general populace of India during COVID-19 pandemic. Asia Pac Psychiatry. 2020. https://doi.org/10.1111/appy.12412.

360. Jahanshahi AA, Dinani MM, Madavani AN, Li JZ, Zhang SX. The distress of Iranian adults during the COVID-19 pandemic - more distressed than the Chinese and with different predictors. Brain Behav Immun. 2020;87:124-5.

361. Fiorillo A, Sampogna G, Giallonardo V, Del Vecchio V, Luciano M, Albert U, et al. Effects of the lockdown on the mental health of the general population during the COVID-19 pandemic in Italy: results from the COMET collaborative network. Eur Psychiatry. 2020;63:e87.

362. Moccia L, Janiri D, Pepe M, Dattoli L, Molinaro M, De Martin V, et al. Affective temperament, attachment style, and the psychological impact of the COVID-19 outbreak: an early report on the Italian general population. Brain Behav Immun. 2020;87:75-9.

363. Shrestha DB, Thapa BB, Katuwal N, Shrestha B, Pant C, Basnet B, et al. Psychological distress in Nepalese residents during COVID-19 pandemic: a community level survey. BMC Psychiatry. 2020;20:491.

364. Perez S, Masegoso A, Hernandez-Espeso N. Levels and variables associated with psychological distress during confinement due to the coronavirus pandemic in a community sample of Spanish adults. Clin Psychol Psychother. 2021;28:606-14.

365. Schnell T, Krampe H. Meaning in life and self-control buffer stress in times of COVID-19: moderating and mediating effects with regard to mental distress. Front Psychiatry. 2020;11:582352.

366. Chodkiewicz J, Talarowska M, Miniszewska J, Nawrocka N, Bilinski P. Alcohol consumption reported during the COVID-19 pandemic: the initial stage. Int J Environ Res Public Health. 2020;17:4677.

367. Gratz KL, Tull MT, Richmond JR, Edmonds KA, Scamaldo K, Rose JP. Thwarted belongingness and perceived burdensomeness explain the associations of COVID-19 social and economic consequences to suicide risk. Suicide Life Threat Behav. 2020;50:1140-8.

368. Fitzpatrick KM, Harris C, Drawve G. How bad is it? Suicidality in the middle of the COVID-19 pandemic. Suicide Life Threat Behav. 2020;50:1241-9.

369. Dai HY, Zhang SX, Looi KH, Su R, Li JZ. Perception of health conditions and test availability as predictors of adults' mental health during the COVID-19 pandemic: a survey study of adults in Malaysia. Int J Environ Res Public Health. 2020;17:5498.

370. Grey I, Arora T, Thomas J, Saneh A, Tohme P, Abi-Habib R. The role of perceived social support on depression and sleep during the COVID-19 pandemic. Psychiatry Res. 2020;293:113452.

371. Lee SA. Coronavirus Anxiety Scale: a brief mental health screener for COVID-19 related anxiety. Death Stud. 2020;44:393-401.

372. Zhang Y, Zhang H, Ma X, Di Q. Mental health problems during the COVID-19 pandemics and the mitigation effects of exercise: a longitudinal study of college students in China. Int J Environ Res Public Health. 2020;17:3722.
