# Supplementary figures and images for: Mental disorders following COVID-19 and other epidemics: a systematic review and meta-analysis
Source: Transl Psychiatry. 2022 May 17;12:205. doi: 10.1038/s41398-022-01946-6 (PMC9110635; doi:10.1038/s41398-022-01946-6)

**Figure 1. PRISMA flowchart**

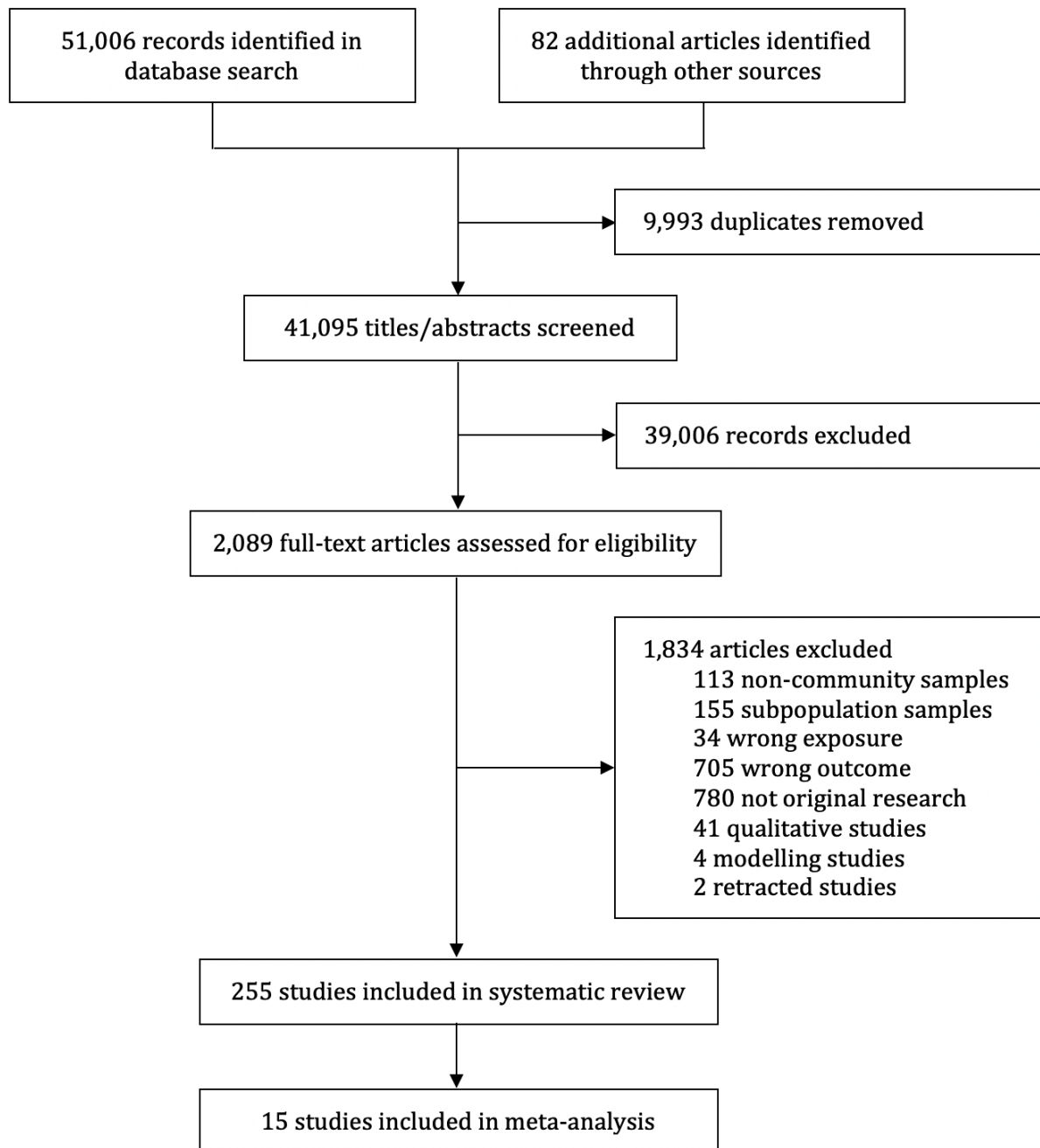

Supplement: Supplementary file 2 — PRISMA Flowchart [file 41398_2022_1946_MOESM2_ESM.pdf]
